# From Quinoxaline, Pyrido[2,3-*b*]pyrazine and Pyrido[3,4-*b*]pyrazine to Pyrazino-Fused Carbazoles and Carbolines

**DOI:** 10.3390/molecules23112961

**Published:** 2018-11-13

**Authors:** Frédéric Lassagne, Timothy Langlais, Elsa Caytan, Emmanuelle Limanton, Ludovic Paquin, Manon Boullard, Coline Courtel, Idriss Curbet, Clément Gédéon, Julien Lebreton, Laurent Picot, Valérie Thiéry, Mohamed Souab, Blandine Baratte, Sandrine Ruchaud, Stéphane Bach, Thierry Roisnel, Florence Mongin

**Affiliations:** 1 Univ Rennes, CNRS, Institut des Sciences Chimiques de Rennes (ISCR)-UMR 6226, F-35000 Rennes, France; timothy.langlais@etudiant.univ-rennes1.fr (T.L.); elsa.caytan@univ-rennes1.fr (E.C.); emmanuelle.limanton@univ-rennes1.fr (E.L.); manon.boullard@etudiant.univ-rennes1.fr (M.B.); coline.courtel@etudiant.univ-rennes1.fr (C.C.); idriss.curbet@etudiant.univ-rennes1.fr (I.C.); clement.gedeon@etudiant.univ-rennes1.fr (C.G.); julien.lebreton@etudiant.univ-rennes1.fr (J.L.); thierry.roisnel@univ-rennes1.fr (T.R.); 2 Laboratoire Littoral Environnement et Sociétés, UMRi CNRS 7266, Université de La Rochelle, 17042 La Rochelle, France; valerie.thiery@univ-lr.fr; 3 Sorbonne Universités, UPMC Univ Paris 06, CNRS USR3151, “Protein Phosphorylation and Human Disease” Unit, Plateforme de criblage KISSf, Station Biologique de Roscoff, Place Georges Teissier, 29688 Roscoff, France; mo.souab@gmail.com (M.S.); baratte@sb-roscoff.fr (B.B.); sandrine.ruchaud@sb-roscoff.fr (S.R.)

**Keywords:** pyrazine, deprotometalation, coupling, *N*-arylation, palladium, copper

## Abstract

2,3-Diphenylated quinoxaline, pyrido[2,3-*b*]pyrazine and 8-bromopyrido[3,4-*b*]pyrazine were halogenated in deprotometalation-trapping reactions using mixed 2,2,6,6-tetramethyl piperidino-based lithium-zinc combinations in tetrahydrofuran. The 2,3-diphenylated 5-iodo- quinoxaline, 8-iodopyrido[2,3-*b*]pyrazine and 8-bromo-7-iodopyrido[3,4-*b*]pyrazine thus obtained were subjected to palladium-catalyzed couplings with arylboronic acids or anilines, and possible subsequent cyclizations to afford the corresponding pyrazino[2,3-*a*]carbazole, pyrazino[2′,3′:5,6] pyrido[4,3-*b*]indole and pyrazino[2′,3′:4,5]pyrido[2,3-*d*]indole, respectively. 8-Iodopyrido[2,3-*b*] pyrazine was subjected either to a copper-catalyzed C-N bond formation with azoles, or to direct substitution to introduce alkylamino, benzylamino, hydrazine and aryloxy groups at the 8 position. The 8-hydrazino product was converted into aryl hydrazones. Most of the compounds were evaluated for their biological properties (antiproliferative activity in A2058 melanoma cells and disease-relevant kinase inhibition).

## 1. Introduction

Quinoxalines and pyridopyrazines are aromatic heterocycles present in compounds endowed with numerous interesting properties. Some derivatives are bioactive and are used as antimicrobial, anti-inflammatory, antimalarial, anticancer and antidepressant compounds [1,2]. Others are for example employed as organic dyes [3], electroluminescent materials [4], and organic semiconductors [5]. Quinoxaline and pyridopyrazine substrates can be readily synthesized by condensation of 1,2-dicarbonyl compounds with 1,2-arylenediamines [6] and lend themselves to further elaboration.

Deprotonative lithiation followed by interception of the arylmetals with electrophiles is an efficient way to functionalize aromatic compounds [7,8,9,10,11,12]. However, reactions with substrates sensitive to nucleophilic attack such as azines must be performed at very low temperatures to avoid secondary reactions between arylmetals and functions [13,14,15]. The use of in situ metal traps avoids the use of cryogenic conditions to achieve these reactions [16,17]. We have developed mixed lithium-zinc combinations based on TMP (TMP = 2,2,6,6-tetramethylpiperidino) capable of deprotonating sensitive substrates at temperatures close to rt [18,19,20,21]. In order to obtain original scaffolds such as pyrazino-fused carbazoles and carbolines, we decided to combine this deprotometalation under *in situ* trapping conditions with palladium- and copper-catalyzed coupling reactions.

## 2. Results and Discussion

### 2.1. Synthesis

To functionalize 2,3-diphenylquinoxaline (**1a**) and 2,3-diphenylpyrido[2,3-*b*]pyrazine (**2a**), two deprotonation methods were tested in tetrahydrofuran (THF) (Table 1, *Method A* and *Method B*).

The lithium-zinc base of *Method A* is prepared from ZnCl_2_·TMEDA (TMEDA = *N*,*N*,*N′*,*N′*- tetramethylethylenediamine) and LiTMP in a 1:3 ratio. Previous studies have suggested that it is a 1:1 LiTMP-Zn(TMP)_2_ combination. While LiTMP deprotonates the substrate, Zn(TMP)_2_ intercepts the generated aryllithium [18,19,22]. A recent computer study on anisole showed that the reactive species is solvated LiTMP. The effectiveness of the reaction derives from the stabilizing effect of the transmetalation step [21].

It is possible to replace Zn(TMP)_2_ by ZnCl_2_ provided that there is no contact between LiTMP and ZnCl_2_ in the absence of the aromatic compound [23,24]. Thus, *Method B* is limited to activated substrates for which deprotonation is favored over reaction between LiTMP and ZnCl_2_.

Whereas *Method A* should provide a lithium arylzincate, *Method B* should rather generate an arylzinc. Nevertheless, both species are known to react with iodine by aryl transfer.

Thus, 2,3-diphenylquinoxaline (**1a**) and 2,3-diphenylpyrido[2,3-*b*]pyrazine (**2a**) were involved in *Method A*. After treatment at rt with the base for 2 h, addition of iodine led to iodoquinoxaline **1b** and iodopyrido[2,3-*b*] pyrazine **2b-I** in 74 and 70% yield, respectively (entries 1 and 3).

To evaluate *Method B*, **1a** and **2a** were mixed with ZnCl_2_·TMEDA before addition of LiTMP at −20 °C and stirring for 0.5 h (*Method B*, entries 2 and 4). After subsequent interception with iodine, **1b** and **2b-I** were isolated in 70 and 62% yield, respectively (entries 2 and 4).

We explored the use of other electrophiles to intercept the heteroarylzinc chloride prepared from **2a** by using *Method B*. Conversion to the corresponding bromide **2b-Br** (60% yield, entry 5) and chloride **2b-Cl** (62% yield, entry 6) was performed using bromine and trichloroisocyanuric acid, respectively, as the electrophile.

**Figure 1:**
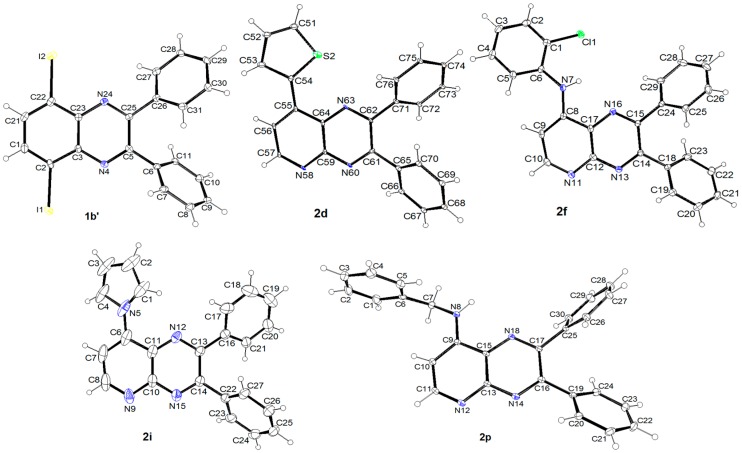
ORTEP diagrams (30% probability) of **1b′**, **2d**, **2f**, **2i**, **2p**.

**Scheme 1:**
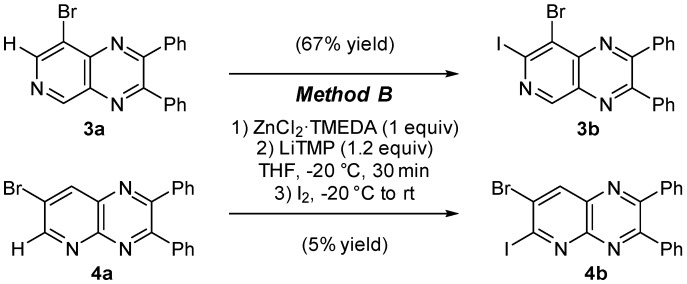
Deprotonative metalation of 8-bromo-2,3-diphenylpyrido[3,4-*b*]pyrazine (**3a**) and 7-bromo-2,3-diphenylpyrido[2,3-*b*]pyrazine (**4a**) followed by conversion to the halogeno derivatives.

The deprotometalation-iodination sequence was successfully applied to 8-bromo-2,3-diphenyl pyrido[3,4-*b*]pyrazine (**3a**) [25,26], but failed with 7-bromo-2,3-diphenylpyrido[2,3-*b*]pyrazine (**4a**) due to significant degradation before trapping (Scheme 1). While the position of the iodo group in **3b** was evidenced by subsequent reaction, it was studied by advanced NMR experiments in the case of **4b** (see Supplementary Materials).

In order to prepare original pyrazino-fused carbazoles and carbolines, iodides **1b** and **2b-I** were subjected to in Suzuki couplings [27,28] under standard conditions (Table 2) [29]. Phenyl- (entry 1), 2-thienyl- (entries 2 and 3) and 2-aminophenyl- (entries 4 and 5) boronic acids led to the 5-arylated derivatives **1c**-**e** and **2d**,**e** in 42-97% yields. The more electron-rich arylboronic acids and the less electron-poor quinoxaline substrate **1b** gave the best results.

**Scheme 2:**
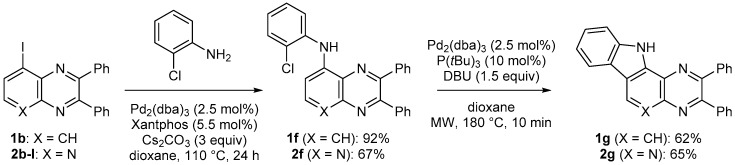
Conversion of 5-iodo-2,3-diphenylquinoxaline (**1b**) and 8-iodo-2,3-diphenylpyrido[2,3-*b*] pyrazine (**2b-I**) into 2,3-diphenyl-11*H*-pyrazino[2,3-*a*]carbazole (**1g**) and 2,3-diphenyl-11*H*-pyrazino [2′,3′:5,6]pyrido[4,3-*b*]indole (**2g**), respectively. Dba = dibenzylideneacetone.

No intramolecular nitrene insertion into the corresponding diazino-fused carbazole and β-carboline was obtained for the azides coming from **1e** and **2e** [29]. We thus turned to the synthesis of the original pyrazino[2,3-*a*]carbazole **1g** and the corresponding pyrazino-fused γ-carboline **2g** isomers by combining intermolecular C-N bond formation [30,31,32,33,34,35,36,37,38] with intramolecular C-C bond formation (Scheme 2).

The first step, attempted from **1b** by using catalytic palladium(II) acetate as transition metal source, Xantphos as ligand, and sodium *tert*-butoxide as base in toluene [39], yielded only 16% of diarylamine **1f**. Applying to **1b** and **2b-I** the conditions reported by Maes and co-workers for related reactions [29], **1f** and **2f** were obtained in 92 and 67% yield, respectively (Scheme 2, left). Inspired by Pieters and co-workers, who cyclized 4-(2-chlorophenylamino)pyridine into 5*H*-pyrido[4,3-*b*]indole under these conditions [40], we successfully employed catalytic (Pd_2_(dba)_3_) and tri-*tert*-butylphosphine as catalyst precursors, diazabicyclo[5.4.0]undec-7-ene (DBU) as base, and dioxane as solvent for the second step. After 10 min at 180 °C under microwave irradiation, the pyrazino-fused carbazole **1g** and γ-carboline **2g** were isolated in moderate yields (Scheme 2, right).

We decided to combine both steps in an auto-tandem process under microwave irradiation (Table 3). Using (Pd_2_(dba)_3_), we selected Xantphos for its higher efficiency in comparison with tri-*tert*-butylphosphine. From **2b**, best results were obtained with three equivalents of DBU as base (entries 1 and 2). In addition, a longer reaction time was required to ensure complete conversion and this afforded carboline **2g** in 70% yield (entry 3).

By testing a profile to maximize the microwave power, we noticed that an increase of the applied power favored the formation of **2f** over **2g** (entry 4). By carrying out one third of the reaction time under microwave irradiation and the rest by classical heating at the same temperature, a small microwave effect was evidenced (entry 5). While **2g** was not formed without catalyst, C-N bond formation giving **2f** could take place (entry 6; see Figure 1). However, increasing the catalyst amount had no impact on the conversion to **2g** (entry 7). Finally, we intentionally chose a short reaction time (5 min) in order to compare the palladium-catalyzed reactions under microwave irradiation from **2b-I** (entry 7), **2b-Br** (entry 7) and **2b-Cl** (entry 10). The results clearly showed decreasing reactivity from **2b-I** to **2b-Cl**, and thus, we selected iodo as halogeno group to pursue our investigations.

**Scheme 3:**
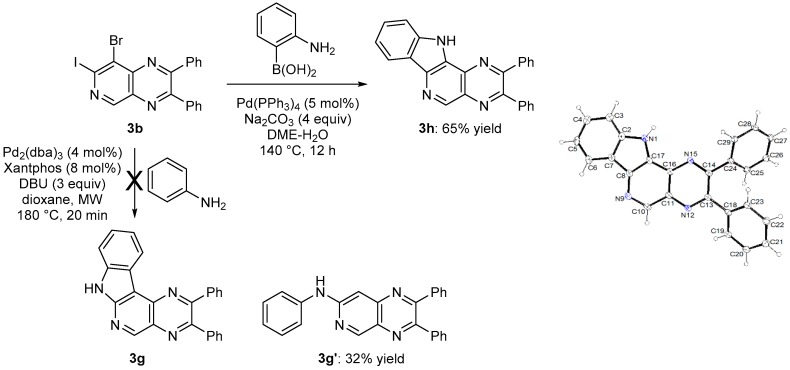
Conversion of 8-bromo-7-iodo-2,3-diphenylpyrido[3,4-*b*]pyrazine (**3b**) into 2,3-diphenyl-11*H*-pyrazino[2′,3′:4,5]pyrido[2,3-*d*]indole (**3h**) and ORTEP diagram (30% probability) of **3h**.

We applied the optimized procedure to the synthesis of the pyrazino-fused α-carboline **3g** from the bromoiodo substrate **3b** and aniline. No trace of the expected product **3g** was detected but the formation of **3g′** due to competitive debromination was noted, showing a less obvious intramolecular C-H arylation (Scheme 3, left). Consequently, we moved to the synthesis of the pyrazino-fused δ-carboline **3h**. Upon treatment of **3b** by 2-aminophenylboronic acid under standard conditions [29], coupling and subsequent cyclization occurred, providing **3h** in 65% yield (Scheme 3, right).

To take advantage of the iodo group on **2b-I**, C-N bond formation with azoles was attempted under copper catalysis as reported previously [41,42] (Table 4). Thus, by treating **2b-I** with pyrrole (entry 1; see Figure 1), indole (entry 2), pyrazole (entry 3), imidazole (entry 4) or 1,2,4-triazole (entry 5), in the presence of catalytic copper(I) oxide, cesium carbonate, and dimethylsulfoxide (DMSO) at 110 °C for 24 h, the expected *N*-arylated azoles were obtained in 51 to 79% yields.

**Scheme 4:**
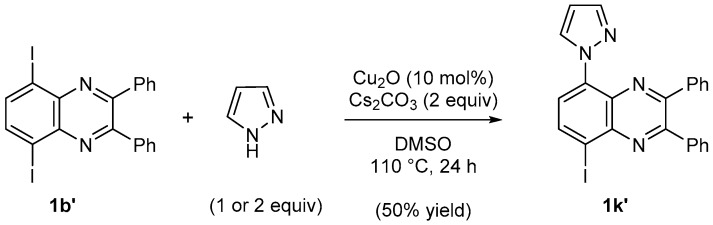
Copper-catalyzed *N*-arylation of 5,8-diiodo-2,3-diphenylquinoxaline (**1b′**).

As previously mentioned [22], such reactions work far less efficiently when performed on diiodides. Indeed, reacting the diiodide **1b′** with pyrazole only gave the monofunctionalized derivative **1k′**, regardless of the amount of azole employed (Scheme 4).

**Scheme 5:**
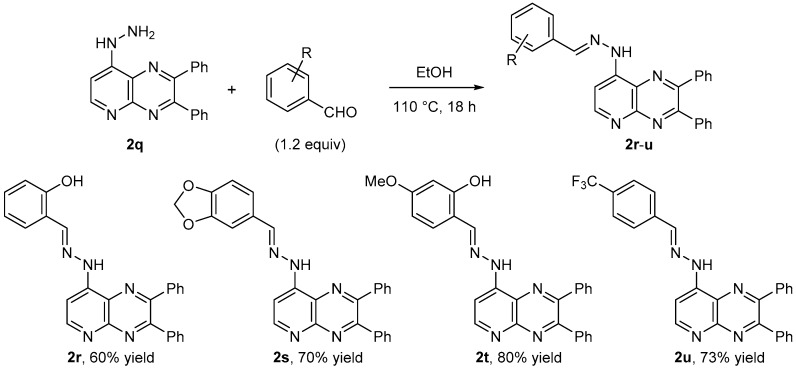
Conversion of 8-hydrazino-2,3-diphenylpyrido[2,3-*b*]pyrazine (**2q**) into aryl hydrazones.

**Scheme 6:**
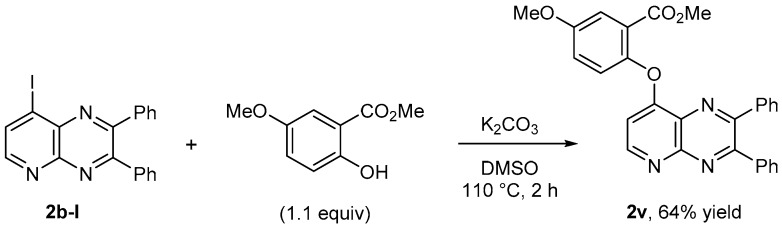
Conversion of 8-iodo-2,3-diphenylpyrido[2,3-*b*]pyrazine (**2b-I**) into ether **2v**.

Different amines and hydrazine reacted with **2b-I** without recourse to catalyst (Table 5), affording the corresponding secondary amines **2n**-**p** (entries 1-3) and arylhydrazine **2q** (entry 4) in good yields. The latter was converted into the hydrazones **2r**-**u** in the presence of aromatic aldehydes chosen for their ability to potentially interact with binding sites of biological interest [43] (Scheme 5). Finally, reaction of **2b**-**I** with a phenol also proved possible without catalyst, giving the diaryl ether **2v** in 64% yield (Scheme 6).

### 2.2. Biological Activity

**Figure 2:**
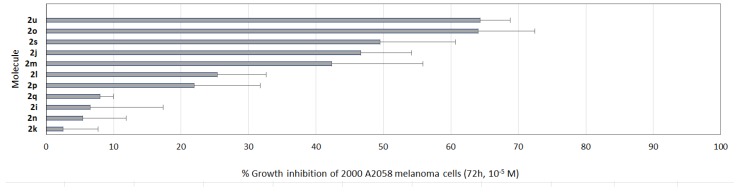
Antiproliferative activity of some of the synthesized compounds at 10^−5^ M after 72 h in A2058 human melanoma cells.

Some of the synthesized compounds were tested [44] for their antiproliferative activity in A2058 melanoma cells and proved to exert a modest to good activity (Figure 2). The best results were obtained with the 4-(trifluoromethyl)benzaldehyde hydrazone **2u** and the 8-benzylamino pyrido[2,3-*b*]pyrazine **2o** which induced ~64% growth inhibition at 10^−5^ M.

Compounds **1c**–**e**, **1g**, **2d**–**g**, **2i**–**v** and **3h** were evaluated [44] against a short panel of disease-relevant protein kinases. Protein kinases are drug targets often deregulated in diseases such as cancers and neurodegenerative disorders [45]. No significant inhibition of the following kinases was observed: Cyclin-dependent kinases 2 (CDK2/Cyclin A), 5 (CDK5/p25) and 9 (CDK9/Cyclin T), proto-oncogene kinase PIM1, CDC2-like kinase 1 (CLK1), dual specificity tyrosine phosphorylation regulated kinase 1A (DYRK1A), glycogen-synthase kinase 3 (GSK3; α/β or β), casein kinase 1 (CK1; δ/ε or ε), and mitotic kinase Haspin). Table S1 in Supplementary Materials shows the results obtained.

## 3. Materials and Methods

### 3.1. General Information

All the reactions were performed under a dry argon atmosphere. THF was distilled over sodium/benzophenone. Column chromatography separations were achieved on silica gel (40–63 μm). Melting points were measured on a Kofler apparatus. IR spectra were taken on an ATR Spectrum 100 spectrometer (Perkin-Elmer). ^1^H- and ^13^C-Nuclear Magnetic Resonance (NMR) spectra were recorded either on an Avance III spectrometer (291 K) at 300 MHz and 75 MHz, respectively, or on an Avance III HD spectrometer (298 K) at 500 MHz and 126 MHz, respectively (Bruker, Billevica, Massachussets, USA). ^1^H chemical shifts (δ) are given in ppm relative to the solvent residual peak and ^13^C chemical shifts are relative to the central peak of the solvent signal [46]. 2,3-Diphenylpyrido[2,3-*b*]pyrazine (**2a**) [6], 8-bromo-2,3-diphenylpyrido[3,4-*b*]pyrazine (**3a**) [25,26] and 7-bromo-2,3-diphenylpyrido[2,3-*b*] pyrazine (**4a**) [6] were prepared as reported previously. The biological activity assays were performed as reported previously [44].

### 3.2. Crystallography

The X-ray diffraction data were collected either using an APEXII Bruker-AXS diffractometer (graphite monochromatized Mo-Kα radiation (λ = 0.71073 Å)) for the compounds **1b′** and **2i**, or using a D8 VENTURE Bruker AXS diffractometer (multilayer monochromatized Mo-Kα radiation (λ = 0.71073 Å)) equipped with a (CMOS) PHOTON 100 detector for **2f**, **2p**, **3h** and **2d**, at the temperature given in the crystal data. For **1b′** and **2i**, the structure was solved by direct methods using *SIR97* [47]. For **2f**, **2p**, **3h** and **2d**, they were solved by dual-space algorithm using the *SHELXT* program [48]. Structural refinements were performed with full-matrix least-square methods based on *F*^2^ (*SHELXL*) [49]. In the case of **2f** and **3h**, the contribution of the disordered solvents to the calculated structure factors was estimated following the *BYPASS* algorithm [50], implemented as the *SQUEEZE* option in *PLATON* [51]; a new data set, free of solvent contribution, was then used in the final refinement. All non-hydrogen atoms were refined with anisotropic atomic displacement parameters. Except nitrogen linked hydrogen atom that was introduced in the structural model through Fourier difference maps analysis (**2f**, **2p**, **3h**), H atoms were finally included in their calculated positions and treated as riding on their parent atom with constrained thermal parameters. The molecular diagrams were generated by ORTEP-3 (version 2.02) [52].

### 3.3. Deprotometalation Followed by Trapping with Electrophiles

#### 3.3.1. General Procedure 1

To a solution of 2,2,6,6-tetramethylpiperidine (0.51 mL, 3.0 mmol) in THF (3 mL) at 0 °C were successively added BuLi (about 1.6 M hexanes solution, 3.0 mmol) and, 15 min later, ZnCl_2_·TMEDA [53] (0.25 g, 1.0 mmol). After 15 min at 0 °C, the pyrazine (2.0 mmol) was introduced, and the mixture was stirred for 2 h at rt before addition of I_2_ (0.76 g, 3.0 mmol) in THF (3 mL) at 0 °C. The mixture was stirred at this temperature for 1 h before addition of an aqueous saturated solution of Na_2_S_2_O_3_ (10 mL) and extraction with EtOAc (3 × 20 mL). The combined organic layers were dried over MgSO_4_, filtered and concentrated under reduced pressure. The crude product was purified by chromatography over silica gel (the eluent is given in the product description).

#### 3.3.2. 5-Iodo-2,3-diphenylquinoxaline (**1b**)

The general procedure 1 using 2,3-diphenylquinoxaline (**1a**, 0.56 g) gave **1b** (eluent: heptane- CH_2_Cl_2_ 60:40; R_f_ = 0.55) in 74% yield as a pale yellow powder. Mp: 148 °C. IR: 486, 529, 551, 602, 689, 695, 701, 763, 776, 796, 892, 978, 1023, 1068, 1079, 1184, 1281, 1336, 1384, 1497, 1534, 3051 cm^−1^. ^1^H-NMR (CDCl_3_): 7.31–7.41 (m, 6H), 7.48 (dd, 1H, *J* = 8.3 and 7.4 Hz), 7.54–7.57 (m, 2H), 7.64–7.67 (m, 2H), 8.15 (dd, 1H, *J* = 8.4 and 1.3 Hz), 8.36 (dd, 1H, *J* = 7.4 and 1.3 Hz). ^13^C-NMR (CDCl_3_): 102.8 (C), 128.3 (2CH), 128.5 (2CH), 129.2 (CH), 129.3 (CH), 129.9 (2CH), 130.0 (CH), 130.5 (2CH), 131.0 (CH), 138.2 (C), 138.7 (C), 140.1 (CH), 140.9 (C), 141.3 (C), 153.9 (C), 154.1 (C). Anal. Calc. for C_20_H_13_IN_2_ (408.24): C 58.84, H 3.21, N, 6.86. Found: C 59.05, H 3.27, N, 6.70. 5,8-Diiodo-2,3-diphenylquinoxaline (**1b′**) was similarly isolated (eluent: heptane-CH_2_Cl_2_ 60:40; R_f_ = 0.69) in 7% yield as a yellow powder. Mp: 222 °C. IR: 533, 572, 613, 649, 692, 771, 824, 893, 978, 1025, 1055, 1077, 1169, 1209, 1325, 1383, 1447, 2930, 3059 cm^−1^. ^1^H-NMR (CDCl_3_): 7.34–7.45 (m, 6H), 7.64–7.76 (m, 4H), 8.02 (s, 2H). ^13^C-NMR (CDCl_3_): 103.5 (2C), 128.4 (4CH), 129.7 (2CH), 130.4 (4CH), 137.7 (2C), 140.6 (2CH), 140.8 (2C), 154.5 (2C). *Crystal data for* **1b′**. C_20_H_12_I_2_N_2_, *M* = 534.12, *T* = 150(2) K, monoclinic, *P* 2_1_, *a* = 10.1153(9), *b* = 5.8725(5), *c* = 14.9603(14) Å, β = 98.489(4) °, *V* = 878.94(14) Å^3^, *Z* = 2, *d* = 2.018 g cm^−3^, *μ* = 3.581 mm^−1^. A final refinement on *F*^2^ with 3888 unique intensities and 217 parameters converged at ω*R*(*F*^2^) = 0.0701 (*R*(*F*) = 0.0343) for 3602 observed reflections with *I* > 2σ(*I*). CCDC 1858478.

#### 3.3.3. 8-Iodo-2,3-diphenylpyrido[2,3-*b*]pyrazine (**2b-I**)

The general procedure 1 using 2,3-diphenylpyrido[2,3-*b*]pyrazine (**2a**, 0.57 g) gave **2b-I** (eluent: CH_2_Cl_2_; R_f_ = 0.34) in 70% yield as a whitish powder. Mp: 220 °C. IR: 534, 562, 613, 624, 637, 699, 980, 1023, 1336, 1416, 1519, 1570, 3068 cm^−1^. ^1^H-NMR (CDCl_3_): 7.32–7.44 (m, 6H), 7.64–7.69 (m, 4H), 8.28 (d, 1H, *J* = 4.5 Hz), 8.70 (d, 1H, *J* = 4.6 Hz). ^13^C-NMR (CDCl_3_): 116.1 (C), 128.3 (2CH), 128.4 (2CH), 129.7 (CH), 129.8 (CH), 130.3 (2CH), 130.3 (2CH), 135.6 (CH), 136.6 (C), 137.6 (C), 137.6 (C), 149.1 (C), 153.6 (CH), 155.0 (C), 157.1 (C). Anal. Calc. for C_19_H_12_IN_3_ (409.23): C 55.77, H 2.96, N, 10.27. Found: C 55.91, H 3.06, N, 10.03.

#### 3.3.4. 8-Bromo-2,3-diphenylpyrido[2,3-*b*]pyrazine (**2b-Br**)

To a stirred mixture of 2,3-diphenyl pyrido[2,3-*b*]pyrazine (**2a**, 0.28 g, 1.0 mmol) and ZnCl_2_· TMEDA [53] (0.26 g, 1.0 mmol) in THF (1 mL) at −20 °C was added dropwise a solution of LiTMP (prepared by adding BuLi (about 1.6 M hexanes solution, 1.2 mmol) to a stirred, cooled (−20 °C) solution of 2,2,6,6-tetramethylpiperidine (0.24 mL, 1.2 mmol) in THF (2 mL) and stirring for 15 min) cooled at −20 °C. After 30 min at −20 °C, Br_2_ (97 μL, 2.0 mmol) was introduced, and the mixture was stirred for 1 h before addition of an aqueous saturated solution of Na_2_S_2_O_3_ (5 mL) and extraction with EtOAc (3 × 20 mL). The combined organic layers were dried over MgSO_4_, filtered and concentrated under reduced pressure. The crude product was purified by chromatography over silica gel (eluent: CH_2_Cl_2_-EtOAc 90:10; R_f_ = 0.50) to give **2b-Br** in 60% yield as a whitish powder. Mp: 183 °C. IR: 491, 538, 563, 615, 625, 649, 698, 767, 839, 985, 1021, 1049, 1090, 1179, 1241, 1336, 1387, 1421, 1460, 1524, 1584, 3067 cm^−1^. ^1^H-NMR (CDCl_3_): 7.32–7.42 (m, 6H), 7.63–7.66 (m, 4H), 8.00 (d, 1H, *J* = 4.7 Hz), 8.91 (d, 1H, *J* = 4.7 Hz). ^13^C-NMR (CDCl_3_): 128.3 (CH), 128.4 (CH), 128.7 (CH), 129.7 (CH), 129.8 (CH), 130.3 (CH), 130.3 (CH), 134.7 (C), 136.3 (C), 137.7 (C), 137.9 (C), 150.1 (C), 153.4 (CH), 154.9 (C), 157.0 (C). Anal. Calc. for C_19_H_12_BrN_3_ (362.23): C 63.00, H 3.34, N, 11.60. Found: C 63.24, H 3.58, N, 11.43.

#### 3.3.5. 8-Chloro-2,3-diphenylpyrido[2,3-*b*]pyrazine (**2b-Cl**)

To a stirred mixture of 2,3-diphenyl pyrido[2,3-*b*]pyrazine (**2a**, 0.28 g, 1.0 mmol) and ZnCl_2_· TMEDA [53] (0.26 g, 1.0 mmol) in THF (1 mL) at −20 °C was added dropwise a solution of LiTMP (prepared by adding BuLi (about 1.6 M hexanes solution, 1.2 mmol) to a stirred, cooled (−20 °C) solution of 2,2,6,6-tetramethylpiperidine (0.24 mL, 1.2 mmol) in THF (2 mL) and stirring for 15 min) cooled at −20 °C. After 30 min at −20 °C, trichloroisocyanuric acid (0.30 g, 1.3 mmol) was introduced (CAUTION: dissolution of trichloroisocyanuric acid in THF at a temperature above 0 °C produces intense heat), and the mixture was stirred at this temperature for 1 h before addition of water (5 mL) and extraction with EtOAc (3 × 20 mL). The combined organic layers were dried over MgSO_4_, filtered and concentrated under reduced pressure. The crude product was purified by chromatography over silica gel (eluent: CH_2_Cl_2_-EtOAc 90:10; R_f_ = 0.60) to give **2b-Cl** in 62% yield as a whitish powder. Mp: 180 °C. IR: 534, 544, 617, 658, 699, 770, 851, 991, 1025, 1055, 1193, 1242, 1341, 1388, 1422, 1442, 1452, 1532, 1583, 3034, 3051 cm^−1^. ^1^H-NMR (CDCl_3_): 7.31–7.44 (m, 6H), 7.62–7.66 (m, 4H), 7.79 (d, 1H, *J* = 4.7 Hz), 9.02 (d, 1H, *J* = 4.7 Hz). ^13^C-NMR (CDCl_3_): 125.1 (CH), 128.3 (CH), 128.5 (CH), 129.7 (CH), 129.8 (CH), 130.2 (CH), 130.3 (CH), 133.7 (C), 137.8 (C), 138.1 (C), 144.5 (C), 150.5 (C), 153.3 (CH), 154.8 (C), 157.1 (C). Anal. Calc. for C_19_H_12_ClN_3_ (317.78): C 71.81, H 3.81, N, 13.22. Found: C 71.77, H 3.85, N, 13.14.

#### 3.3.6. General Procedure 2

To a stirred mixture of the pyrazine (1.0 mmol) and ZnCl_2_·TMEDA [53] (0.26 g, 1.0 mmol) in THF (1 mL) at −20 °C was added dropwise a solution of LiTMP (prepared by adding BuLi (about 1.6 M hexanes solution, 1.2 mmol) to a stirred, cooled (−20 °C) solution of 2,2,6,6-tetramethylpiperidine (0.24 mL, 1.2 mmol) in THF (2 mL) and stirring for 15 min) cooled at −20 °C. After 30 min at −20 °C, I_2_ (0.37 g, 1.5 mmol) in THF (2 mL) was introduced, and the mixture was stirred at this temperature for 1 h before addition of an aqueous saturated solution of Na_2_S_2_O_3_ (5 mL) and extraction with EtOAc (3 × 20 mL). The combined organic layers were dried over MgSO_4_, filtered and concentrated under reduced pressure. The crude product was purified by chromatography over silica gel (the eluent is given in the product description).

#### 3.3.7. 8-Bromo-7-iodo-2,3-diphenylpyrido[3,4-*b*]pyrazine (**3b**)

The general procedure 2 using 8-bromo-2,3-diphenylpyrido[3,4-*b*]pyrazine (**3a** [25,26], 0.36 g) gave **3b** (eluent: CH_2_Cl_2_-petroleum ether 80:20; R_f_ = 0.44) in 67% yield as a red powder. Mp: 186–188 °C. IR: 493, 529, 559, 600, 658, 695, 765, 984, 1025, 1055, 1117, 1238, 1315, 1373, 1399, 1446, 1493, 1551, 3034, 3060 cm^−1^. ^1^H-NMR (CDCl_3_): 7.34–7.47 (m, 6H), 7.54–7.57 (m, 2H), 7.62–7.65 (m, 2H), 9.27 (s, 1H). ^13^C-NMR (CDCl_3_): 121.7 (C), 128.6 (2CH), 128.7 (2CH), 129.7 (C), 129.8 (2CH), 130.1 (CH), 130.5 (2CH), 130.5 (CH), 136.0 (C), 137.4 (C), 137.7 (C), 142.3 (C), 152.5 (CH), 156.2 (C), 158.6 (C). Anal. Calc. for C_19_H_11_BrIN_3_ (488.13): C 46.75, H 2.27, N, 8.61. Found: C 46.89, H 2.49, N, 8.55. 8-Bromo-5,7-diiodo-2,3-diphenyl pyrido[3,4-*b*]pyrazine, also formed in <5% yield, was identified by its ^1^H-NMR (CDCl_3_): 7.36–7.47 (m, 6H), 7.54–7.57 (m, 2H), 7.65–7.68 (m, 4H).

#### 3.3.8. 7-Bromo-6-iodo-2,3-diphenylpyrido[2,3-*b*]pyrazine (**4b**)

The general procedure 2 using 7-bromo-2,3-diphenylpyrido[2,3-*b*]pyrazine (**4a** [54], prepared in 90% yield [6], 0.36 g) gave **4b** (eluent: CH_2_Cl_2_-heptane 70:30; R_f_ (heptane-CH_2_Cl_2_ 80:20) = 0.80) in 5% yield as a yellow powder. Mp: 150–152 °C. IR: 495, 547, 596, 615, 697, 731, 770, 778, 903, 937, 1025, 1060, 1107, 1178, 1274, 1332, 1390, 1448, 1502, 1562, 1603, 1699, 1768, 2734, 2940, 3064 cm^−1^. ^1^H-NMR (CDCl_3_): 7.30–7.43 (m, 6H), 7.52–7.55 (m, 2H), 7.59–7.62 (m, 2H), 8.62 (s, 1H). ^13^C-NMR (CDCl_3_): 128.3 (2CH), 128.6 (2CH), 128.7 (C), 129.9 (CH), 129.9 (2CH), 130.0 (CH), 130.0 (C), 130.3 (2CH), 135.5 (C), 137.6 (C), 138.0 (C), 139.5 (CH), 148.0 (C), 155.9 (C), 157.1 (C). Anal. Calc. for C_19_H_11_BrIN_3_ (488.13): C 46.75, H 2.27, N, 8.61. Found: C 46.93, H 2.38, N, 8.49.

### 3.4. Suzuki Coupling Reactions

#### 3.4.1. General Procedure 3

To a stirred mixture of the iodide (0.50 mmol) and Pd(PPh_3_)_4_ (29 mg, 25 μmol) in degassed 1,2-dimethoxyethane (5 mL) was added the boronic acid (0.60 mmol) and NaHCO_3_ (2.0 mmol) in degassed water (1.6 mL). The resulting mixture was heated at 80 °C for 3 h and cooled to rt before addition of water (5 mL) and extraction with EtOAc (3 × 10 mL). The combined organic layers were washed with brine (10 mL), dried over MgSO_4_, filtered and concentrated under reduced pressure. The crude product was purified by chromatography over silica gel (the eluent is given in the product description).

#### 3.4.2. 2,3,5-Triphenylquinoxaline (**1c**)

The general procedure 3 using 5-iodo-2,3-diphenyl quinoxaline **(1b**, 0.20 g) and phenylboronic acid (73 mg) gave **1c** (eluent: CH_2_Cl_2_-heptane 60:40; R_f_ = 0.35) in 42% yield as a white powder. Mp: 150 °C. IR: 763, 804, 841, 927, 984, 1023, 1081, 1128, 1233, 1336, 1388, 1433, 1444, 1491, 1566, 2858, 2927, 2965, 3064 cm^−1^. ^1^H-NMR (CDCl_3_): 7.26–7.34 (m, 3H), 7.36–7.48 (m, 4H), 7.52–7.64 (m, 6H), 7.81–7.89 (m, 4H), 8.21 (dd, 1H, *J* = 7.3 and 2.5 Hz). ^13^C-NMR (CDCl_3_): 127.7 (CH), 128.0 (2CH), 128.1 (2CH), 128.5 (2CH), 128.7 (CH), 128.8 (CH), 129.0 (CH), 129.8 (CH), 129.9 (2CH), 130.3 (2CH), 130.4 (CH), 131.1 (2CH), 138.4 (C), 139.0 (C), 139.1 (C), 139.4 (C), 140.6 (C), 141.3 (C), 152.4 (C), 152.9 (C). Anal. Calc. for C_26_H_18_N_2_ (358.44): C 87.12, H 5.06, N, 7.82. Found: C 87.25, H 5.22, N, 7.70.

#### 3.4.3. 2,3-Diphenyl-5-(2-thienyl)quinoxaline (**1d**)

The general procedure 3 using 5-iodo-2,3-diphenylquinoxaline (**1b**, 0.20 g) and 2-thienylboronic acid (77 mg) gave **1d** (eluent: CH_2_Cl_2_-heptane 60:40; R_f_ = 0.20) in 97% yield as a yellow powder. Mp: 210 °C. IR: 738, 766, 796, 828, 854, 916, 933, 969, 1025, 1053, 1083, 1163, 1238, 1336, 1390, 1442, 1495, 1562, 1592, 3064 cm^−1^. ^1^H-NMR (CDCl_3_): 7.18 (dd, 1H, *J* = 5.1 and 3.7 Hz), 7.32–7.40 (m, 6H), 7.51 (dd, 1H, *J* = 5.1 and 1.2 Hz), 7.58–7.61 (m, 2H), 7.67–7.70 (m, 2H), 7.76 (dd, 1H, *J* = 8.3 and 7.4 Hz), 7.88 (dd, 1H, *J* = 3.7 and 1.2 Hz), 8.08 (dd, 1H, *J* = 8.3 and 1.3 Hz), 8.13 (dd, 1H, *J* = 7.4 and 1.3 Hz). ^13^C-NMR (CDCl_3_): 126.7 (CH), 126.9 (CH), 127.5 (CH), 128.1 (CH), 128.2 (2CH), 128.5 (2CH), 128.8 (CH), 129.0 (CH), 129.0 (CH), 129.9 (2CH), 129.9 (CH), 130.6 (2CH), 133.0 (C), 137.6 (C), 138.8 (C), 138.9 (C), 139.2 (C), 141.4 (C), 152.3 (C), 153.2 (C). Anal. Calc. for C_24_H_16_N_2_S (364.47): C 79.09, H 4.43, N, 7.69. Found: C 79.11, H 4.48, N, 7.72.

#### 3.4.4. 2,3-Diphenyl-8-(2-thienyl)pyrido[2,3-*b*]pyrazine (**2d**)

The general procedure 3 using 8-iodo-2,3-diphenylpyrido[2,3-*b*]pyrazine (**2b-I**, 0.20 g) and 2-thienylboronic acid (77 mg) gave **2d** (eluent: CH_2_Cl_2_-EtOAc 95:5; R_f_ = 0.50) in 75% yield as a pale yellow powder. Mp: 215 °C. IR: 540, 695, 744, 1025, 1096, 1120, 1188, 1238, 1336, 1384, 1435, 1480, 551, 1568, 2927, 2965, 3060 cm^−1^. ^1^H-NMR (CDCl_3_): 7.23 (dd, 1H, *J* = 5.1 and 3.8 Hz), 7.32–7.45 (m, 6H), 7.66–7.72 (m, 5H), 7.98 (d, 1H, *J* = 4.8 Hz), 8.10 (dd, 1H, *J* = 3.8 and 1.2 Hz), 9.10 (d, 1H, *J* = 4.8 Hz). ^13^C-NMR (CDCl_3_): 120.3 (CH), 127.2 (CH), 128.3 (2CH), 128.4 (2CH), 129.2 (CH), 129.4 (CH), 129.5 (CH), 130.3 (2CH), 130.5 (2CH), 132.4 (CH), 132.4 (C), 136.0 (C), 138.2 (C), 138.3 (C), 140.9 (C), 150.2 (C), 153.1 (C), 153.9 (CH), 155.8 (C). *Crystal data for* **2d.** C_23_H_15_N_3_S, *M* = 365.44, *T* = 150(2) K, triclinic, *P* 1, *a* = 6.6311(18), *b* = 9.939(3), *c* = 13.655(4) Å, α = 81.914(12), β = 80.405(11), γ = 89.955(10) °, *V* = 878.3(4) Å^3^, *Z* = 2, *d* = 1.382 g cm^−3^, *μ* = 0.197 mm^−1^. A final refinement on *F*^2^ with 7113 unique intensities and 236 parameters converged at ω*R*(*F*^2^) = 0.3351 (*R*(*F*) = 0.1327) for 6147 observed reflections with *I* > 2σ(*I*). CCDC 1858479.

#### 3.4.5. 5-(2-Aminophenyl)-2,3-diphenylquinoxaline (**1e**)

The general procedure 3 using 5-iodo-2,3-diphenylquinoxaline **(1b**, 0.20 g) and 2-amino- phenylboronic acid (82 mg) gave **1e** (eluent: heptane-CH_2_Cl_2_ 70:30; R_f_ = 0.31) in 92% yield as a yellow powder. Mp: 178 °C. IR: 689, 702, 740, 771, 977, 1307, 1342, 1492, 1626, 3025, 3060, 3212, 3328, 3468 cm^−1^. ^1^H-NMR (CDCl_3_): 3.87 (br s, 2H, NH_2_), 6.85 (dd, 1H, *J* = 7.9 and 1.1 Hz), 6.92 (td, 1H, *J* = 7.4 and 1.2 Hz), 7.21–7.30 (m, 5H), 7.35–7.40 (m, 3H), 7.47–7.50 (m, 2H), 7.55–7.58 (m, 2H), 7.78–7.86 (m, 2H), 8.20 (dd, 1H, *J* = 7.8 and 2.1 Hz). ^13^C-NMR (CDCl_3_): 116.5 (CH), 118.8 (CH), 125.7 (C), 128.1 (2CH), 128.5 (2CH), 128.9 (CH), 129.0 (CH), 129.0 (CH), 129.0 (CH), 129.9 (2CH), 130.2 (CH), 130.3 (2CH), 132.0 (CH), 132.3 (CH), 138.8 (C), 139.2 (C), 139.3 (C), 139.6 (C), 141.3 (C), 145.0 (C), 152.5 (C), 153.2 (C). Anal. Calc. for C_26_H_19_N_3_ (373.46): C 83.62, H 5.13, N, 11.25. Found: C 83.81, H 5.26, N, 11.17.

#### 3.4.6. 8-(2-Aminophenyl)-2,3-diphenylpyrido[2,3-*b*]pyrazine (**2e**)

The general procedure 3 using 8-iodo-2,3-diphenylpyrido[2,3-*b*]pyrazine **(2b-I**, 0.20 g) and 2-aminophenylboronic acid (82 mg) gave **2e** (eluent: CH_2_Cl_2_-EtOAc 70:30; R_f_ = 0.50) in 73% yield as a yellow powder. Mp: 205 °C. IR: 687, 742, 766, 854, 981, 1015, 1047, 1237, 1307, 1382, 1489, 1623, 3024, 3055, 3345 cm^−1^. ^1^H-NMR (CDCl_3_): 3.99 (br s, 2H, NH_2_), 6.87 (dd, 1H, *J* = 8.4 and 1.2 Hz), 6.94 (td, 1H, *J* = 7.4 and 1.2 Hz), 7.25–7.40 (m, 8H), 7.51–7.54 (m, 2H), 7.65–7.68 (m, 2H), 7.73 (d, 1H, *J* = 4.5 Hz), 9.19 (d, 1H, *J* = 4.4 Hz). ^13^C-NMR (CDCl_3_): 116.9 (CH), 118.7 (CH), 122.6 (C), 126.3 (CH), 128.2 (2CH), 128.2 (2CH), 129.3 (CH), 129.5 (CH), 130.1 (CH), 130.1 (2CH), 130.1 (2CH), 132.0 (CH), 134.3 (C), 138.1 (C), 138.2 (C), 144.9 (C), 149.0 (C), 149.8 (C), 153.4 (C), 154.1 (CH), 155.7 (C). Anal. Calc. for C_25_H_18_N_4_ (374.45): C 80.19, H 4.85, N, 14.96. Found: C 80.07, H 4.87, N, 14.85.

#### 3.4.7. 2,3-Diphenyl-11*H*-pyrazino[2′,3′:4,5]pyrido[2,3-*d*]indole (**3h**)

In a tube containing a stirred mixture of 8-bromo-7-iodo-2,3-diphenylpyrido[3,4-*b*]pyrazine (**3b**, 0.24 g, 0.50 mmol) and Pd(PPh_3_)_4_ (29 mg, 25 μmol) in degassed 1,2-dimethoxyethane (5 mL) was introduced 2-aminophenylboronic acid (82 mg, 0.60 mmol) and Na_2_CO_3_ (2.0 mmol) in degassed water (1.6 mL). The sealed tube was heated overnight at 140 °C and cooled to rt before addition of saturated aqueous NaHCO_3_ (5 mL) and extraction with EtOAc (3 × 10 mL). The combined organic layers were washed with brine (10 mL), dried over MgSO_4_, filtered and concentrated under reduced pressure. The crude product was purified by chromatography over silica gel (eluent: CH_2_Cl_2_-EtOAc 90:10; R_f_ = 0.28) to give **3h** in 65% yield as a yellow powder. Mp: 284–286 °C. IR: 695, 748, 763, 1025, 1092, 1190, 1236, 1315, 1328, 1336, 1376, 1446, 1495, 1540, 1624, 3034, 3064, 3420 cm^−1^. ^1^H-NMR (CDCl_3_): 7.30–7.42 (m, 7H), 7.50–7.60 (m, 6H), 8.45 (d, 1H, *J* = 7.9 Hz), 9.47 (s, 1H), 9.78 (br s, 1H). ^13^C-NMR (CDCl_3_): 111.9 (CH), 120.6 (CH), 121.3 (CH), 123.1 (C), 126.7 (C), 127.2 (CH), 128.4 (2CH), 128.5 (2CH), 129.2 (CH), 129.6 (CH), 130.0 (2CH), 130.1 (2CH), 132.4 (C), 134.9 (C), 138.4 (C), 138.6 (C), 138.8 (C), 139.5 (C), 146.5 (CH), 153.6 (C), 155.9 (C). *Crystal data for* **3h.** C_25_H_16_N_4_, *M* = 372.42, *T* = 150(2) K, orthorhombic, *Pbca*, *a* = 7.1524(9), *b* = 16.3313(17), *c* = 33.798(4) Å, *V* = 3947.9(8) Å^3^, *Z* = 8, *d* = 1.253 g cm^−3^, *μ* = 0.076 mm^−1^. A final refinement on *F*^2^ with 4429 unique intensities and 265 parameters converged at ω*R*(*F*^2^) = 0.1564 (*R*(*F*) = 0.0739) for 3511 observed reflections with *I* > 2σ(*I*). CCDC 1858477. This compound was also obtained in 64% yield under microwave irradiation (300 W; Monowave 300, Anton Paar, Graz, Austria) for 30 min at 150 °C.

### 3.5. 8-(2-Azidophenyl)-2,3-diphenylpyrido[2,3-b]pyrazine

To a stirred solution of 8-(2-aminophenyl)-2,3-diphenylpyrido[2,3-*b*]pyrazine (**2e**, 94 mg, 0.25 mmol) in acetic acid (1.5 mL) at 0 °C was added 1M aqueous NaNO_2_ (0.35 mL, 0.35 mmol). After stirring for 1 h at rt, the solution was cooled to 0 °C before addition of 1M aqueous NaN_3_ (0.35 mL, 0.35 mmol). After stirring overnight at rt, 3 mL of saturated aqueous NaHCO_3_ were added. Extraction with EtOAc (3 × 10 mL), washing of the combined organic layers with brine (10 mL), drying over MgSO_4_, filtration and concentration under reduced pressure afforded a brown powder which was purified by chromatography over silica gel (eluent: CH_2_Cl_2_-EtOAc 95:5; R_f_ = 0.50) to afford the azide in 64% yield. IR: 685, 745, 1288, 1440, 1577, 2088, 2124, 3064 cm^−1^. ^1^H-NMR (CDCl_3_): 7.23–7.40 (m, 8H), 7.45–7.57 (m, 4H), 7.66–7.69 (m, 3H), 9.18 (d, 1H, *J* = 4.4 Hz). ^13^C-NMR (CDCl_3_): 118.8 (CH), 124.7 (CH), 126.0 (CH), 127.9 (C), 128.2 (2CH), 128.2 (2CH), 129.2 (CH), 129.5 (CH), 130.1 (2CH), 130.3 (2CH), 130.4 (CH), 132.5 (CH), 134.5 (C), 138.3 (C), 138.5 (C), 138.7 (C), 146.7 (C), 149.8 (C), 153.5 (CH), 153.7 (C), 155.8 (C).

### 3.6. Palladium-Catalyzed N-arylation

#### 3.6.1. General Procedure 4 

To a stirred mixture of the halide (0.50 mmol) and Cs_2_CO_3_ (0.48 g, 1.5 mmol) in 2-chloroaniline (63 μL, 0.60 mmol) was added a solution of the catalyst prepared by stirring Pd_2_(dba)_3_ (11 mg, 12.5 μmol) and Xantphos (16 mg, 27.5 μmol) in degassed dioxane (2 mL) for 10 min at rt. The resulting mixture was heated at 110 °C for 24 h and cooled to rt before addition of water (5 mL) and extraction with EtOAc (3 × 10 mL). The combined organic layers were washed with brine (10 mL), dried over MgSO_4_, filtered and concentrated under reduced pressure. The crude product was purified by chromatography over silica gel (the eluent is given in the product description).

#### 3.6.2. 5-(2-Chlorophenylamino)-2,3-diphenylquinoxaline (**1f**)

The general procedure 4 using 5-iodo-2,3-diphenylquinoxaline (**1b**, 0.20 g) gave **1f** (eluent: heptane-CH_2_Cl_2_ 60:40; R_f_ = 0.42) in 92% yield as a yellow powder. Mp: 182 °C. IR: 695, 729, 748, 959, 1021, 1055, 1072, 1098, 1182, 1218, 1317, 1343, 1356, 1394, 1442, 1454, 1497, 1534, 1562, 1579, 1594, 1613, 3060, 3347 cm^−1^. ^1^H-NMR (CDCl_3_): 6.95 (td, 1H, *J* = 7.7 and 1.4 Hz), 7.27–7.39 (m, 7H), 7.46–7.52 (m, 2H), 7.55–7.62 (m, 4H), 7.64–7.66 (m, 2H), 7.71 (dd, 1H, *J* = 8.2 and 1.4 Hz), 8.56 (br s, 1H). ^13^C-NMR (CDCl_3_): 109.1 (CH), 118.9 (CH), 118.9 (CH), 122.6 (CH), 125.1 (C), 127.6 (CH), 128.2 (2CH), 128.4 (2CH), 128.9 (CH), 128.9 (CH), 129.9 (2CH), 130.1 (2CH), 130.2 (CH), 130.9 (CH), 132.1 (C), 138.4 (C), 138.9 (C), 139.2 (C), 139.3 (C), 141.9 (C), 150.4 (C), 153.9 (C). Anal. Calc. for C_26_H_18_ClN_3_ (407.90): C 76.56, H 4.45, N, 10.30. Found: C 76.89, H 4.58, N, 10.13.

#### 3.6.3. 8-(2-Chlorophenylamino)-2,3-diphenylpyrido[2,3-*b*]pyrazine (**2f**)

The general procedure 4 using 8-iodo-2,3-diphenylpyrido[2,3-*b*]pyrazine (**2b-I**, 0.20 g) gave **2f** (eluent: CH_2_Cl_2_-EtOAc 90:10; R_f_ = 0.32) in 67% yield as a yellow powder. Mp: 202 °C. IR: 542, 699, 755, 1021, 1102, 1242, 1313, 1336, 1356, 1437, 1452, 1534, 1558, 1583, 1646, 3060, 3322, 3631 cm^−1^. ^1^H-NMR (CDCl_3_): 7.13–7.19 (m, 2H), 7.30–7.41 (m, 7H), 7.53 (dd, 1H, *J* = 8.0 and 1.5 Hz), 7.57–7.65 (m, 4H), 7.68 (dd, 1H, *J* = 8.1 and 1.5 Hz), 8.77 (br s, 1H, NH), 8.81 (d, 1H, *J* = 5.4 Hz, H_6_). ^13^C-NMR (CDCl_3_): 102.8 (CH), 122.2 (CH), 125.5 (CH), 127.2 (C), 127.5 (C), 127.8 (CH), 128.2 (2CH), 128.4 (2CH), 129.2 (CH), 129.4 (CH), 130.0 (2CH), 130.3 (2CH), 130.5 (CH), 136.2 (C), 138.4 (C), 138.5 (C), 147.0 (C), 150.3 (C), 151.1 (C), 155.0 (CH), 156.5 (C). *Crystal data for* **2f.** C_25_H_17_ClN_4_, *M* = 408.88, *T* = 150(2) K, orthorhombic, *P c a* 2_1_, *a* = 15.3485(15), *b* = 18.8937(16), *c* = 6.9936(7) Å, *V* = 2028.1(3) Å^3^, *Z* = 4, *d* = 1.339 g cm^−3^, *μ* = 0.208 mm^−1^. A final refinement on *F*^2^ with 4578 unique intensities and 274 parameters converged at ω*R*(*F*^2^) = 0.1478 (*R*(*F*) = 0.0583) for 4133 observed reflections with *I* > 2σ(*I*). CCDC 1858474.

### 3.7. Palladium-Catalyzed N-arylation

2,3-Diphenyl-11*H*-pyrazino[2,3-*a*]carbazole (**1g**) was prepared by adapting a reported procedure [40]. To a stirred mixture of 5-(2-chlorophenylamino)-2,3-diphenylquinoxaline (**1f**, 0.24 g, 0.60 mmol) and 1,8-diazabicyclo[5.4.0]undec-7-ene (0.13 mL, 0.90 mmol), was added a solution of the catalyst prepared by stirring Pd_2_(dba)_3_ (14 mg, 15 μmol) and P(*t*Bu)_3_ (12 mg, 60 μmol) in degassed dioxane (1 mL) for 10 min at rt. The resulting mixture was heated by microwave irradiation (300 W; Monowave 300, Anton Paar, Graz, Austria) for 10 min at 180 °C before addition of water (5 mL) and extraction with EtOAc (3 × 10 mL). The combined organic layers were washed with brine (10 mL), dried over MgSO_4_, filtered and concentrated under reduced pressure. The crude product was purified by chromatography over silica gel (eluent: heptane-CH_2_Cl_2_ 60:40; R_f_ = 0.48) to give **1g** in 62% yield as a yellow powder. Mp: 260 °C. IR: 1025, 1087, 1102, 1175, 1242, 1326, 1347, 1362, 1384, 1444, 1459, 1624, 1731, 2854, 2922, 3420 cm^−1^. ^1^H-NMR ((CD_3_)_2_SO): 6.86 (ddd, 1H, *J* = 8.0, 7.1 and 1.0 Hz), 6.91–6.97 (m, 6H), 7.01–7.10 (m, 3H), 7.14–7.17 (m, 2H), 7.28 (d, 1H, *J* = 8.3 Hz), 7.39 (d, 1H, *J* = 8.7 Hz), 7.84 (d, 1H, *J* = 7.8 Hz), 8.14 (d, 1H, *J* = 8.7 Hz), 12.13 (br s, 1H). ^13^C-NMR ((CD_3_)_2_SO): 112.2 (CH), 118.8 (CH), 119.7 (CH), 120.3 (CH), 120.7 (C), 122.7 (C), 124.2 (CH), 125.6 (CH), 128.0 (2CH), 128.0 (2CH), 128.5 (CH), 128.6 (CH), 129.7 (2CH), 129.9 (2CH), 130.0 (C), 134.3 (C), 139.0 (C), 139.2 (C), 139.8 (C), 139.9 (C), 150.7 (C), 151.4 (C). Anal. Calc. for C_26_H_17_N_3_ (371.44): C 84.07, H 4.61, N, 11.31. Found: C 84.19, H 4.52, N, 11.12.

### 3.8. One-Pot Palladium-Catalyzed N-arylation/C-H Arylation

#### 3.8.1. General Procedure 5

To a mixture of the halide (0.25 mmol), 1,8-diazabicyclo[5.4.0]undec-7-ene (118 μL, 0.75 mmol), 2-chloroaniline (38 mg, 0.30 mmol), Pd_2_(dba)_3_ (9.2 mg, 10 μmol) and Xantphos (13 mg, 22 μmol), was added degassed 1,4-dioxane (1 mL). The mixture was heated by microwave irradiation (150 W; Monowave 300, Anton Paar, Graz, Austria) under the conditions given in the product description. The cooled residue was taken up with EtOAc (20 mL). The organic layer was washed with brine (10 mL), dried over MgSO_4_, filtered and concentrated under reduced pressure. The crude product was purified by chromatography over silica gel (the eluent is given in the product description).

#### 3.8.2. 2,3-Diphenyl-11*H*-pyrazino[2′,3′:5,6]pyrido[4,3-*b*]indole (**2g**)

The general procedure 5 (1 h at 180 °C) using 8-iodo-2,3-diphenylpyrido[2,3-*b*]pyrazine (**2b-I**, 0.10 g) gave **2g** (eluent: CH_2_Cl_2_-EtOAc 90:10; R_f_ = 0.43) in 70% yield as a white powder. Mp > 260 °C. IR: 525, 542, 551, 626, 699, 750, 768, 1025, 1045, 1075, 1100, 1236, 1339, 1373, 1444, 1555, 1736, 2665, 3056 cm^−1^. ^1^H-NMR ((CD_3_)_2_SO): 7.40–7.45 (m, 7H), 7.55–7.63 (m, 5H), 7.77 (dd, 1H, *J* = 8.2 and 0.9 Hz), 8.42 (dt, 1H, *J* = 7.8 and 1.0 Hz), 9.87 (s, 1H), 13.19 (s, 1H). ^13^C-NMR ((CD_3_)_2_SO): 112.6 (CH), 118.2 (C), 120.7 (CH), 121.3 (CH), 121.4 (C), 126.4 (C), 126.5 (CH), 128.0 (CH), 128.0 (2CH), 128.1 (2CH), 128.8 (CH), 129.8 (2CH), 129.9 (2CH), 138.6 (C), 138.7 (C), 139.4 (C), 140.0 (C), 147.4 (C), 148.5 (CH), 151.4 (C), 153.4 (C). Anal. Calc. for C_25_H_16_N_4_ (372.43): C 80.63, H 4.33, N, 15.04. Found: C 80.54, H 4.28, N, 14.89.

#### 3.8.3. 7-(Phenylamino)-2,3-diphenylpyrido[3,4-*b*]pyrazine (**3g′**)

The general procedure 5 (40 min at 180 °C) using 8-bromo-7-iodo-2,3-diphenylpyrido[3,4-*b*] pyrazine (**3b**, 0.12 g) gave **3g′** (eluent: CH_2_Cl_2_-MeOH 99:1; R_f_ = 0.27) in 32% yield as a yellow powder. Mp: 224–226 °C. IR: 699, 750, 770, 978, 1025, 1057, 1077, 1169, 1197, 1261, 1336, 1349, 1435, 1450, 1527, 1555, 1588, 1613, 2854, 2927, 2961, 3025, 3232 cm^−1^. ^1^H-NMR (CDCl_3_): 7.14 (p, 1H, *J* = 4.4 Hz), 7.23 (br s, 1H), 7.29–7.49 (m, 15H), 9.26 (s, 1H). ^13^C-NMR (CDCl_3_): 158.3 (C), 155.8 (C), 154.0 (CH), 151.4 (C), 146.3 (C), 139.8 (C), 138.8 (C), 138.7 (C), 132.4 (C), 129.8 (2CH), 129.7 (2CH), 129.7 (2CH), 129.5 (CH), 128.9 (CH), 128.4 (2CH), 128.4 (2CH), 124.1 (CH), 121.4 (2CH), 98.3 (CH). Anal. Calc. for C_25_H_18_N_4_ (374.45): C 80.19, H 4.85, N, 14.96. Found: C 80.17, H 4.99, N, 14.84.

### 3.9. Copper-Catalyzed N-arylation

#### 3.9.1. General Procedure 6

A mixture containing the iodide (0.50 mmol) and azole (1.0 mmol), Cu_2_O (6.0 mg, 0.10 mmol), Cs_2_CO_3_ (0.33 g, 1.0 mmol) and DMSO (0.5 mL) was stirred at 110 °C for 24 h. The cooled residue was taken up with EtOAc (20 mL) and filtered through a Celite pad. The organic layer was washed with water (10 mL) and brine (10 mL), dried over MgSO_4_, filtered and concentrated under reduced pressure. The crude product was purified by chromatography over silica gel (the eluent is given in the product description).

#### 3.9.2. 2,3-Diphenyl-8-(*N*-pyrrolyl)pyrido[2,3-*b*]pyrazine (**2i**)

The general procedure 6 using 8-iodo-2,3-diphenylpyrido[2,3-*b*]pyrazine (**2b-I**, 0.20 g) and pyrrole (67 mg) gave **2i** (eluent: CH_2_Cl_2_-EtOAc 90:10; R_f_ = 0.47) in 67% yield as a yellow powder. Mp: 210 °C. IR: 946, 1025, 1072, 1096, 1107, 1173, 1238, 1289, 1328, 1362, 1388, 1433, 1454, 1482, 1549, 1588, 3025, 3060, 3111, 3141, 3180 cm^−1^. ^1^H-NMR (CDCl_3_): 6.39–6.40 (m, 2H), 7.24–7.35 (m, 6H), 7.49–7.53 (m, 3H), 7.59–7.62 (m, 2H), 7.65–7.66 (m, 2H), 9.01 (d, 1H, *J* = 5.0 Hz). ^13^C-NMR (CDCl_3_): 112.0 (2CH), 115.1 (CH), 122.7 (2CH), 128.3 (2CH), 128.4 (2CH), 129.4 (C), 129.5 (CH), 129.8 (CH), 130.0 (2CH), 130.2 (2CH), 137.8 (C), 138.1 (C), 144.6 (C), 150.7 (C), 153.2 (C), 153.9 (CH), 156.0 (C). *Crystal data for* **2i.** C_23_H_16_N_4_, *M* = 348.40, *T* = 150(2) K, orthorhombic, *P 2_1_ 2_1_ 2_1_*, *a* = 6.3672(5), *b* = 13.0997(10), *c* = 21.5377(18) Å, *V* = 1796.4(2) Å^3^, *Z* = 4, *d* = 1.288 g cm^−3^, *μ* = 0.079 mm^−1^. A final refinement on *F*^2^ with 2367 unique intensities and 245 parameters converged at ω*R*(*F*^2^) = 0.1207 (*R*(*F*) = 0.0498) for 1679 observed reflections with *I* > 2σ(*I*). CCDC 1858475.

#### 3.9.3. 8-(*N*-indolyl)-2,3-diphenylpyrido[2,3-*b*]pyrazine (**2j**)

The general procedure 6 using 8-iodo-2,3-diphenylpyrido[2,3-*b*]pyrazine (**2b-I**, 0.20 g) and indole (0.12 g) gave **2j** (eluent: CH_2_Cl_2_; R_f_ = 0.36) in 51% yield as a red powder. Mp: 136 °C. IR: 1023, 1154, 1208, 1236, 1324, 1356, 1379, 1442, 1454, 1478, 1519, 1555, 1577, 1592, 3240, 3339, 3639 cm^−1^. ^1^H-NMR (CDCl_3_): 6.82 (d, 1H, *J* = 3.4 Hz), 7.22–7.44 (m, 8H), 7.53–7.56 (m, 2H), 7.67 (d, 1H, *J* = 8.3 Hz), 7.70–7.72 (m, 3H), 7.86 (dd, 1H, *J* = 4.9 and 1.2 Hz), 7.94 (d, 1H, *J* = 3.4 Hz), 9.17 (d, 1H, *J* = 4.9 Hz). ^13^C-NMR (CDCl_3_): 106.1 (CH), 111.4 (CH), 118.0 (CH), 121.5 (CH), 122.1 (CH), 123.2 (CH), 128.4 (2CH), 128.4 (2CH), 128.5 (C), 129.7 (CH), 129.9 (CH), 130.1 (2CH), 130.3 (C), 130.3 (2CH), 130.5 (C), 130.8 (CH), 136.2 (C), 137.8 (C), 138.0 (C), 144.8 (C), 150.8 (C), 153.6 (CH), 156.4 (C). Anal. Calc. for C_27_H_18_N_4_ (398.47): C 81.39, H 4.55, N, 14.06. Found: C 81.26, H 4.67, N, 13.84.

#### 3.9.4. 2,3-Diphenyl-8-(*N*-pyrazolyl)pyrido[2,3-*b*]pyrazine (**2k**)

The general procedure 6 using 8-iodo-2,3-diphenylpyrido[2,3-*b*]pyrazine (**2b-I**, 0.20 g) and pyrazole (68 mg) gave **2k** (eluent: CH_2_Cl_2_-EtOAc 80:20; R_f_ = 0.47) in 71% yield as a pale yellow powder. Mp: 200 °C. IR: 1027, 1032, 1092, 1164, 1229, 1324, 1356, 1388, 1532, 1549, 1592, 3034, 3060, 3159 cm^−1^. ^1^H-NMR (CDCl_3_): 6.55 (d, 1H, *J* = 2.2 Hz), 7.30–7.41 (m, 6H), 7.55–7.58 (m, 2H), 7.64–7.66 (m, 2H), 7.82 (s, 1H), 8.34 (dd, 1H, *J* = 5.3 and 2.4 Hz), 9.12 (dd, 1H, *J* = 5.2 and 2.2 Hz), 9.46 (t, 1H, *J* = 2.5 Hz). ^13^C-NMR (CDCl_3_): 109.2 (CH), 115.0 (CH), 127.9 (C), 128.3 (2CH), 128.6 (2CH), 129.6 (CH), 129.8 (CH), 129.9 (2CH), 130.3 (2CH), 134.4 (CH), 137.6 (C), 138.3 (C), 142.5 (CH), 143.2 (C), 150.5 (C), 153.4 (C), 154.3 (CH), 156.1 (C). Anal. Calc. for C_22_H_15_N_5_ (349.40): C 75.63, H 4.33, N, 20.04. Found: C 75.71, H 4.42, N, 19.86.

#### 3.9.5. 8-(*N*-imidazolyl)-2,3-diphenylpyrido[2,3-*b*]pyrazine (**2l**)

The general procedure 6 using 8-iodo-2,3-diphenylpyrido[2,3-*b*]pyrazine (**2b-I**, 0.20 g) and imidazole (68 mg) gave **2l** (eluent: EtOAc-MeOH 95:5; R_f_ = 0.48) in 69% yield as a yellow powder. Mp: 209 °C. IR: 1019, 1053, 1075, 1105, 1115, 1169, 1236, 1319, 1334, 1379, 1429, 1446, 1459, 1482, 1549, 1594, 3064, 3124, 3639 cm^−1^. ^1^H-NMR (CDCl_3_): 7.32–7.45 (m, 7H), 7.56 (d, 2H, *J* = 6.6 Hz), 7.65–7.71 (m, 3H), 7.80 (br s, 1H), 8.82 (br s, 1H), 9.20 (d, 1H, *J* = 4.8 Hz). ^13^C-NMR (CDCl_3_): 115.6 (CH), 119.5 (CH), 128.3 (2CH), 128.4 (2CH), 128.9 (C), 129.7 (CH), 129.9 (CH), 129.9 (2CH), 130.1 (2CH), 130.3 (CH), 137.5 (C), 137.6 (C), 138.8 (CH), 141.4 (C), 150.7 (C), 154.1 (C), 154.2 (CH), 156.6 (C). Anal. Calc. for C_22_H_15_N_5_ (349.40): C 75.63, H 4.33, N, 20.04. Found: C 75.74, H 4.37, N, 19.92.

#### 3.9.6. 2,3-Diphenyl-8-[1-(1,2,4-triazolyl)]pyrido[2,3-*b*]pyrazine (**2m**)

The general procedure 6 using 8-iodo-2,3-diphenylpyrido[2,3-*b*]pyrazine (**2b-I**, 0.20 g) and 1,2,4-triazole (69 mg) gave **2m** (eluent: CH_2_Cl_2_-EtOAc 80:20; R_f_ = 0.35) in 79% yield as an orange powder. Mp: 205 °C. IR: 708, 995, 1025, 1049, 1079, 1124, 1158, 1223, 1242, 1276, 1332, 1386, 1403, 1459, 1508, 1551, 1590, 3064, 3146 cm^−1^. ^1^H-NMR (CDCl_3_): 7.35–7.49 (m, 6H), 7.59 (d, 2H, *J* = 7.0 Hz), 7.68 (d, 2H, *J* = 7.2 Hz), 8.22 (s, 1H), 8.37 (br s, 1H), 9.27 (br s, 1H), 10.16 (br s, 1H). ^13^C-NMR (CDCl_3_): 115.1 (CH), 127.3 (C), 128.2 (2CH), 128.5 (2CH), 129.8 (CH), 129.8 (2CH), 129.9 (CH), 130.1 (2CH), 137.3 (C), 137.7 (C), 140.4 (C), 147.2 (CH), 150.4 (C), 152.0 (CH), 154.1 (C), 154.5 (CH), 156.7 (C). Anal. Calc. for C_21_H_14_N_6_ (350.39): C 71.99, H 4.03, N, 23.99. Found: C 72.19, H 4.15, N, 23.81.

#### 3.9.7. 5-Iodo-2,3-diphenyl-8-(*N*-pyrazolyl)quinoxaline (**1k′**)

The general procedure 6 using 5-iodo-2,3-diphenylquinoxaline (**1b′**, 0.27 g) and pyrazole (68 mg) gave **1k′** (eluent: CH_2_Cl_2_-heptane 80:20; R_f_ = 0.45) in 50% yield as an pale yellow powder. Mp: 200–202 °C. IR: 536, 585, 602, 692, 696, 755, 843, 894, 946, 1040, 1092, 1182, 1193, 1221, 1336, 1397, 1465, 1519, 1543, 1592, 3060, 3159 cm^−1^. ^1^H-NMR (CDCl_3_): 6.56 (dd, 1H, *J* = 2.6, 1.8 Hz), 7.35–7.45 (m, 6H), 7.57–7.60 (m, 2H), 7.70–7.73 (m, 2H), 7.82 (d, 1H, *J* = 1.8 Hz), 8.12 (d, 1H, *J* = 8.2 Hz), 8.43 (d, 1H, *J* = 8.3 Hz), 8.97–8.98 (m, 1H). ^13^C-NMR (CDCl_3_): 99.3 (C), 107.6 (CH), 123.7 (CH), 128.4 (2CH), 128.5 (2CH), 129.5 (CH), 129.6 (CH), 130.0 (2CH), 130.4 (2CH), 133.2 (C), 133.5 (CH), 137.1 (C), 137.8 (C), 138.2 (C), 139.7 (CH), 140.7 (C), 141.1 (CH), 153.0 (C), 153.6 (C). Anal. Calc. for C_23_H_15_IN_4_ (474.31): C 58.24, H 3.19, N, 11.81. Found: C 58.33, H 3.26, N, 11.68.

### 3.10. Nucleophilic Substitution Using Amines

#### 3.10.1. General Procedure 7

A sealed tube containing the iodide (0.50 mmol) and amine (amount given in the product description) in ethanol (2 mL) was heated (conditions given in the product description). The cooled residue was concentrated before chromatography over silica gel (eluent given in the product description).

#### 3.10.2. 8-(Isopropylamino)-2,3-diphenylpyrido[2,3-*b*]pyrazine (**2n**)

The general procedure 7 (150 °C, 18 h) using 8-iodo-2,3-diphenylpyrido[2,3-*b*]pyrazine (**2b-I**, 0.20 g) and isopropylamine (51 μL, 0.60 mmol) gave **2n** (eluent: CH_2_Cl_2_-EtOAc 50:50; R_f_ = 0.20) in 69% yield as a beige powder. Mp: 179 °C. IR: 699, 703, 772, 804, 1156, 1178, 1236, 1313, 1336, 1538, 1564, 1592, 2965, 3038, 3064, 3390 cm^−1^. ^1^H-NMR (CDCl_3_): 1.27 (d, 6H, *J* = 6.4 Hz, Me), 3.77 (dp, 1H, *J* = 7.9 and 6.4 Hz, C*H*Me_2_), 6.40 (br d, 1H, *J* = 8.0 Hz, NH), 6.45 (d, 1H, *J* = 5.5 Hz), 7.15–7.28 (m, 6H), 7.39–7.42 (m, 2H), 7.46–7.49 (m, 2H), 8.59 (dd, 1H, *J* = 5.4, 0.6 Hz). ^13^C-NMR (CDCl_3_): 22.3 (2CH_3_), 44.1 (CH), 100.7 (CH), 127.1 (C), 128.0 (2CH), 128.2 (2CH), 128.7 (CH), 129.0 (CH), 129.9 (2CH), 130.2 (2CH), 138.4 (C), 138.9 (C), 149.8 (C), 150.1 (C), 150.1 (C), 154.8 (CH), 155.8 (C). Anal. Calc. for C_22_H_20_N_4_ (340.43): C 77.62, H 5.92, N, 16.46. Found: C 77.72, H 6.14, N, 16.19.

#### 3.10.3. 8-(4-Methoxybenzylamino)-2,3-diphenylpyrido[2,3-*b*]pyrazine (**2o**)

The general procedure 7 (150 °C, 24 h) using 8-iodo-2,3-diphenylpyrido[2,3-*b*]pyrazine (**2b-I**, 0.20 g) and 4-methoxybenzylamine (78 μL, 0.60 mmol) gave **2o** (eluent: CH_2_Cl_2_-EtOAc 50:50; R_f_ = 0.48) in 71% yield as a yellow powder. Mp: 190 °C. IR: 697, 832, 1175, 1236, 1302, 1341, 1437, 1459, 1510, 1585, 2828, 2910, 3064, 3232 cm^−1^. ^1^H-NMR (CDCl_3_): 3.81 (s, 3H, OMe), 4.55 (d, 2H, *J* = 5.9 Hz), 6.58 (d, 1H, *J* = 5.3 Hz), 6.90 (d, 2H, *J* = 8.7 Hz), 7.02 (t, 1H, *J* = 5.7 Hz), 7.27–7.35 (m, 8H), 7.49–7.51 (m, 2H), 7.58–7.61 (m, 2H), 8.69 (d, *J* = 5.3 Hz, 1H). ^13^C-NMR (CDCl_3_): 46.5 (CH_2_), 55.3 (CH_3_), 101.1 (CH), 114.3 (2CH), 127.2 (C), 128.0 (2CH), 128.2 (2CH), 128.6 (2CH), 128.8 (CH), 129.1 (CH), 129.1 (C), 129.9 (2CH), 130.3 (2CH), 138.5 (C), 138.8 (C), 150.0 (C), 150.2 (C), 150.9 (C), 154.9 (CH), 155.9 (C), 159.2 (C). Anal. Calc. for C_27_H_22_N_4_O (418.50): C 77.49, H 5.30, N, 13.39. Found: C 77.58, H 5.44, N, 13.20.

#### 3.10.4. 8-(Benzylamino)-2,3-diphenylpyrido[2,3-*b*]pyrazine (**2p**)

The general procedure 7 (150 °C, 24 h) using 8-iodo-2,3-diphenylpyrido[2,3-*b*]pyrazine (**2b-I**, 0.20 g) and benzylamine (66 μL, 0.60 mmol) gave **2p** (eluent: CH_2_Cl_2_-EtOAc 50:50; R_f_ = 0.50) in 79% yield as a yellow powder. Mp: 238 °C. IR: 697, 768, 873, 1150, 1238, 1300, 1324, 1339, 1439, 1538, 1590, 2910, 3064, 3201 cm^−1^. ^1^H-NMR (CDCl_3_): 4.63 (d, 2H, *J* = 6.0 Hz), 6.56 (d, 1H, *J* = 5.4 Hz), 7.11 (t, 1H, *J* = 6.0 Hz), 7.27–7.39 (m, 11H), 7.49–7.52 (m, 2H), 7.58–7.61 (m, 2H), 8.68 (d, 1H, *J* = 5.4 Hz). ^13^C-NMR (CDCl_3_): 47.0 (CH_2_), 101.2 (CH), 127.2 (2CH), 127.2 (C), 127.8 (CH), 128.1 (2CH), 128.3 (2CH), 128.9 (CH), 128.9 (2CH), 129.1 (CH), 129.9 (2CH), 130.3 (2CH), 137.2 (C), 138.5 (C), 138.8 (C), 150.0 (C), 150.3 (C), 151.0 (C), 154.9 (CH), 156.0 (C). *Crystal data for* **2p**. C_26_H_20_N_4_, *M* = 388.46, *T* = 150(2) K, monoclinic, *P* 2_1_/*n*, *a* = 6.0721(6), *b* = 12.8640(10), *c* = 25.460(2) Å, β = 91.436(4) °, *V* = 1988.1(3) Å^3^, *Z* = 4, *d* = 1.298 g cm^−3^, *μ* = 0.078 mm^−1^. A final refinement on *F*^2^ with 4438 unique intensities and 274 parameters converged at ω*R*(*F*^2^) = 0.1432 (*R*(*F*) = 0.0626) for 3710 observed reflections with *I* > 2σ(*I*). CCDC 1858476.

### 3.11. Nucleophilic Substitution using Hydrazine Hydrate: 8-Hydrazino-2,3-diphenylpyrido[2,3-b]pyrazine (2q)

A solution of 8-iodo-2,3-diphenyl pyrido [2,3-*b*]pyrazine (**2b-I**, 0.20 g, 0.50 mmol) and hydrazine hydrate (0.25 mL, 5.0 mmol) in isopropanol (2 mL) was heated under reflux for 4 h. The cooled residue was concentrated and taken up with EtOAc (20 mL). The organic layer was washed with water (10 mL), dried over MgSO_4_, filtered and concentrated under reduced pressure to give the title compound **2q** in 92% yield as a red powder. Mp > 250 °C. ^1^H-NMR (CDCl_3_): 4.22 (br s, 2H, NH), 6.91 (d, 1H, *J* = 5.6 Hz), 7.22–7.36 (m, 7H), 7.40–7.43 (m, 2H), 7.48–7.51 (m, 2H), 8.60 (d, 1H, *J* = 5.6 Hz). ^13^C-NMR (CDCl_3_): 101.2 (CH), 126.2 (C), 128.2 (2CH), 128.3 (2CH), 129.0 (CH), 129.2 (CH), 129.9 (2CH), 130.3 (2CH), 138.4 (C), 138.7 (C), 149.7 (C), 150.3 (C), 153.0 (C), 155.0 (CH), 156.1 (C). Anal. Calc. for C_19_H_15_N_5_ (313.36): C 72.83, H 4.83, N, 22.35. Found: C 72.96, H 4.89, N, 22.31.

### 3.12. Condensation Reactions from the Hydrazine 2q

#### 3.12.1. General Procedure 8

A sealed tube containing 8-hydrazino-2,3-diphenylpyrido[2,3-*b*] pyrazine (**2q**, 0.16 g, 0.50 mmol) and the aldehyde (0.55 mmol) in ethanol (2 mL) was heated at 110 °C overnight. The cooled residue was concentrated under vacuum, washed with methanol and isolated by filtration.

#### 3.12.2. 2-Hydroxybenzaldehyde 2-[8-(2,3-diphenylpyrido[2,3-*b*]pyrazinyl)]hydrazone (**2r**)

General Procedure 8 using 2-hydroxybenzaldehyde (67 mg) gave **2r** (R_f_ (CH_2_Cl_2_-EtOAc 80:20) = 0.44) in 60% yield as a yellow powder. Mp > 260 °C. IR: 952, 1019, 1096, 1163, 1233, 1270, 1309, 1328, 1422, 1540, 1562, 1594, 1618, 3064, 3317 cm^−1^. ^1^H-NMR (CDCl_3_): 6.96 (td, 1H, *J* = 7.5 and 1.1 Hz), 7.07 (d, 1H, *J* = 8.2 Hz), 7.23–7.44 (m, 9H), 7.51–7.54 (m, 2H), 7.58–7.62 (m, 2H), 8.26 (s, 1H), 8.91 (d, 1H, *J* = 5.3 Hz), 9.71 (br s, 1H), 10.60 (br s, 1H). The ^13^C spectra could not be recorded due to low solubility in CDCl_3_ and DMSO. Anal. Calc. for C_26_H_19_N_5_O (417.47): C 74.80, H 4.59, N, 16.78. Found: C 74.72, H 4.39, N, 16.67.

#### 3.12.3. Piperonal 2-[8-(2,3-diphenylpyrido[2,3-*b*]pyrazinyl)]hydrazone (**2s**)

General Procedure 8 using piperonal (83 mg) gave **2s** (R_f_ (CH_2_Cl_2_-EtOAc 80:20) = 0.37) in 70% yield as a yellow powder. Mp: 254 °C. IR: 933, 1038, 1150, 1255, 1339, 1450, 1489, 1501, 1545, 1568, 1590, 2901, 3060, 3322, 3648 cm^−1^. ^1^H-NMR (CDCl_3_): 6.03 (s, 2H), 6.84 (d, 1H, *J* = 8.0 Hz), 7.07 (dd, 1H, *J* = 8.1 and 1.6 Hz), 7.28–7.42 (m, 7H), 7.50 (t, 3H, *J* = 6.6 Hz), 7.59 (d, 2H, *J* = 6.8 Hz), 7.97 (s, 1H), 8.85 (d, 1H, *J* = 5.3 Hz), 9.66 (s, 1H). ^13^C-NMR ((CD_3_)_2_SO): 101.5 (CH_2_), 103.5 (CH), 104.9 (CH), 108.5 (CH), 123.0 (CH), 125.6 (C), 128.1 (2CH), 128.2 (2CH), 128.8 (CH), 129.1 (CH), 129.2 (C), 129.7 (2CH), 130.1 (2CH), 138.3 (C), 138.6 (C), 145.1 (CH), 147.7 (C), 148.1 (C), 148.8 (C), 149.6 (C), 150.0 (C), 154.5 (CH), 155.5 (C). Anal. Calc. for C_27_H_19_N_5_O_2_ (445.48): C 72.80, H 4.30, N, 15.72. Found: C 72.95, H 4.44, N, 15.83.

#### 3.12.4. 2-Hydroxy-4-methoxybenzaldehyde 2-[8-(2,3-diphenylpyrido[2,3-*b*]pyrazinyl)]hydrazone (**2t**)

General Procedure 8 using 2-hydroxy-4-methoxybenzaldehyde (84 mg) gave **2t** (R_f_ (CH_2_Cl_2_- EtOAc 80:20) = 0.58) in 80% yield as a yellow powder. Mp > 260 °C. IR: 1032, 1135, 1163, 1238, 1291, 1339, 1431, 1439, 1461, 1510, 1543, 1566, 1631, 2845, 2931, 3004, 3056, 3176, 3317 cm^−1^. ^1^H-NMR (CDCl_3_): 3.85 (s, 3H), 6.52 (dd, 1H, *J* = 8.5 and 2.5 Hz), 6.58 (d, 1H, *J* = 2.5 Hz), 7.15 (d, 1H, *J* = 8.6 Hz), 7.19 (d, 1H, *J* = 5.2 Hz), 7.28–7.43 (m, 6H), 7.50–7.54 (m, 2H), 7.58–7.61 (m, 2H), 8.19 (s, 1H), 8.88 (br s, 1H), 9.59 (br s, 1H), 10.81 (s, 1H). The ^13^C spectra could not be recorded due to low solubility in CDCl_3_ and DMSO. Anal. Calc. for C_27_H_21_N_5_O_2_ (447.50): C 72.47, H 4.73, N, 15.65. Found: C 72.53, H 4.89, N, 15.60.

#### 3.12.5. 4-(Trifluoromethyl)benzaldehyde 2-[8-(2,3-diphenylpyrido[2,3-*b*]pyrazinyl)]hydrazone (**2u**)

General Procedure 8 using 4-(trifluoromethyl)benzaldehyde (87 mg) gave **2u** (R_f_ (CH_2_Cl_2_- EtOAc 80:20) = 0.51) in 73% yield as a yellow powder. Mp: 258–260 °C. IR: 1017, 1066, 1109, 1124, 1145, 1236, 1300, 1321, 1512, 1545, 1562, 1588, 3060, 3184, 3317, 3652 cm^−1^. ^1^H-NMR (CDCl_3_): 7.28–7.43 (m, 6H), 7.50–7.53 (m, 2H), 7.56–7.61 (m, 3H), 7.68 (d, 2H, *J* = 8.2 Hz), 7.87 (d, 2H, *J* = 7.8 Hz), 8.11 (s, 1H), 8.91 (d, 1H, *J* = 5.2 Hz), 9.90 (br s, 1H). ^13^C-NMR ((CD_3_)_2_SO, 333 K): 103.8 (CH), 124.0 (q, CF_3_, *J* = 272 Hz), 125.4 (C), 125.5 (q, 2CH, *J* = 3.7 Hz), 127.1 (2CH), 127.8 (2CH), 127.9 (2CH), 128.7 (CH), 128.8 (CH), 129.2 (q, *C*-CF_3_, *J* = 31.7 Hz), 129.5 (2CH), 129.8 (2CH), 138.1 (C), 138.4 (C), 138.5 (C), 143.2 (CH), 147.4 (C), 149.4 (C), 150.3 (C), 154.3 (CH), 155.5 (C). Anal. Calc. for C_27_H_18_F_3_N_5_ (469.47): C 69.08, H 3.86, N, 14.92. Found: C 69.25, H 3.97, N, 14.78.

### 3.13. Nucleophilic Substitution Using a Phenolate: Methyl 2-[8-(2,3-diphenylpyrido[2,3-b]pyrazinyl)]oxy- 5-methoxybenzoate (**2v**)

A mixture of 8-iodo-2,3-diphenylpyrido[2,3-*b*]pyrazine (**2b-I**, 0.20 g, 0.50 mmol), methyl 2-hydroxy-5-methoxy-benzoate (0.10 g, 0.55 mmol), K_2_CO_3_ (77 mg, 0.55 mmol) and DMSO (1 mL) was heated at 110 °C for 2 h. The cooled residue was treated by an aqueous solution of Na_2_CO_3_ (10 mL) before extraction with Et_2_O (3 × 10 mL). The organic layer was dried over MgSO_4_, filtered and concentrated under reduced pressure, and the residue was chromatographed over silica gel (eluent: CH_2_Cl_2_-MeOH 95:5; Rf (CH_2_Cl_2_-EtOAc 95:5) = 0.50) to give the title compound **2v** in 64% yield as a beige powder. Mp: 206 °C. IR: 542, 698, 773, 856, 1021, 1072, 1109, 1205, 1235, 1263, 1333, 1350, 1434, 1468, 1496, 1554, 1594, 1719, 2845, 2956, 3041 cm^−1^. ^1^H-NMR (CDCl_3_): 3.65 (s, 3H), 3.90 (s, 3H), 6.64 (d, *J* = 5.2 Hz, 1H), 7.19 (dd, 1H, *J* = 8.9, 3.0 Hz), 7.24 (d, 1H, *J* = 9.5 Hz), 7.30–7.40 (m, 6H), 7.55–7.58 (m, 3H), 7.61–7.64 (m, 2H), 8.86 (d, 1H, *J* = 5.2 Hz). ^13^C-NMR (CDCl_3_): 52.4 (CH_3_), 56.0 (CH_3_), 107.6 (CH), 116.3 (CH), 120.6 (CH), 124.6 (C), 125.0 (CH), 128.2 (2CH), 128.4 (2CH), 129.0 (C), 129.1 (CH), 129.4 (CH), 130.2 (2CH), 130.3 (2CH), 138.2 (C), 138.7 (C), 147.0 (C), 151.1 (C), 153.6 (C), 154.4 (CH), 156.6 (C), 157.4 (C), 163.0 (C), 164.7 (C). Anal. Calc. for C_28_H_21_N_3_O_4_ (463.49): C 72.56, H 4.57, N, 9.07. Found: C 72.49, H 4.65, N, 9.01.

## 4. Conclusions

Original pyrazino-fused polycyclic scaffolds were synthesized by combining deproto- metalation-iodolysis with palladium- or copper-catalyzed couplings or direct substitution reactions. This study highlights the interest in preparing iodo derivatives of sensitive aromatic heterocycles by using lithium-zinc basic combinations to access scaffolds of potential biological interest. Interestingly, bromine and trichloroisocyanuric acid were successfully employed as electrophiles to intercept the intermediate heteroarylzinc halides.

## Figures and Tables

**Table 1:**
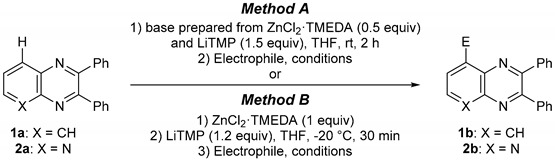
Deprotonative metalation of 2,3-diphenylquinoxaline (**1a**) and 2,3-diphenylpyrido[2,3-*b*]-pyrazine (**2a**) and conversion to the halogeno derivatives.

Entry	Substrate	*Method*	Electrophile, Conditions		Product (E), Yield (%) ^1^	
1	**1a** (X = CH)	**A**	I_2_, THF, 0 °C, 1 h		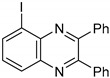	**1b** (I), 74 ^2^
2	**1a** (X = CH)	**B**	I_2_, THF, 0 °C, 1 h			**1b** (I), 70
3	**2a** (X = N)	**A**	I_2_, THF, 0 °C, 1 h		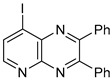	**2b-I** (I), 70
4	**2a** (X = N)	**B**	I_2_, THF, 0 °C, 1 h			**2b-I** (I), 62
5	**2a** (X = N)	**B**	Br_2_, −20 °C, 1 h		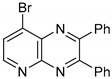	**2b-Br** (Br), 60
6	**2a** (X = N)	**B**	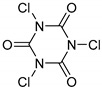	−20 °C, 1 h	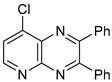	**2b-Cl** (Cl), 62

^1^ After purification (see experimental part). ^2^ The rest is 5,8-diiodo-2,3-diphenylquinoxaline (**1b′**; 7% yield; see Figure 1). **1b′** was isolated in 70% yield by using ZnCl_2_·TMEDA (1 equiv) and LiTMP (3 equiv).

**Table 2:**
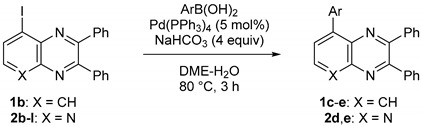
Suzuki coupling from 5-iodo-2,3-diphenylquinoxaline (**1b**) and 8-iodo-2,3-diphenyl pyrido[2,3-*b*]pyrazine (**2b-I**).

Entry	Substrate	ArB(OH)_2_	Product (Ar), Yield (%) ^1^
1	**1b** (X = CH)	PhB(OH)_2_	**1c** (Ph), 42
2	**1b** (X = CH)	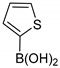	**1d** (2-thienyl), 97
3	**2b-I** (X = N)		**2d** (2-thienyl) ^2^, 75
4	**1b** (X = CH)	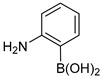	**1e** (2-aminophenyl), 92
5	**2b-I** (X = N)		**2e** (2-aminophenyl), 73

^1^ After purification (see experimental part). ^2^ See Figure 1.

**Table 3:**
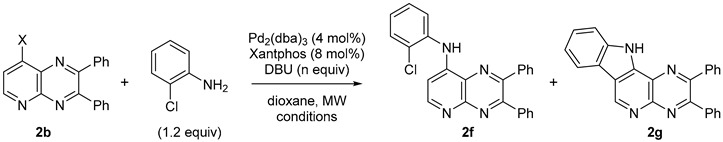
Study of the conversion of the 8-halogenated 2,3-diphenylpyrido[2,3-*b*]pyrazines **2b** into 2,3-diphenyl-11*H*-pyrazino[2′,3′:5,6]pyrido[4,3-*b*]indole (**2g**) under MW irradiation.

Entry	Substrate (X)	n Equiv	Conditions ^1^	2f (%) ^2^	2g (%) ^2^
1	**2b-I** (I)	1.2	180 °C, 20 min	80	20
2	**2b-I** (I)	3.0	180 °C, 20 min	41	59
3	**2b-I** (I)	3.0	180 °C, 60 min	<5	>95, 70 ^3^
4	**2b-I** (I)	3.0	180 °C, 60 min ^4^	75	25
5	**2b-I** (I)	3.0	180 °C, 20 min ^5^	23	75
6 ^6^	**2b-I** (I)	3.0	180 °C, 20 min	95	<2
7 ^7^	**2b-I** (I)	3.0	180 °C, 20 min	32	67
8	**2b-I** (I)	3.0	180 °C, 5 min	66 ^8^	0
9	**2b-Br** (Br)	3.0	180 °C, 5 min	35 ^9^	0
10	**2b-Cl** (Cl)	3.0	180 °C, 5 min	22 ^10^	0

^1^ Maximum microwave power applied: 150–200 W at the beginning to reach the required temperature. ^2^ Evaluated from the NMR spectra of the crudes. ^3^ Yield after purification. ^4^ Microwave profile of irradiation: The sequence ‘*Maximum microwave power applied: 150*–*200 W to reach 180 °C then 2 min at 180 °C before cooling to 100 °C*’ was repeated every 4 min. ^5^ Then classical heating at 180 °C for 40 min. ^6^ Without catalyst. ^7^ By using 12 mol% Pd_2_(dba)_3_ and 30 mol% Xantphos. ^8^ The rest was unreacted **2b-I** (32%). ^9^ The rest was unreacted **2b-Br** (38%) and **2a** (28%). ^10^ The rest was unreacted **2b-Cl** (78%).

**Table 4:**
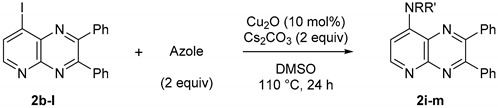
Copper-catalyzed *N*-arylation of 8-iodo-2,3-diphenylpyrido[2,3-*b*]pyrazine (**2b-I**) using azoles.

Entry	Azole	Product, Yield (%) ^1^	
1	Pyrrole	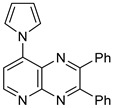	**2i**, 67
2	Indole	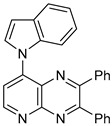	**2j**, 51
3	Pyrazole	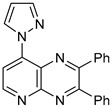	**2k**, 71
4	Imidazole	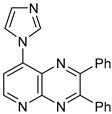	**2l**, 69
5	1,2,4-Triazole	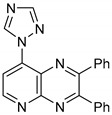	**2m**, 79

^1^ After purification (see experimental part). The rest is starting material and the corresponding deiodinated compound.

**Table 5:**
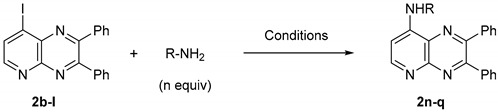
Conversion of 8-iodo-2,3-diphenylpyrido[2,3-*b*]pyrazine (**2b-I**) into corresponding amines and hydrazine.

Entry	R-NH_2_	Conditions	Product, Yield (%) ^1^	
1	iPrNH_2_ (1.2)	EtOH, 150 °C, 18 h	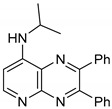	**2n**, 69
2	4-MeOC_6_H_4_CH_2_NH_2_ (1.2)	EtOH, 150 °C, 24 h	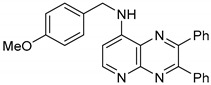	**2o**, 71
3	PhCH_2_NH_2_ (1.2)	EtOH, 150 °C, 24 h	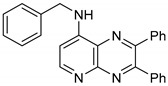	**2p**^2^, 79
4	NH_2_NH_2_·H_2_O (10)	iPrOH, reflux, 4 h	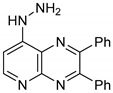	**2q**, 92

^1^ After purification (see the Materials and Methods section). ^2^ See Figure 1.

## Supplementary Materials

Supplementary materials are available online.
