# Supplementary material for: From Quinoxaline, Pyrido[2,3-b]pyrazine and Pyrido[3,4-b]pyrazine to Pyrazino-Fused Carbazoles and Carbolines
Source: Molecules. 2018 Nov 13;23(11):2961. doi: 10.3390/molecules23112961 (PMC6278407; doi:10.3390/molecules23112961)
Supplement: Supplementary file 1 [file molecules-23-02961-s001.pdf]

Supplementary Materials

From quinoxaline, pyrido[2,3-*b*]pyrazine and pyrido[3,4-*b*]pyrazine to pyrazino-fused carbazoles and carbolines

Frédéric Lassagne [1,*] Timothy Langlais [1], Elsa Caytan [1], Emmanuelle Limanton [1], Ludovic Paquin [1,*], Manon Boullard [1], Coline Courtel [1], Idriss Curbet [1], Clément Gédéon [1], Julien Lebreton [1], Laurent Picot [2,*], Valérie Thiéry [2], Mohamed Souab [3], Blandine Baratte [3], Sandrine Ruchaud [3], Stéphane Bach [3,*], Thierry Roisnel [1] and Florence Mongin [1,*]

[1] *Univ Rennes, CNRS, ISCR (Institut des Sciences Chimiques de Rennes) - UMR 6226, F-35000 Rennes, France*

[2] *Laboratoire Littoral Environnement et Sociétés, UMRi CNRS 7266, Université de La Rochelle, 17042 La Rochelle, France*

[3] *Sorbonne Universités, UPMC Univ Paris 06, CNRS USR3151, "Protein Phosphorylation and Human Disease" Unit, Plateforme de criblage KISSf, Station Biologique de Roscoff, Place Georges Teissier, 29688 Roscoff, France*

frederic.lassagne@univ-rennes1.fr (F. Lassagne)

ludovic.paquin@univ-rennes.fr (L. Paquin)

laurent.picot@univ-lr.fr (L. Picot)

bach@sb-roscoff.fr (S. Bach)

florence.mongin@univ-rennes1.fr (F. Mongin).

NMR data of compounds 1b-g, 1b', 1k', 2b-I, 2b-Br, 2b-Cl, 2d-g, 2i-v, 3b, 3g', 3h, 4a and 4b

Inhibitory activities of synthesized compounds against a short panel of disease-related protein kinases

NMR data of compounds 1b-g, 1b’, 1k’, 2b-I, 2b-Br, 2b-Cl, 2d-g, 2i-v, 3b, 3g’, 3h, 4a and 4b

Compound 1b

^{1}H NMR (300 MHz, $CDCl_3$)

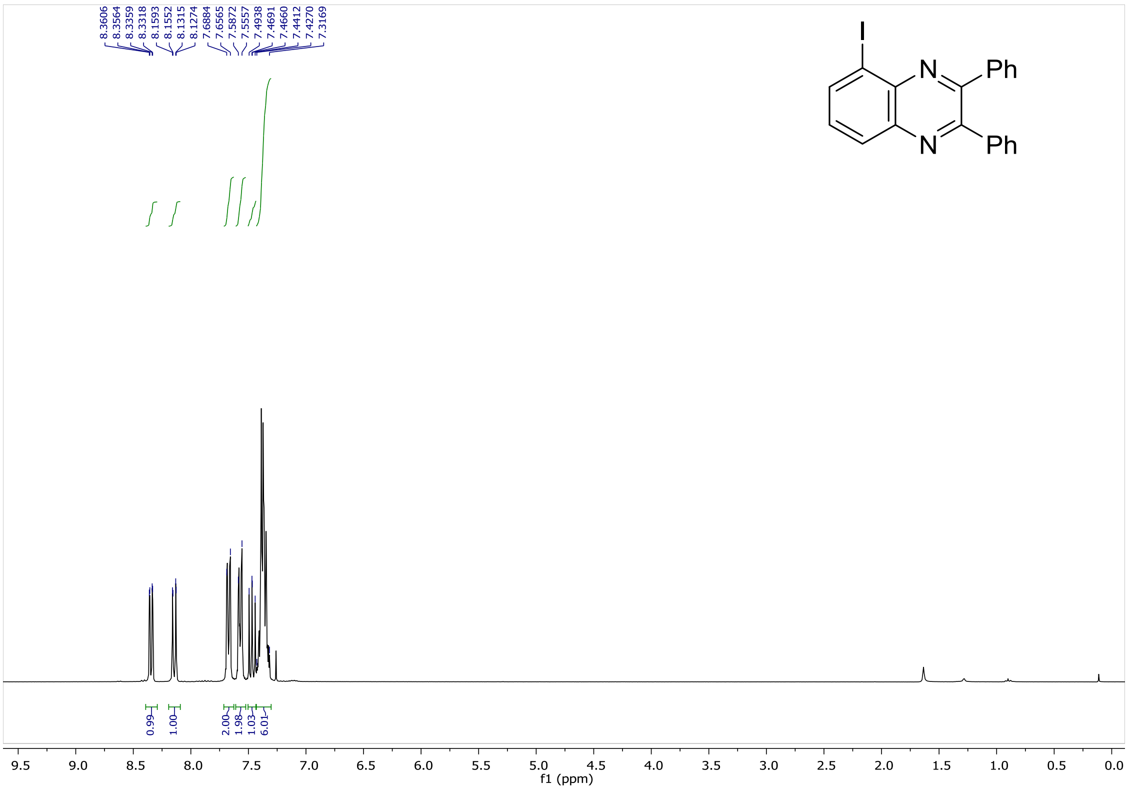

^{13}C NMR (75 MHz, $CDCl_3$)

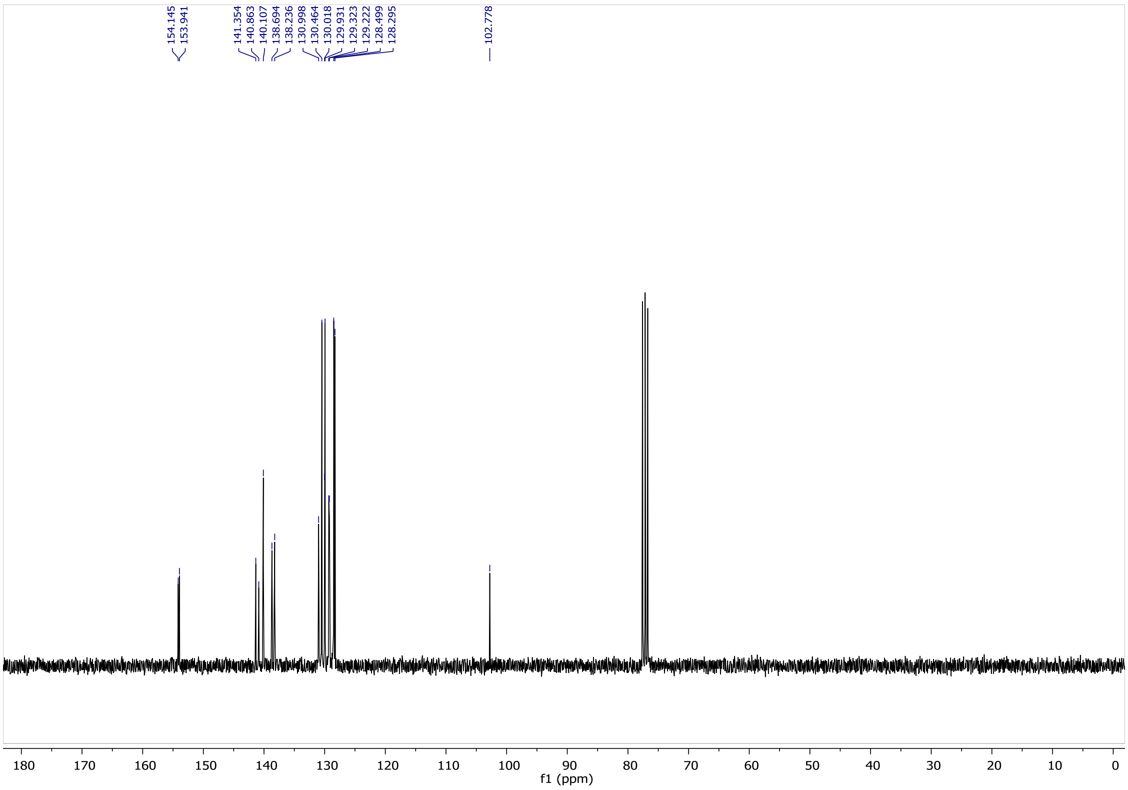

Compound 1b’

^{1}H NMR (300 MHz, CDCl$_3$)

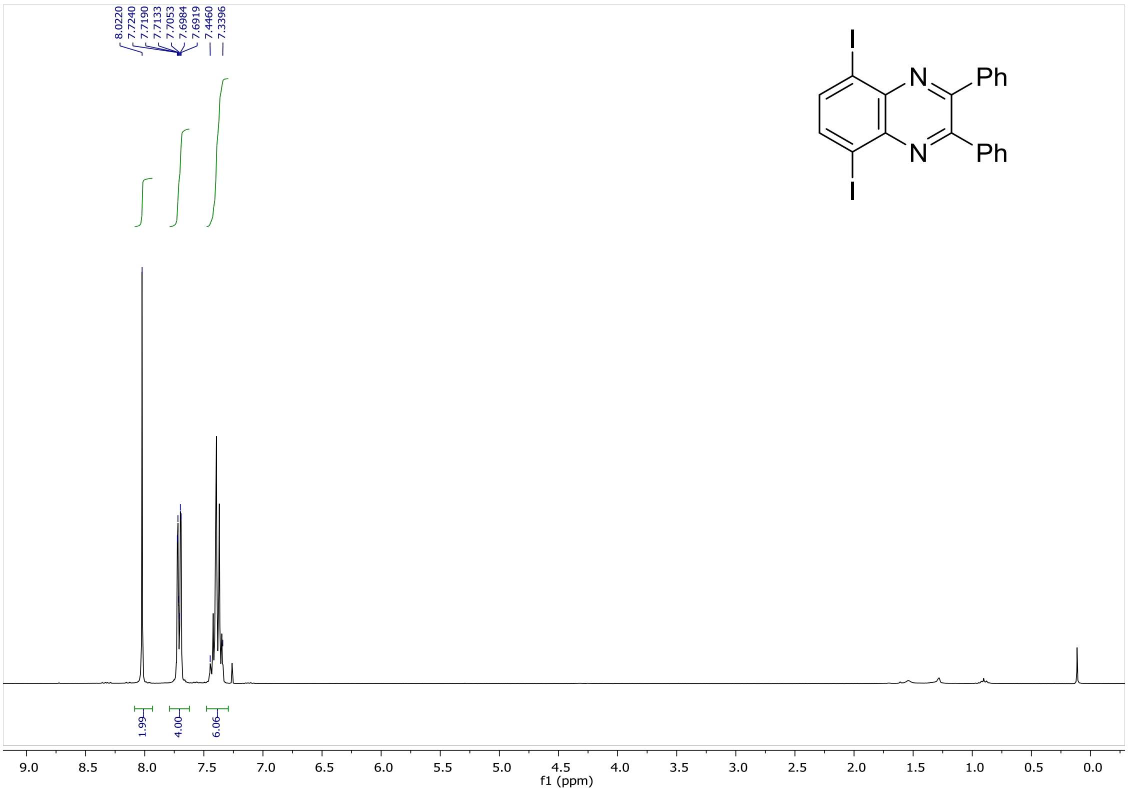

^{13}C NMR (75 MHz, CDCl$_3$)

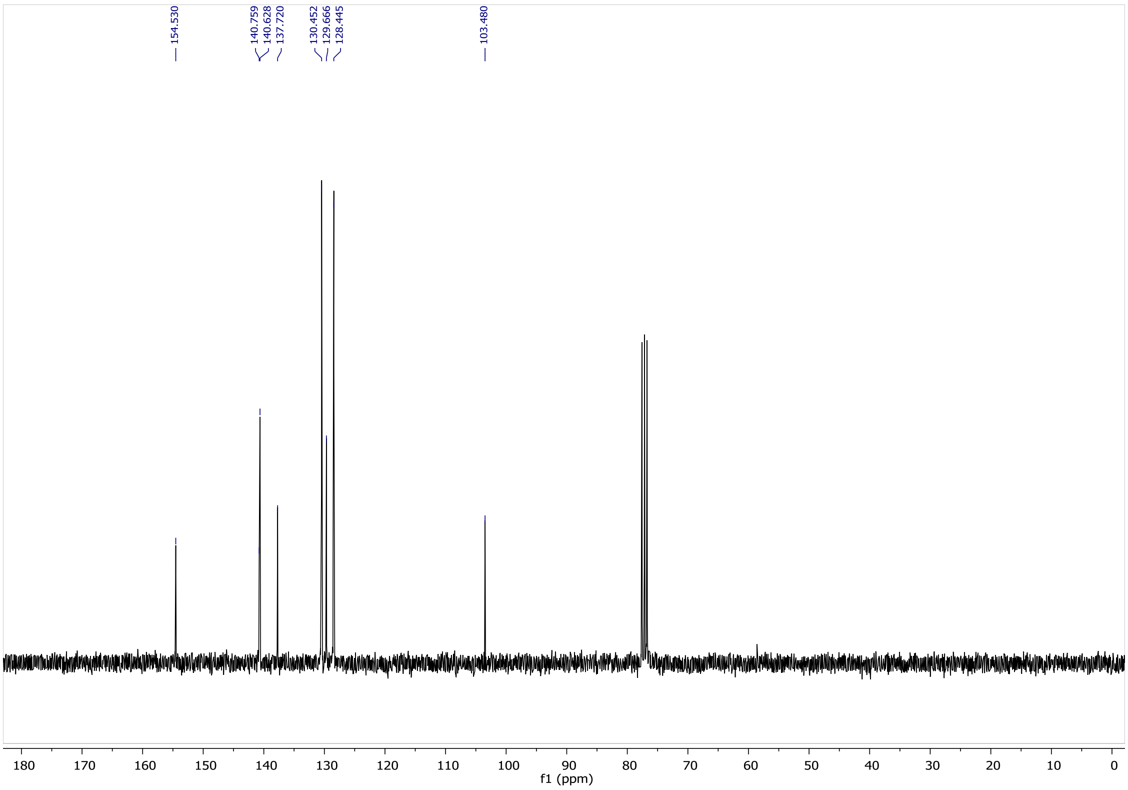

Compound 2b-I

^{1}H NMR (300 MHz, $CDCl_3$)

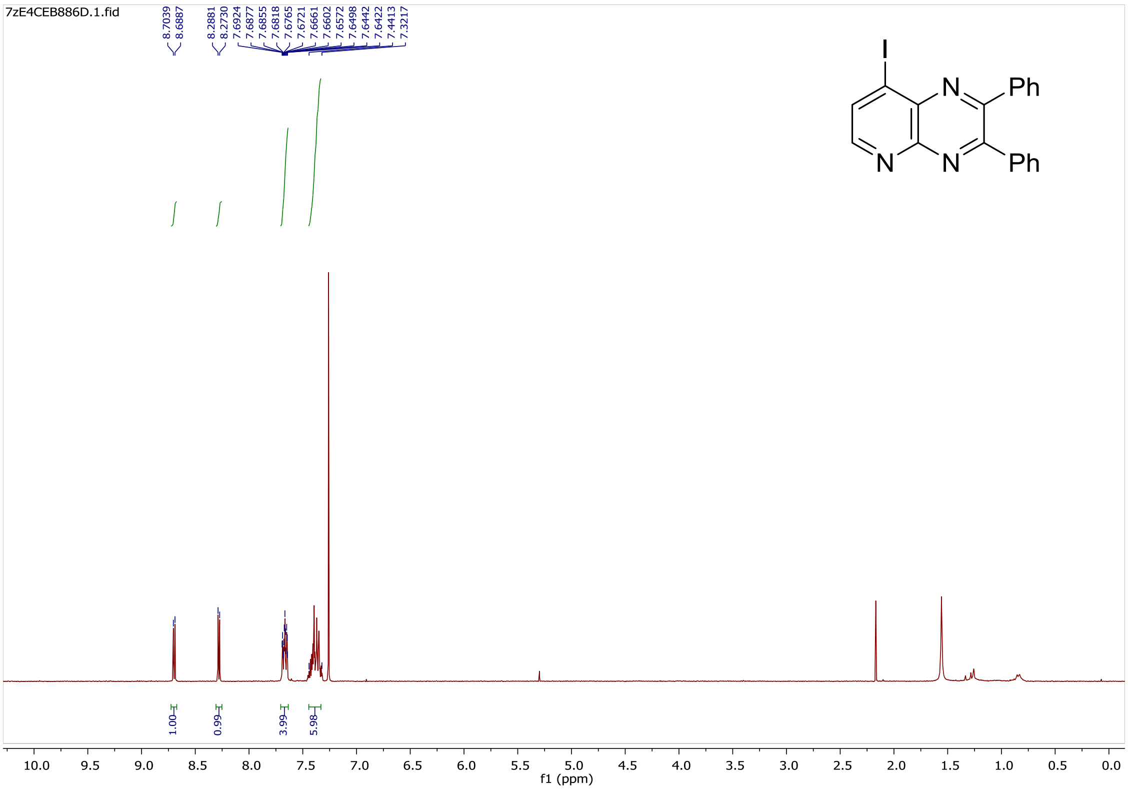

^{13}C NMR (75 MHz, $CDCl_3$)

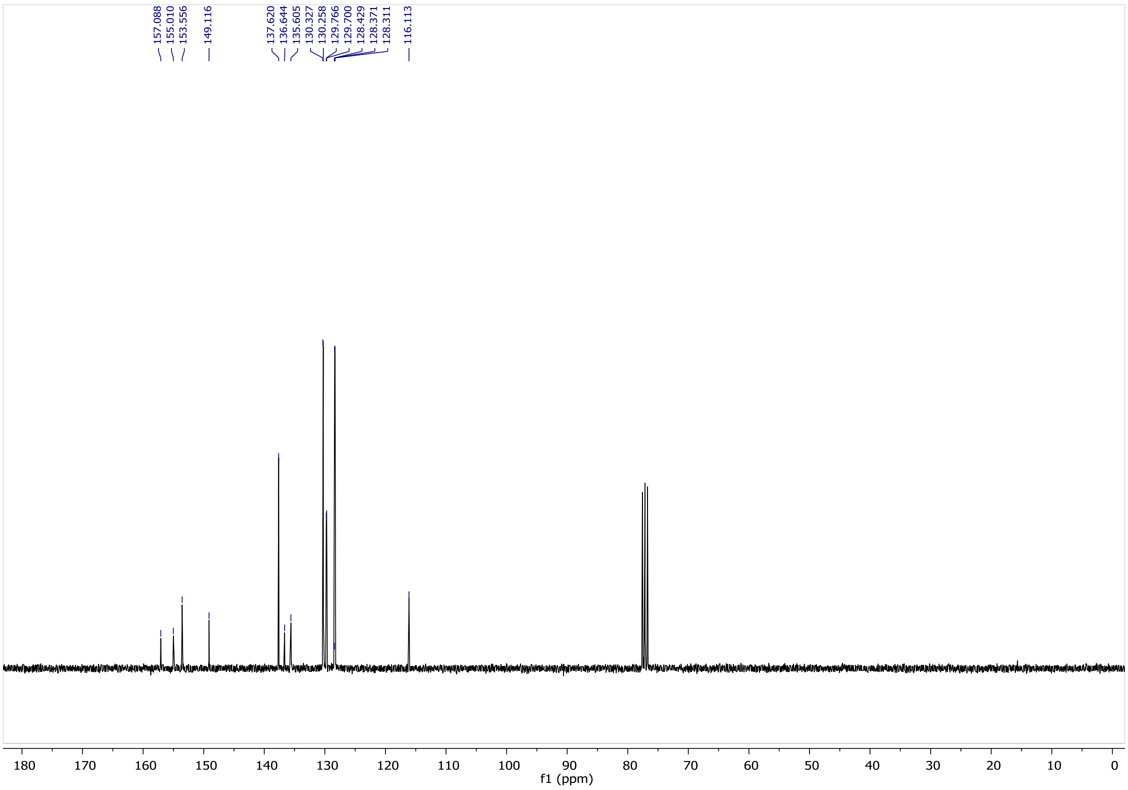

Compound 2b-Br

^{1}H NMR (300 MHz, CDCl$_3$)

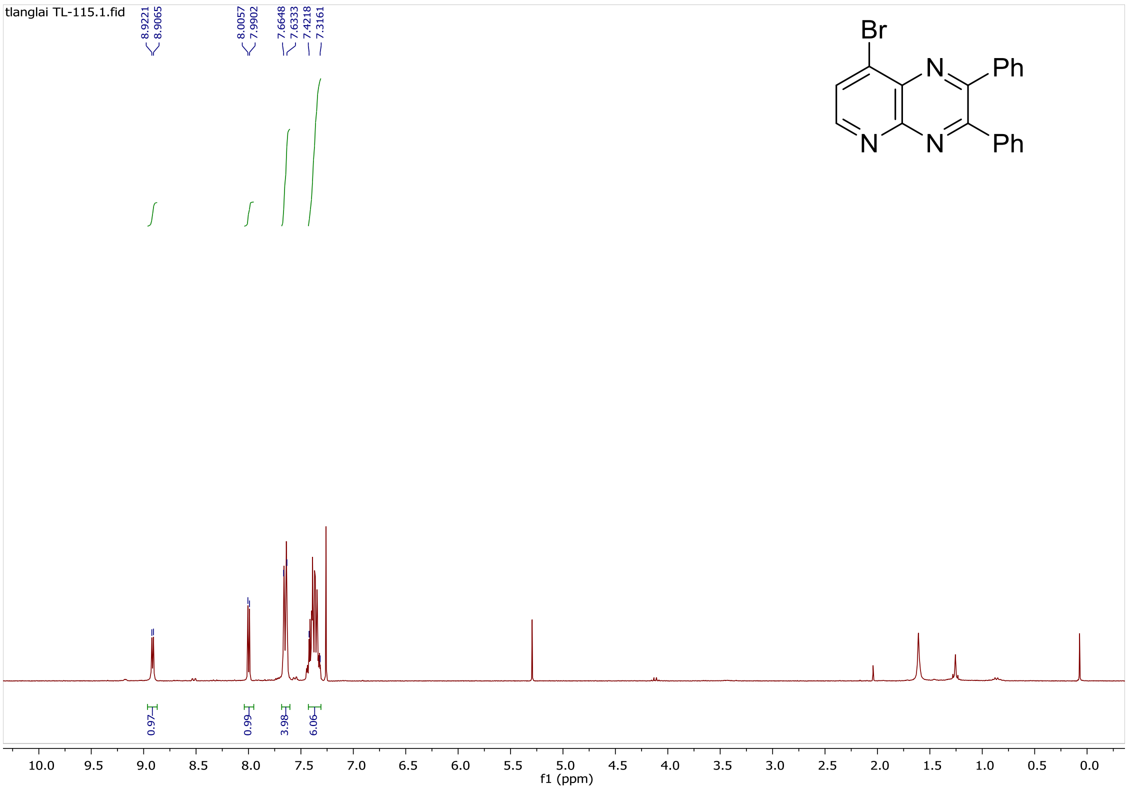

^{13}C NMR (75 MHz, CDCl$_3$)

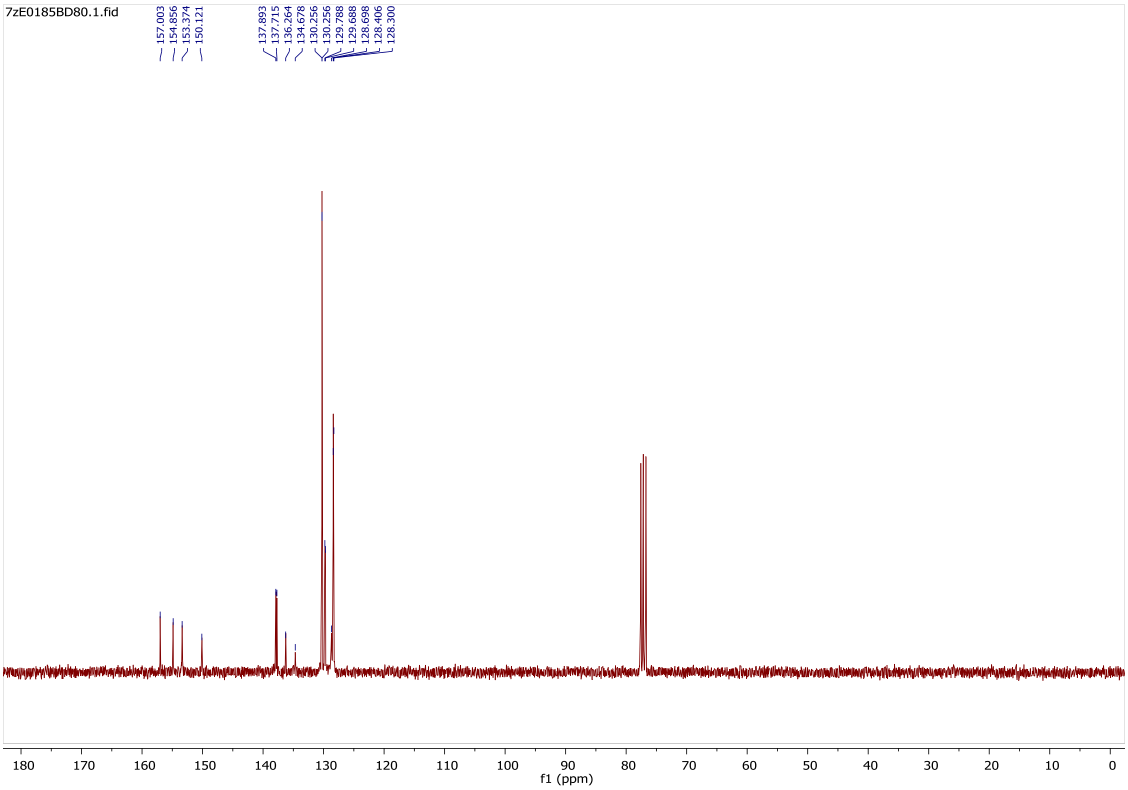

Compound 2b-Cl

^{1}H NMR (300 MHz, $CDCl_3$)

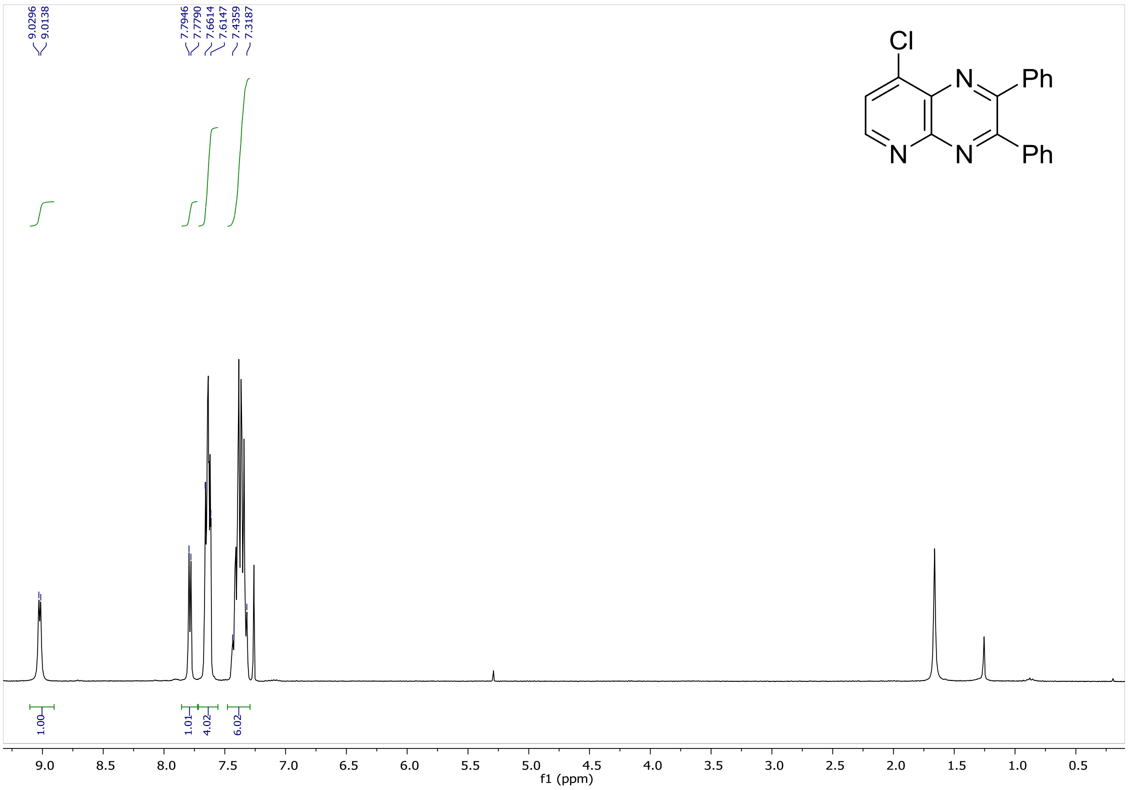

^{13}C NMR (75 MHz, $CDCl_3$)

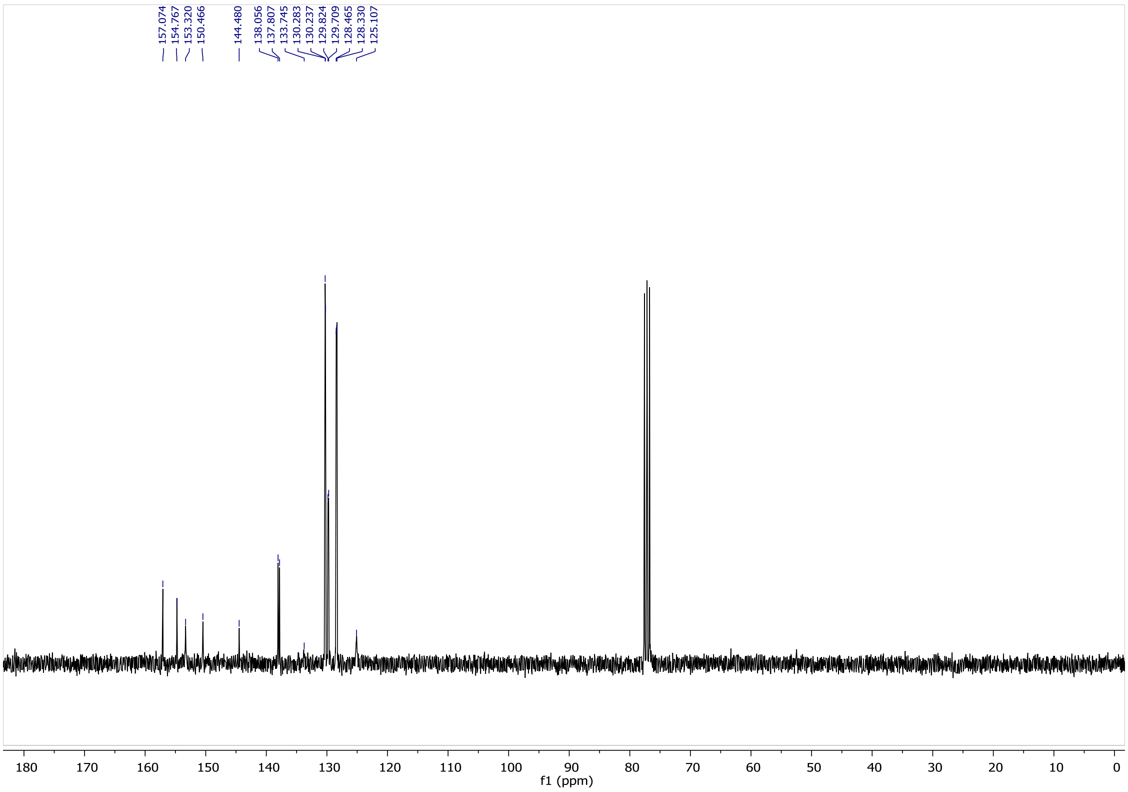

Compound 3b

^{1}H NMR (300 MHz, $CDCl_3$)

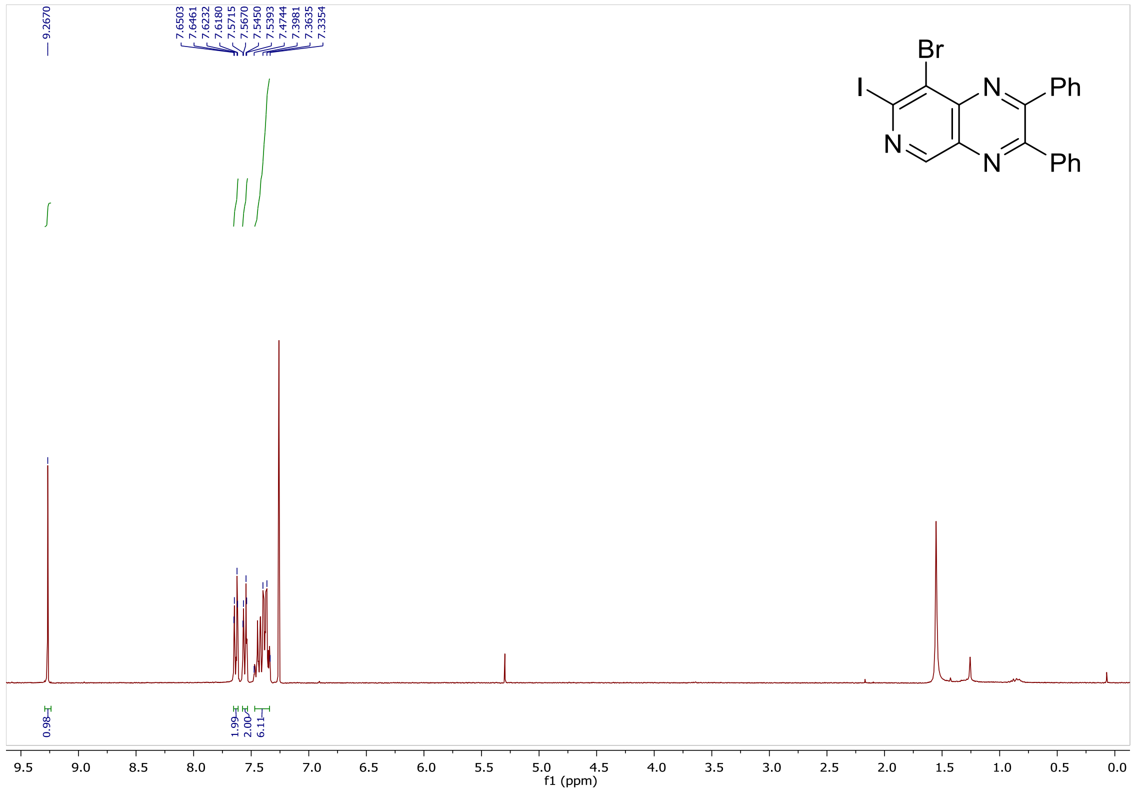

^{13}C NMR (75 MHz, $CDCl_3$)

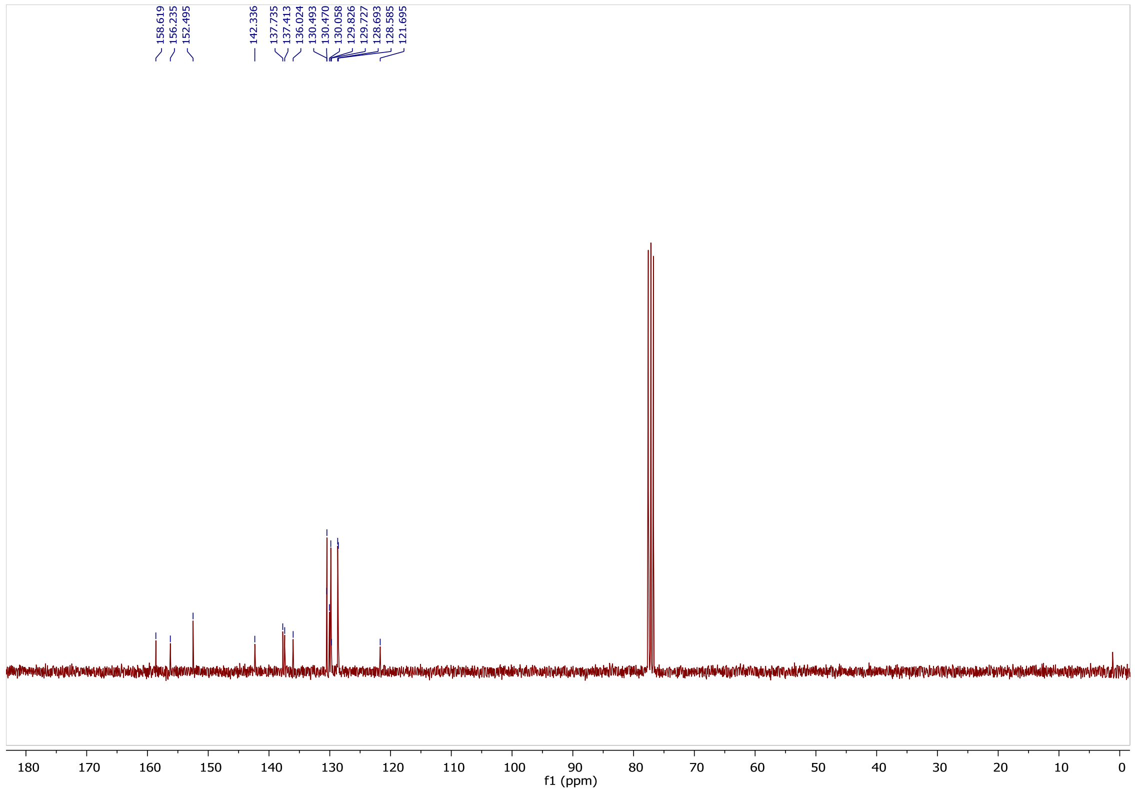

Compound 4a

^{1}H NMR (500 MHz, $CDCl_3$)

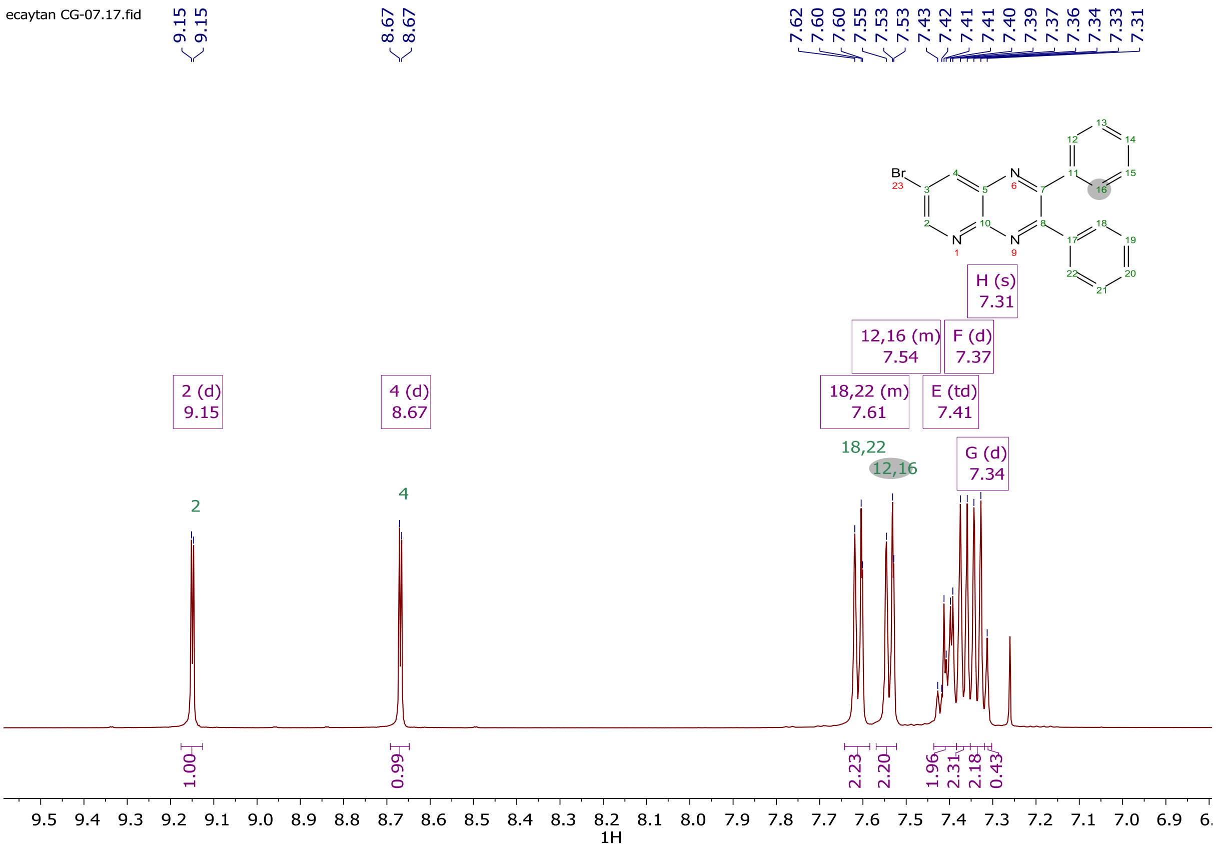

^{13}C NMR (126 MHz, $CDCl_3$)

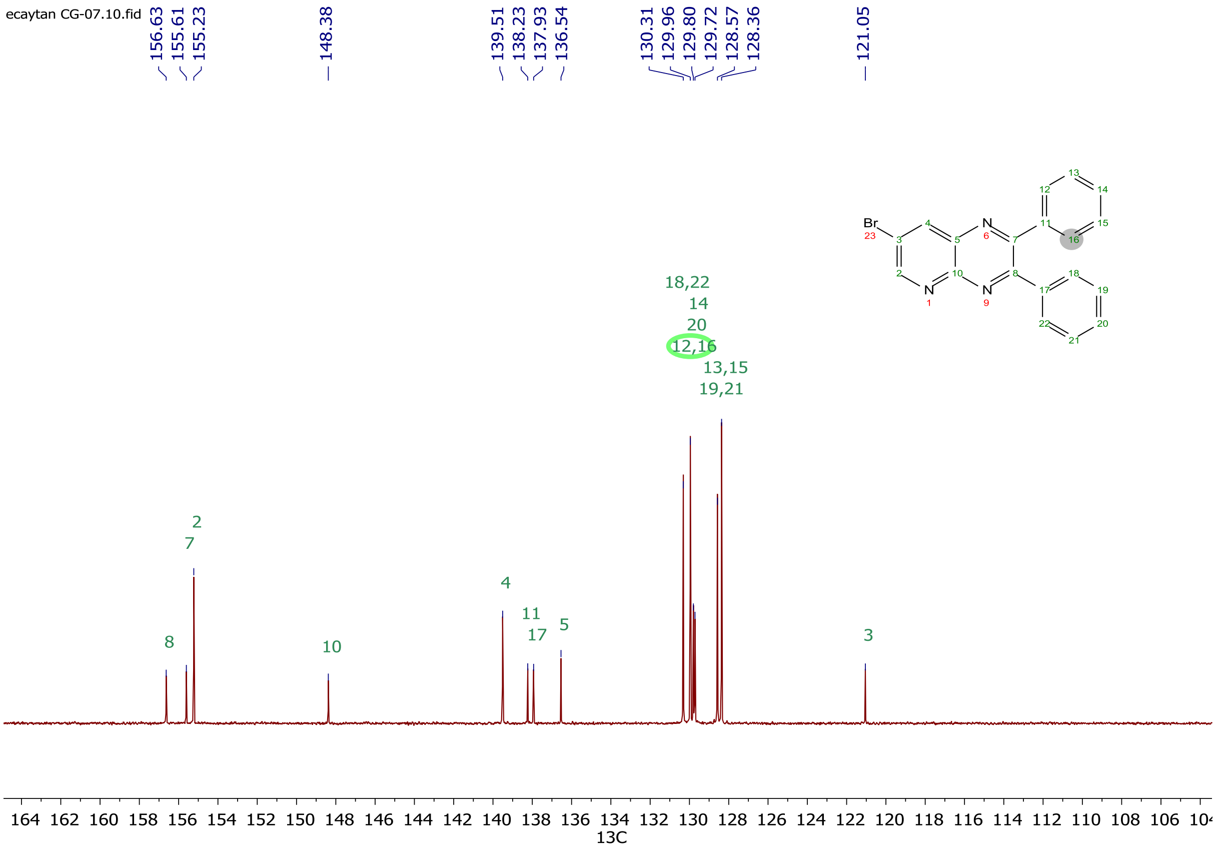

HSQC spectrum (500MHz, 126 MHz, $CDCl_3$)

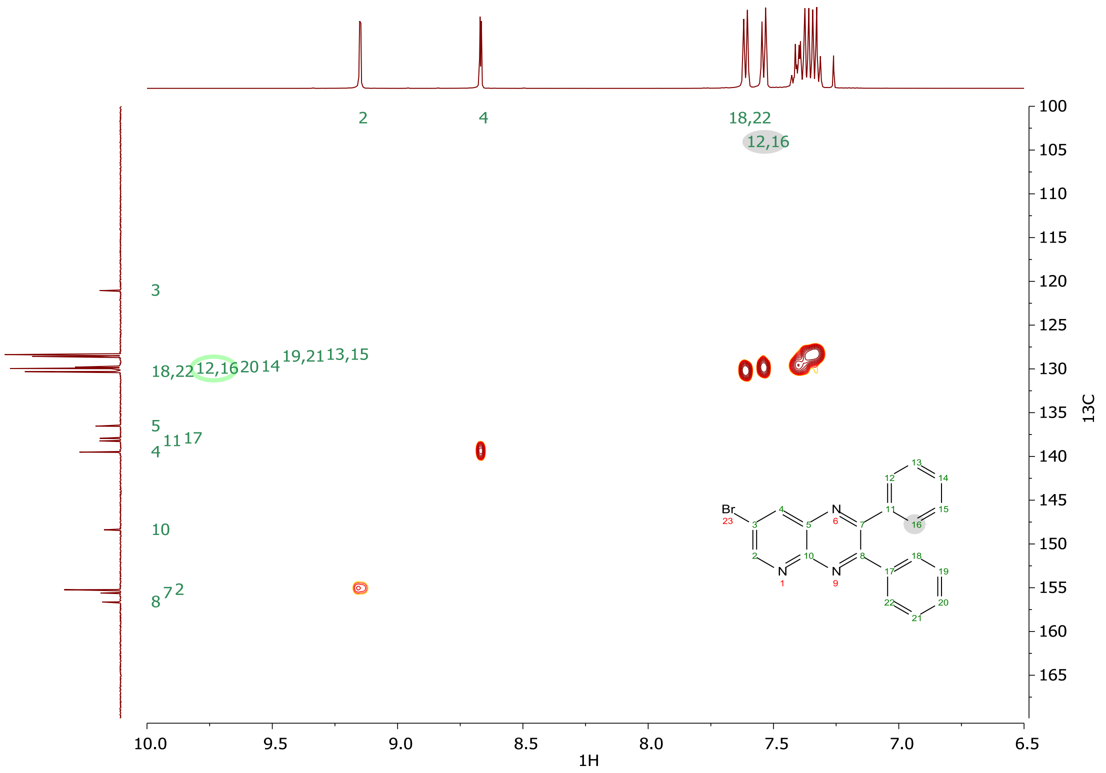

^{13}C-Band-selective HSQC spectrum (500 MHz, 126 MHz, $CDCl_3$)

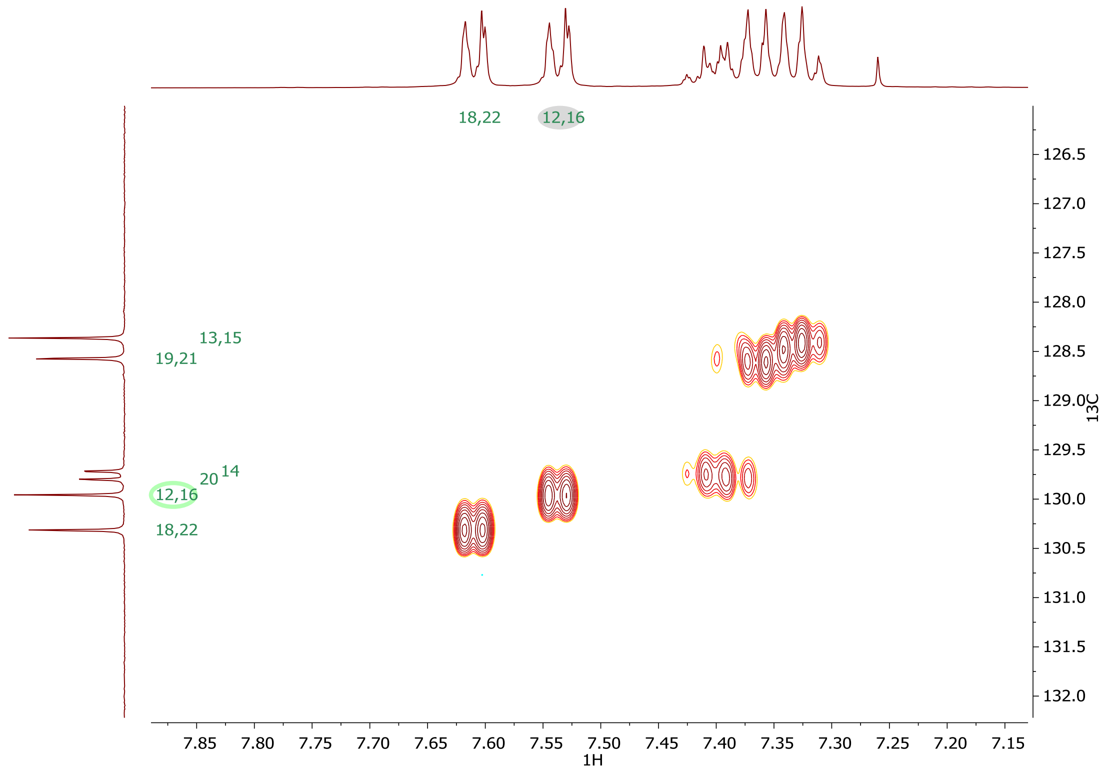

HMBC spectrum (500 MHz, 126 MHz, $CDCl_3$)

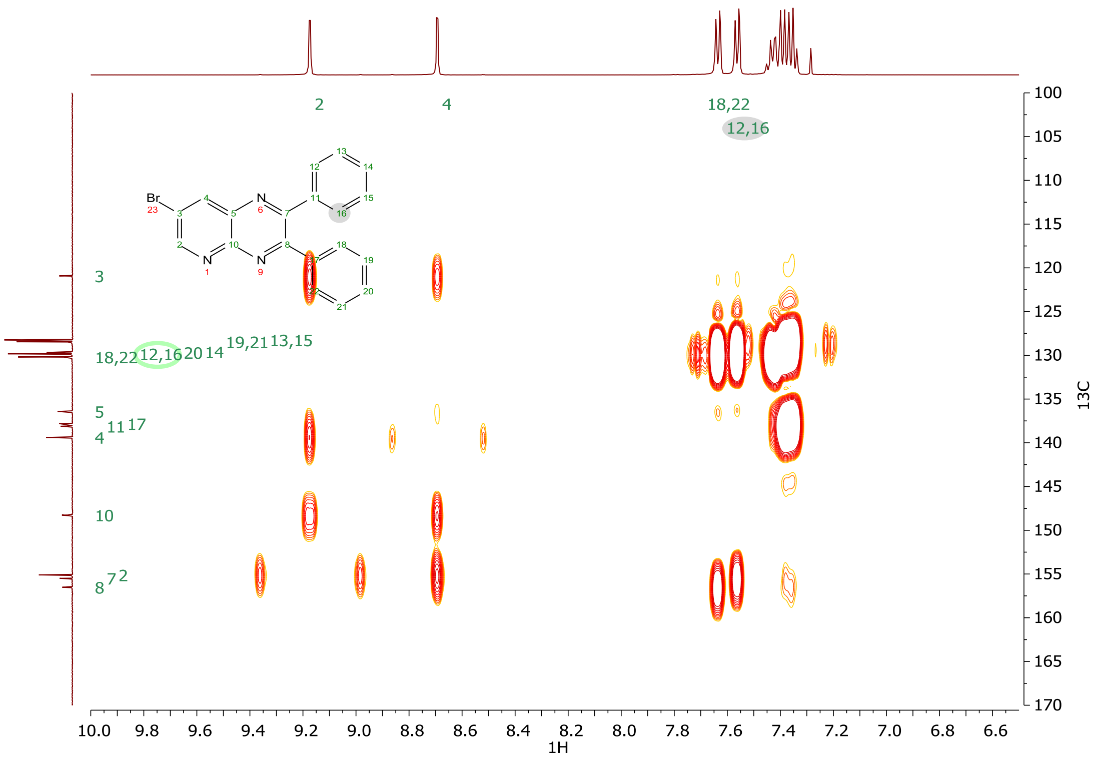

^{13}C-Band-selective HMBC spectrum (500 MHz, 126 MHz, $CDCl_3$)

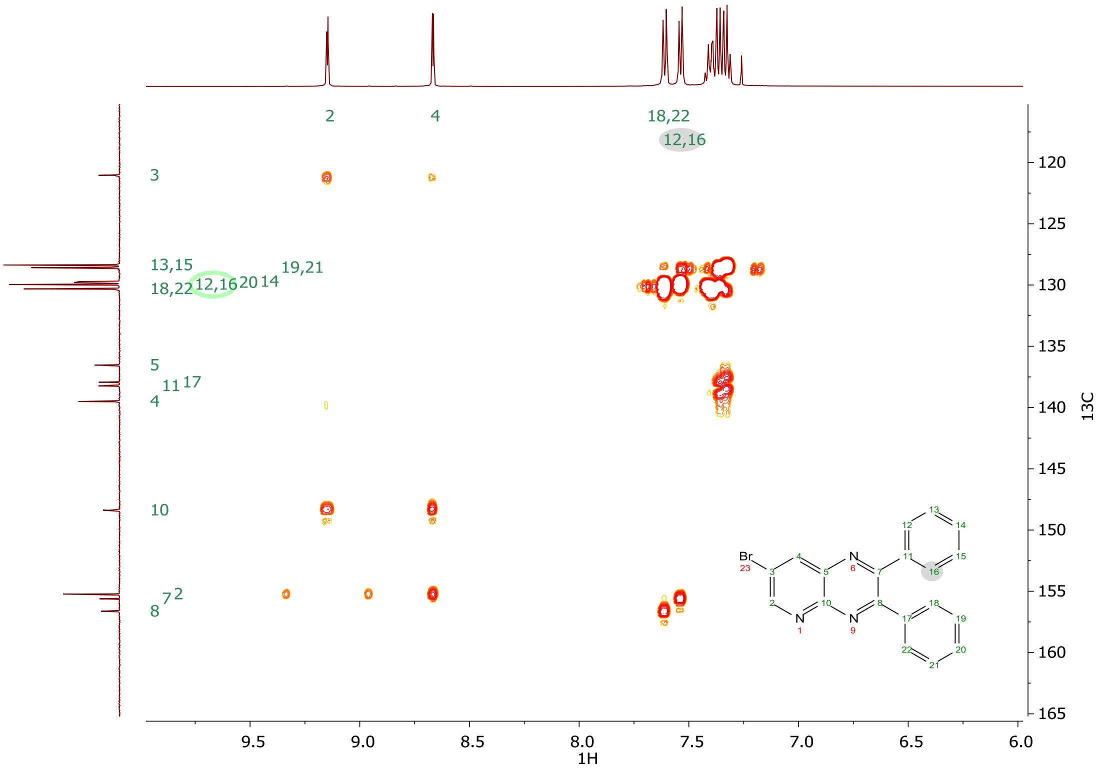

^{1}H-^{15}N HMBC spectrum (500 MHz, 51 MHz, $CDCl_3$)

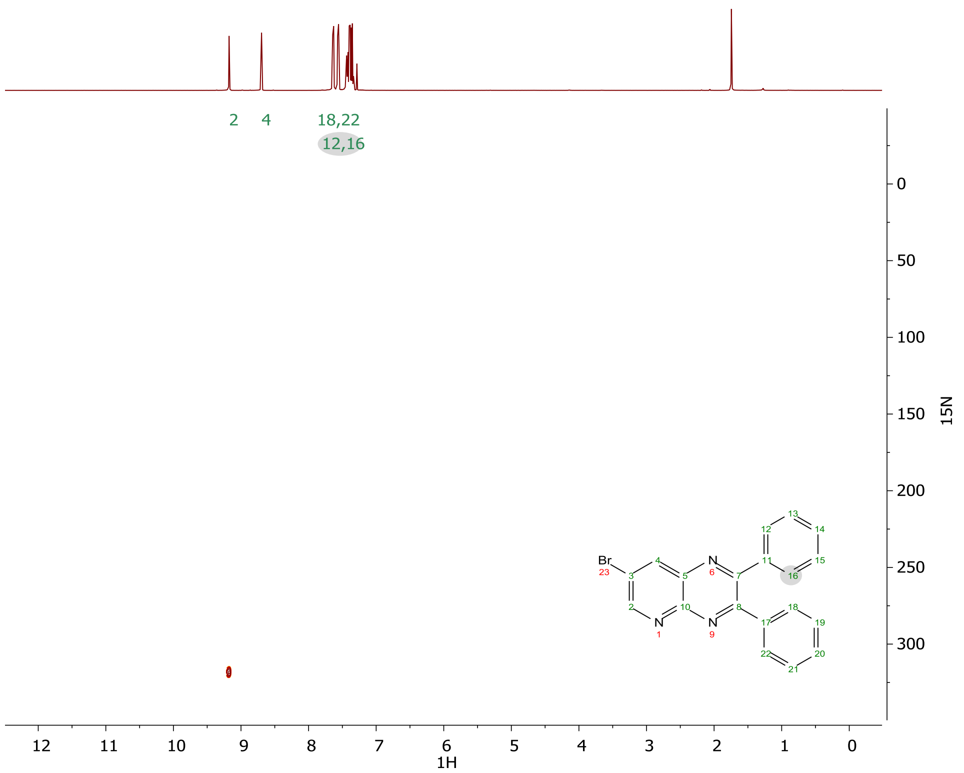

^{1}H NOESY spectrum (500 MHz, $CDCl_3$)

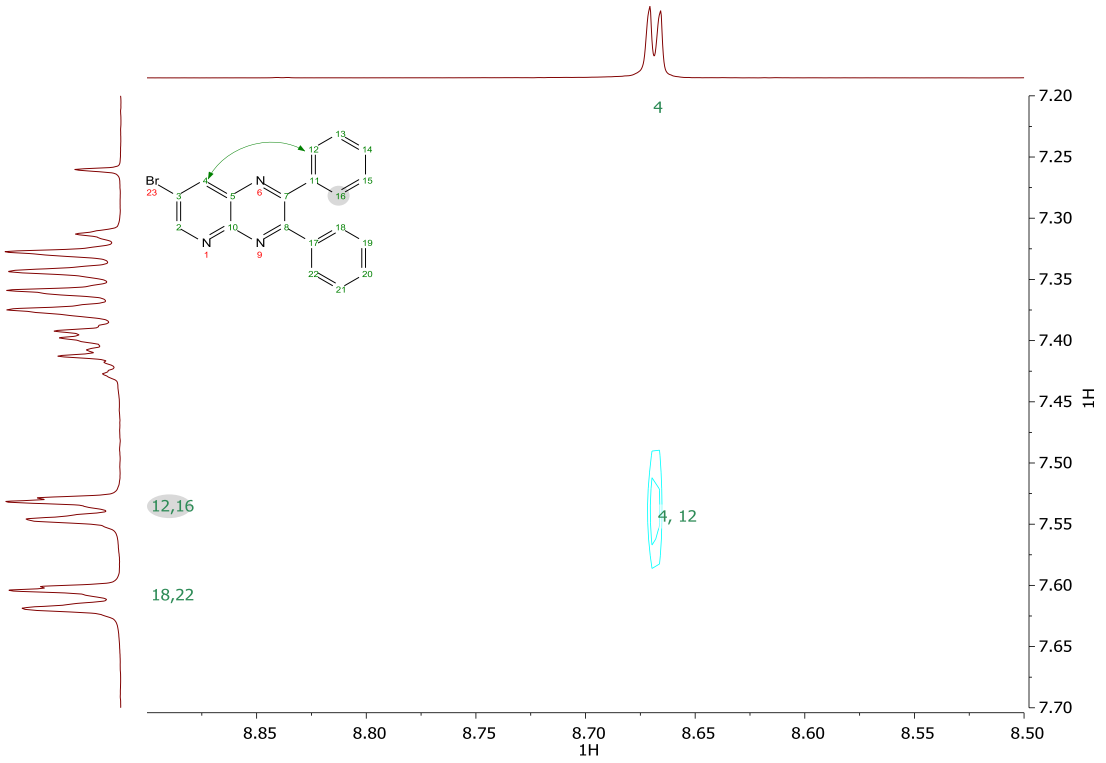

Compound 4b

^{1}H NMR (500 MHz, $CDCl_3$)

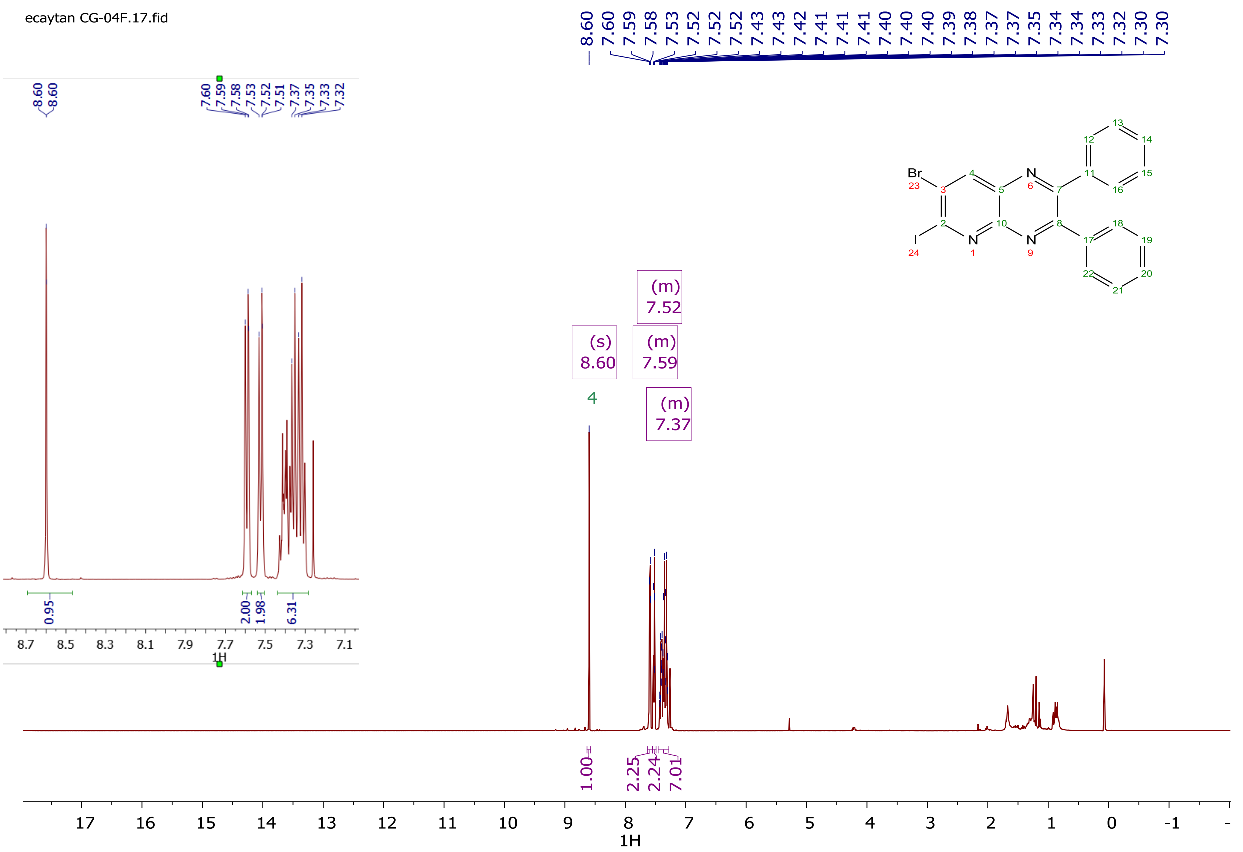

^{13}C NMR (126 MHz, $CDCl_3$)

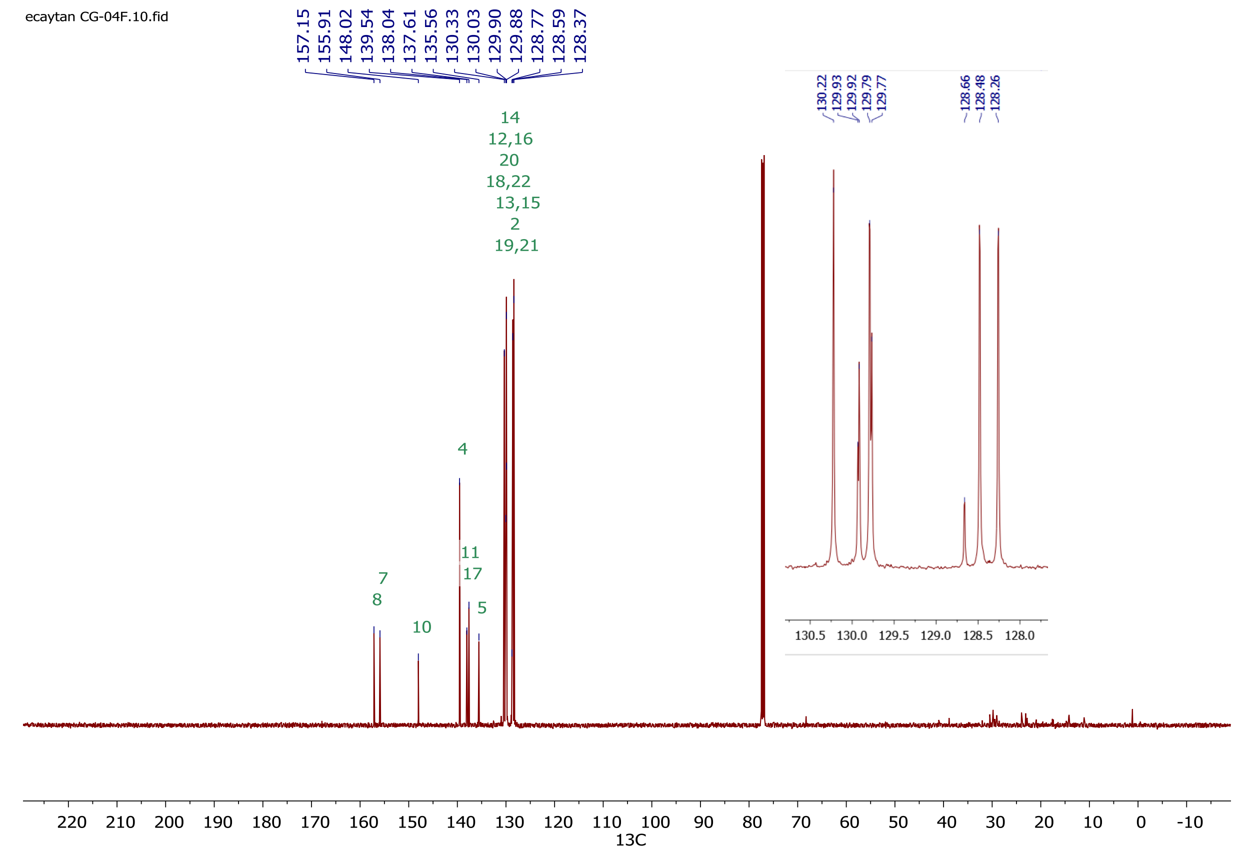

HSQC spectrum (500 MHz, 126 MHz, $CDCl_3$)

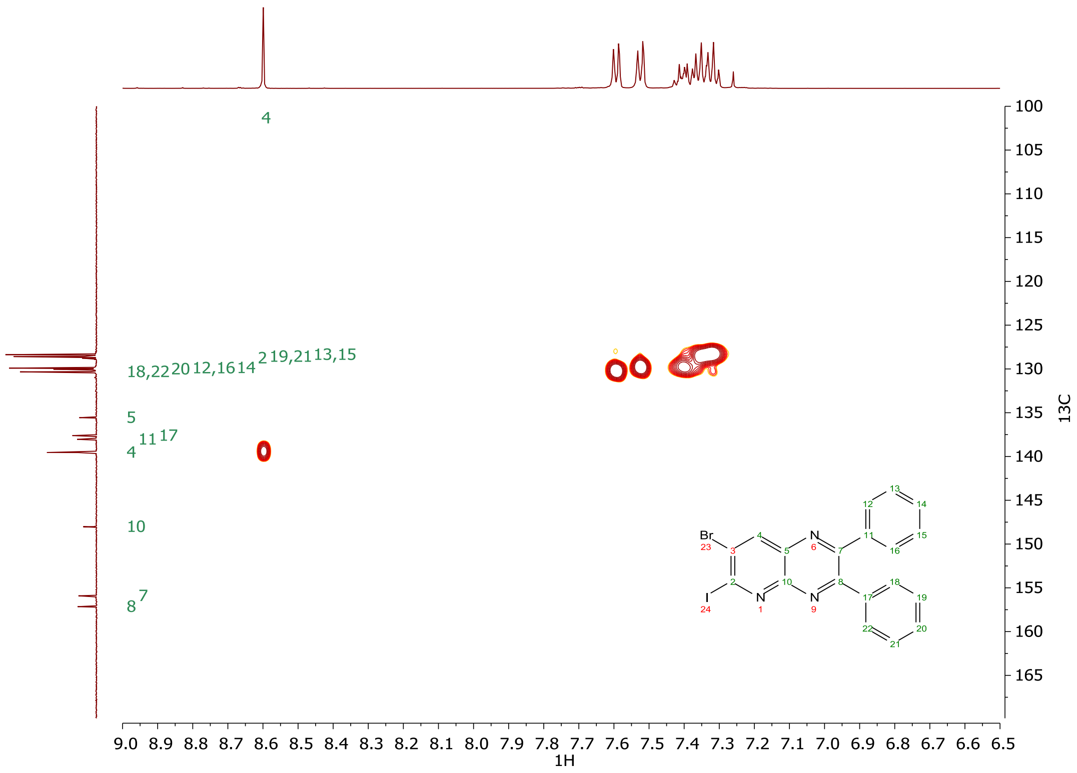

HMBC spectrum (500 MHz, 126 MHz, $CDCl_3$)

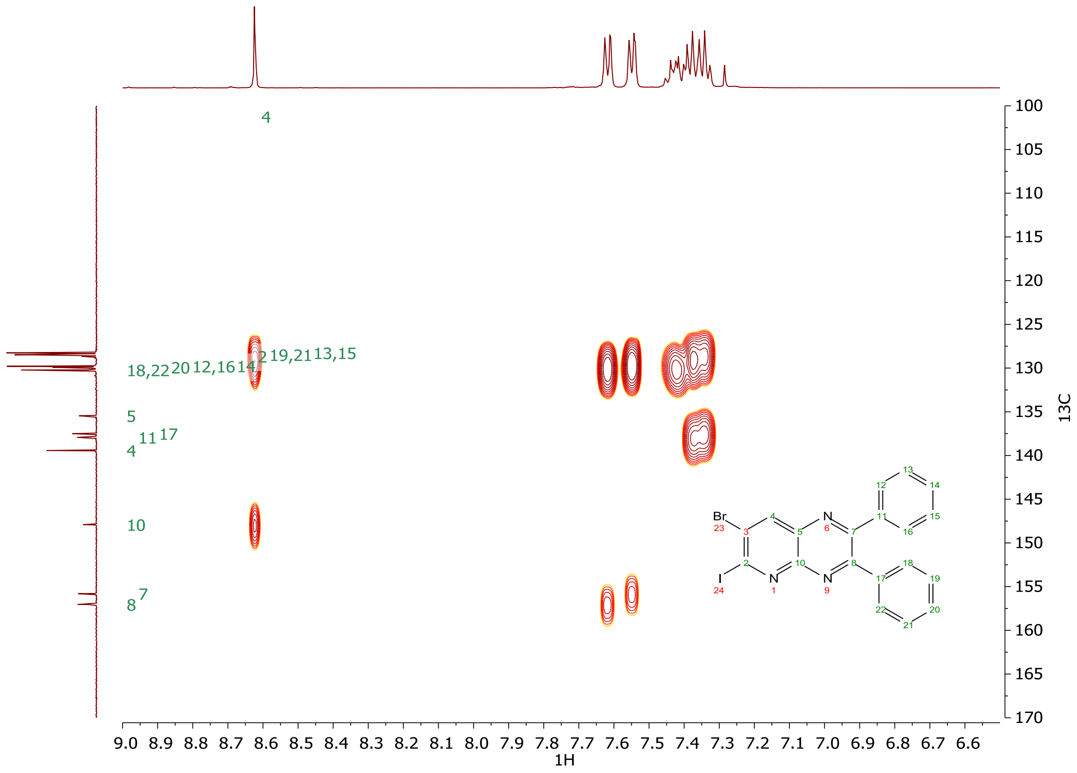

^{1}H NOESY spectrum (500 MHz, $CDCl_3$)

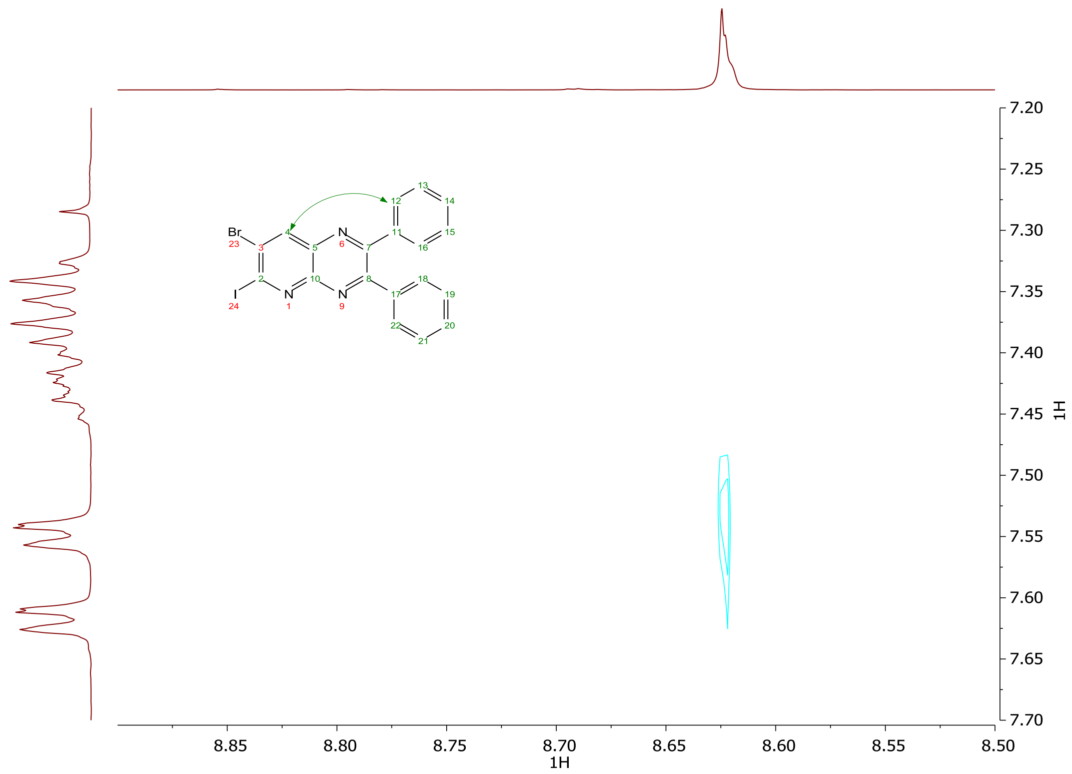

Compound 1c

^{1}H NMR (300 MHz, $CDCl_3$)

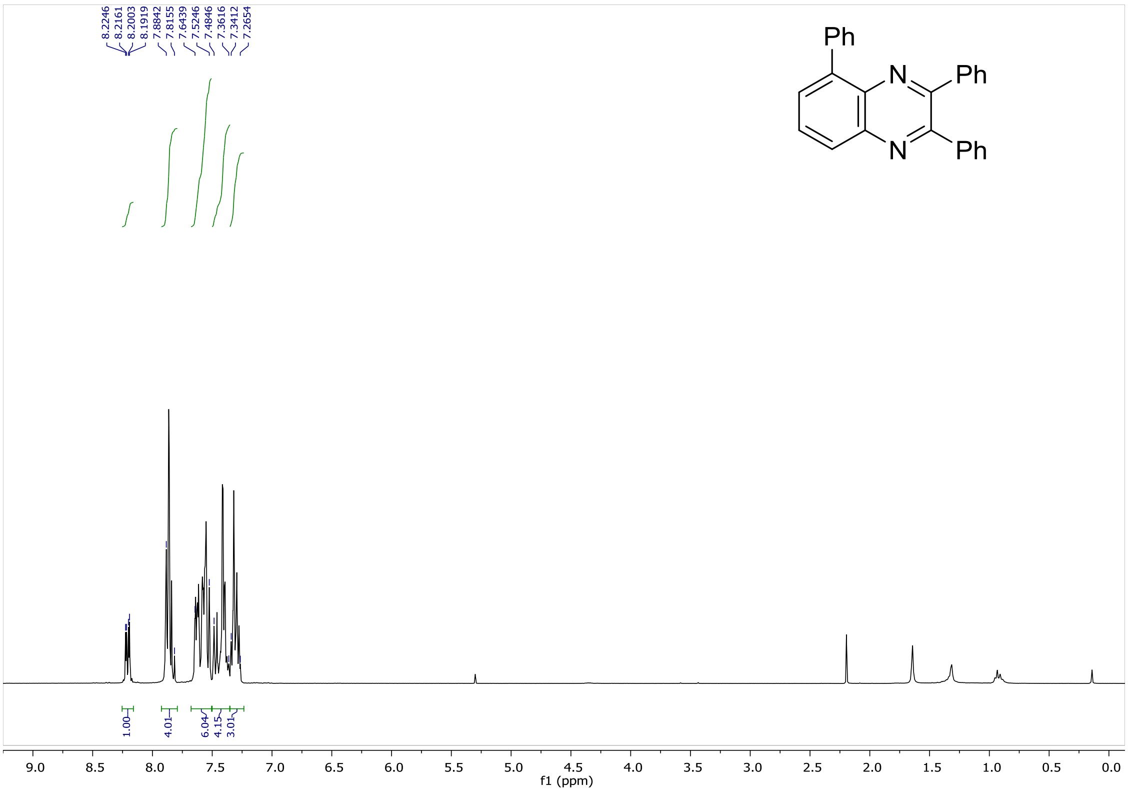

^{13}C NMR (75 MHz, $CDCl_3$)

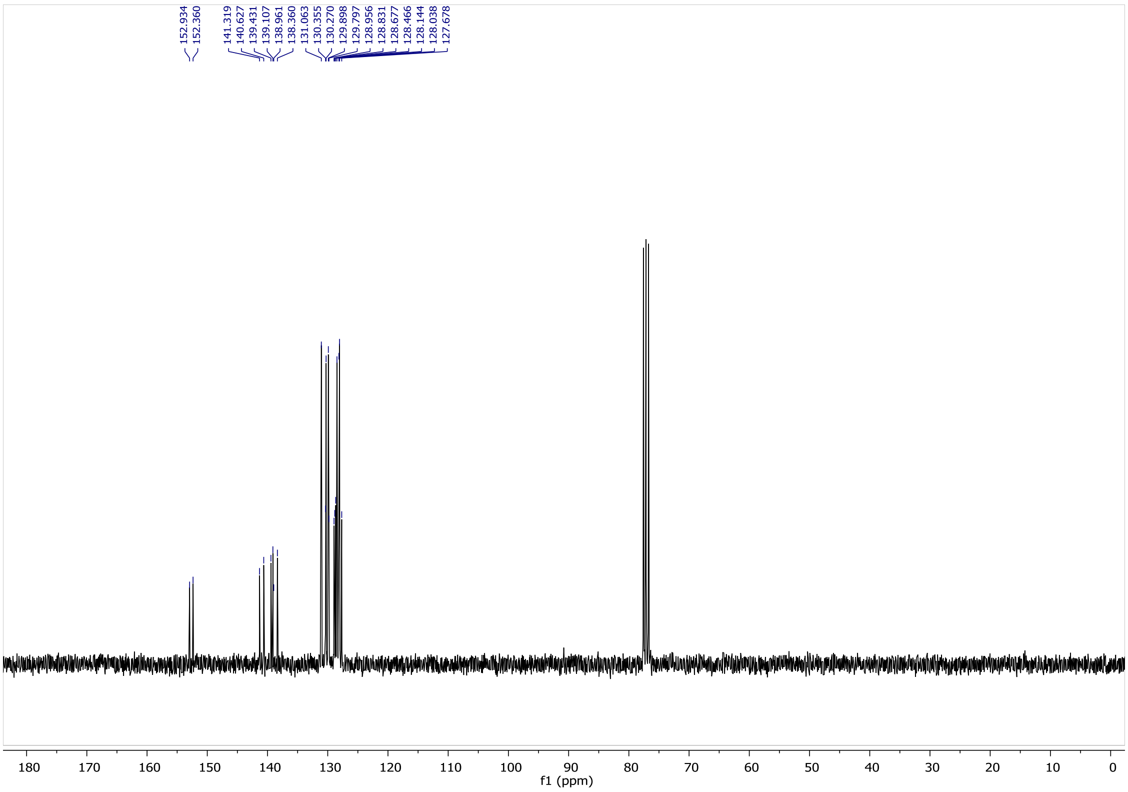

Compound 1d

^{1}H NMR (500 MHz, $CDCl_3$)

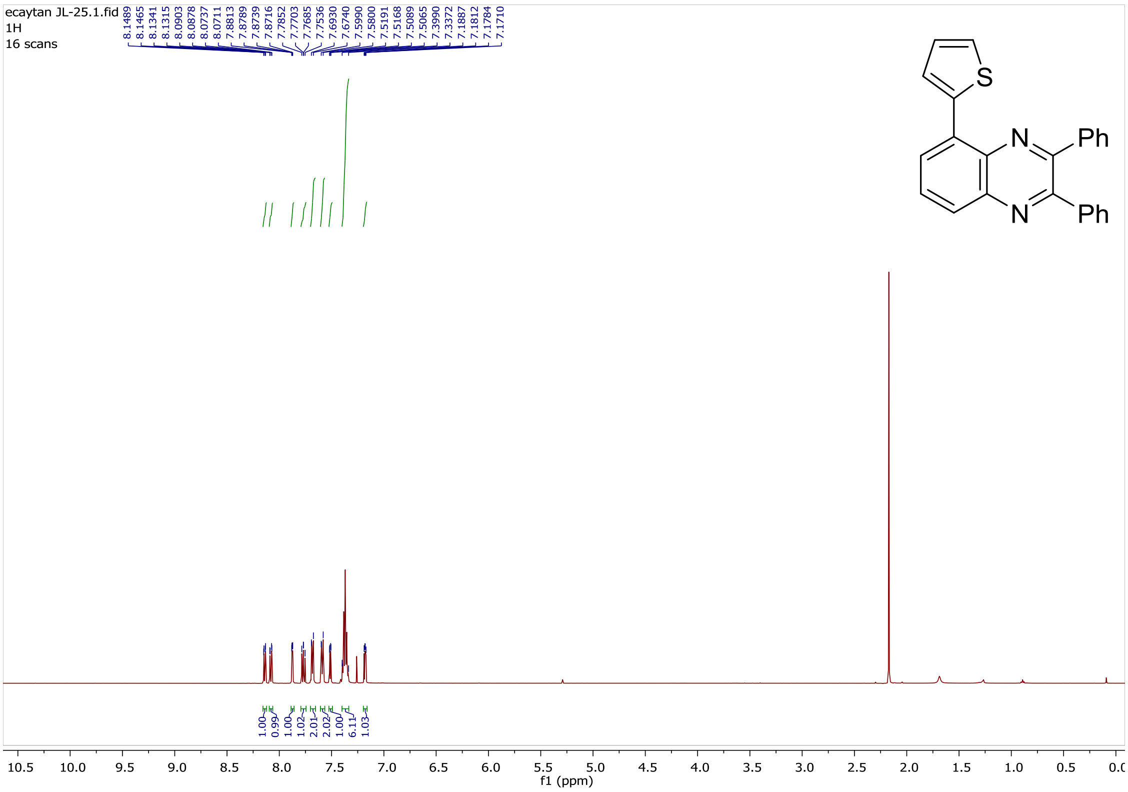

^{13}C NMR (126 MHz, $CDCl_3$)

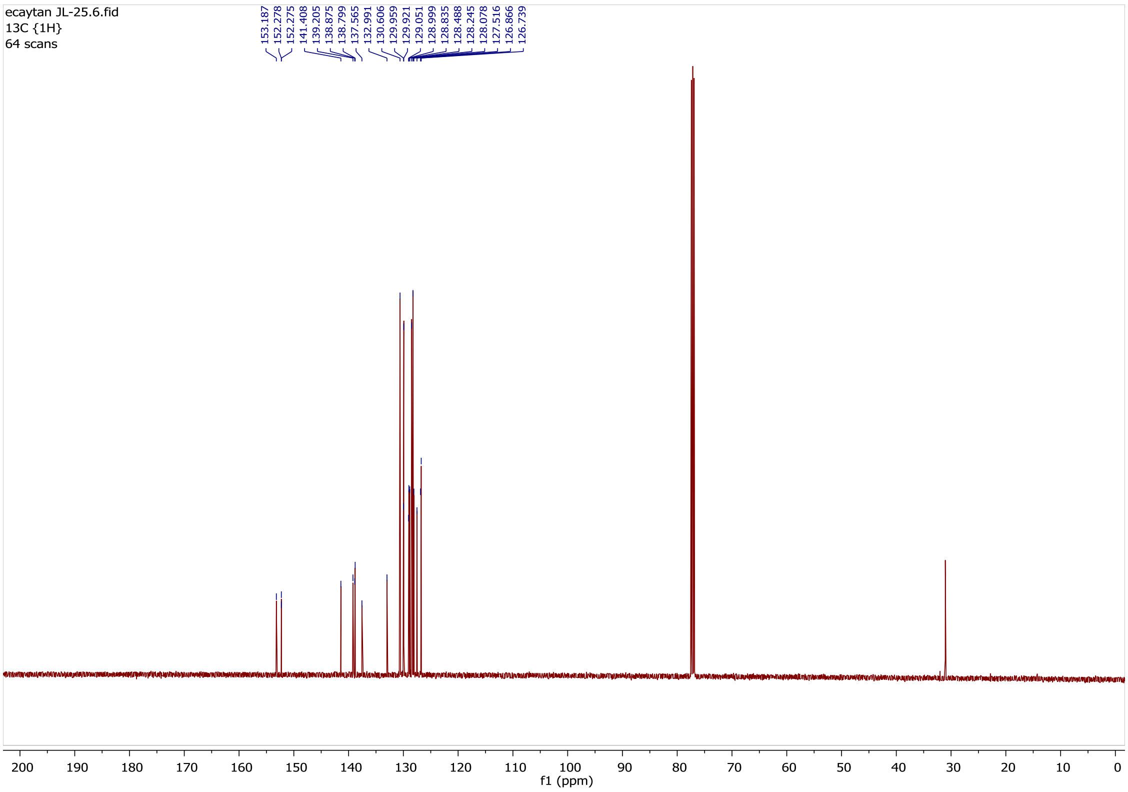

^{13}C-Band-selective HSQC spectrum (500 MHz, 126 MHz, $CDCl_3$)

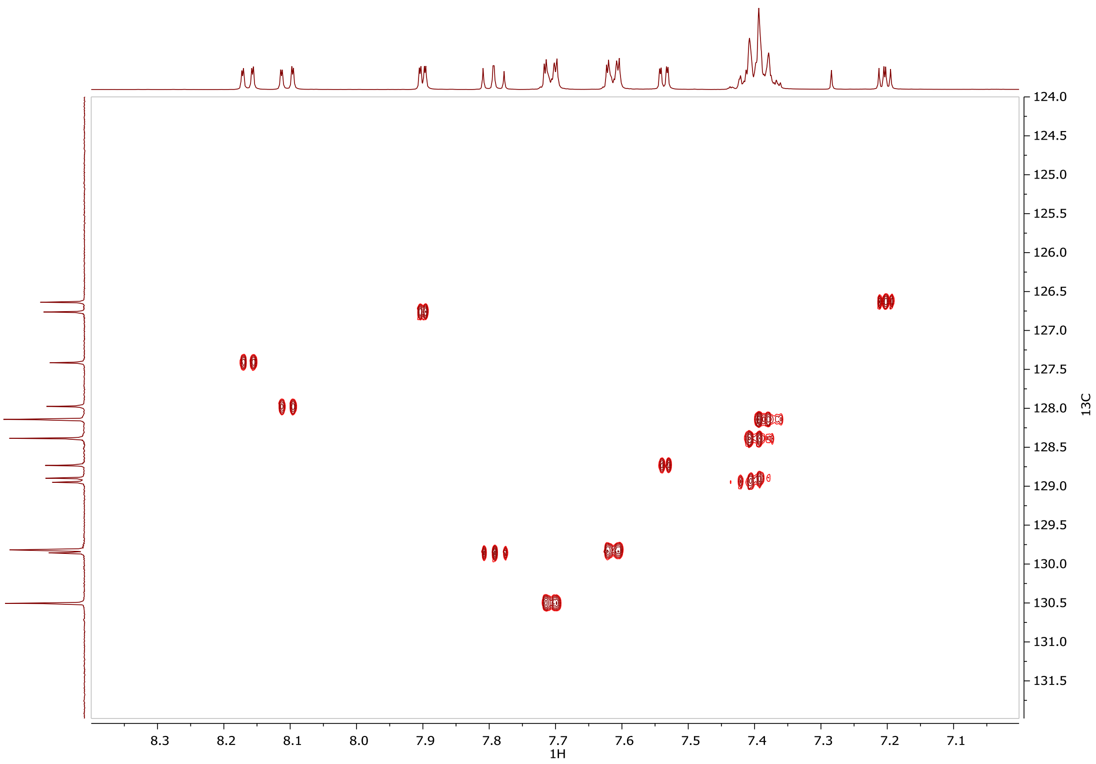

^{13}C-Band-selective HMBC spectra (500 MHz, 126 MHz, $CDCl_3$)

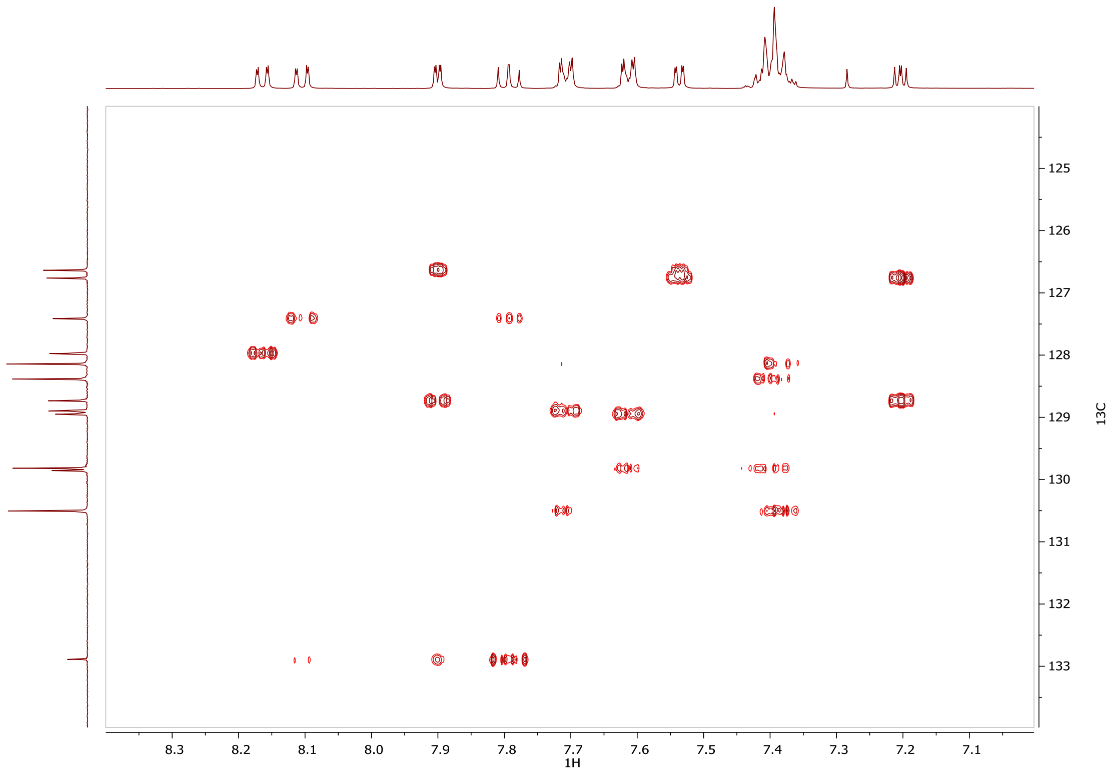

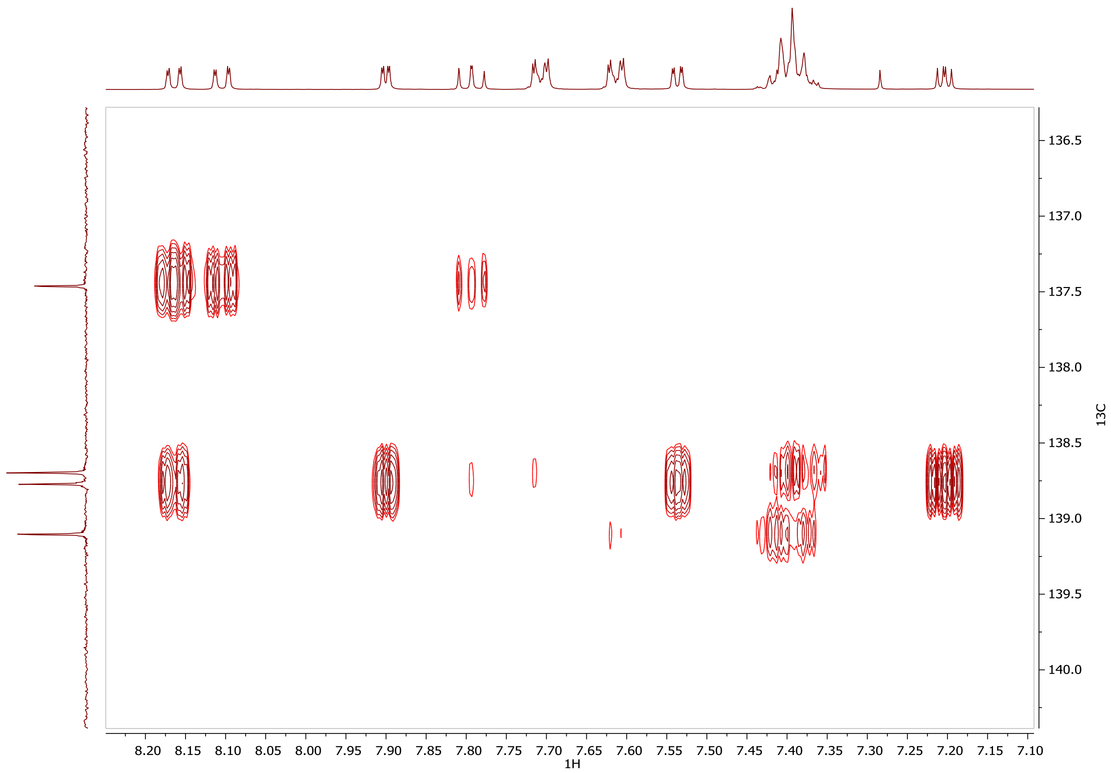

Compound 2d

^{1}H NMR (300 MHz, CDCl$_3$)

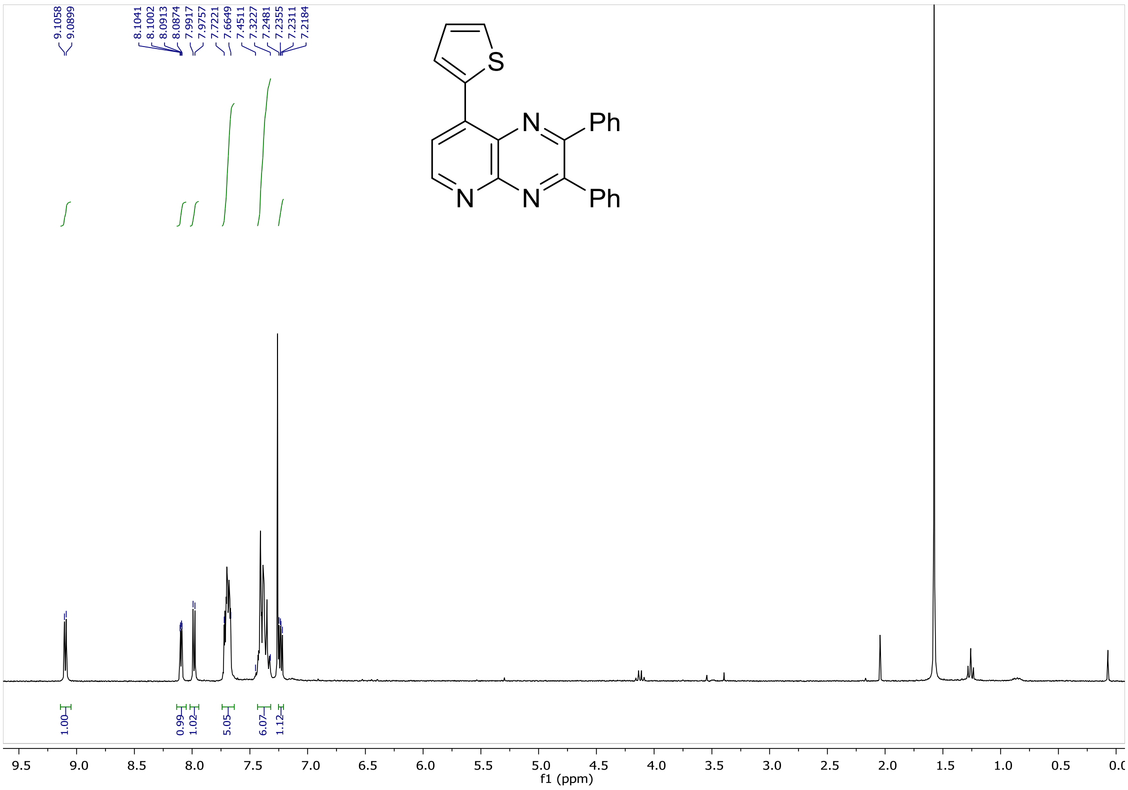

^{13}C NMR (75 MHz, CDCl$_3$)

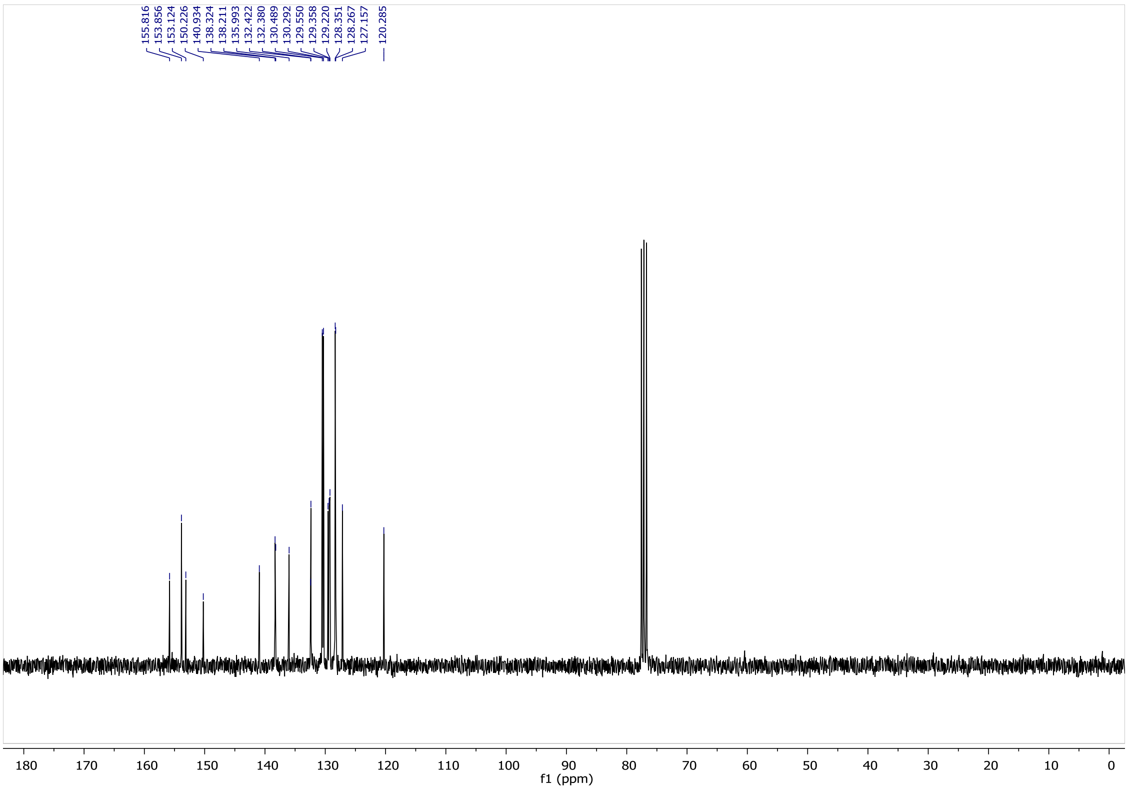

Compound 1e

^{1}H NMR (300 MHz, $CDCl_3$)

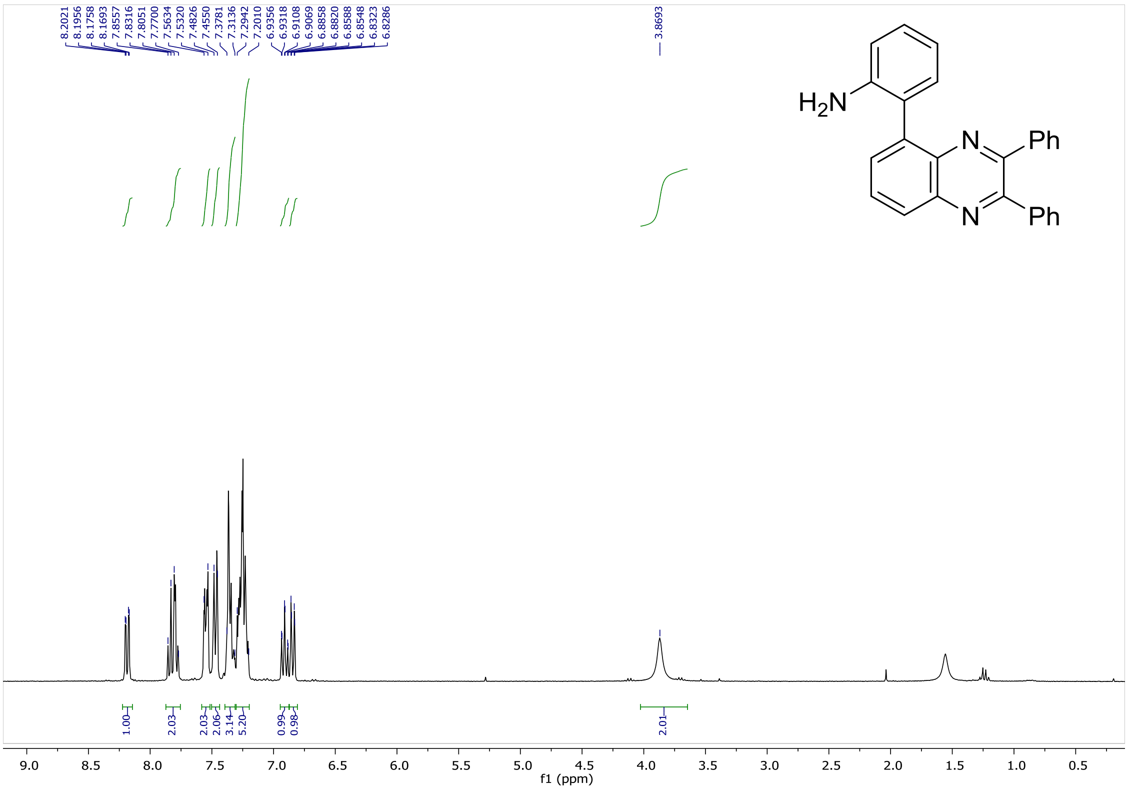

^{13}C NMR (75 MHz, $CDCl_3$)

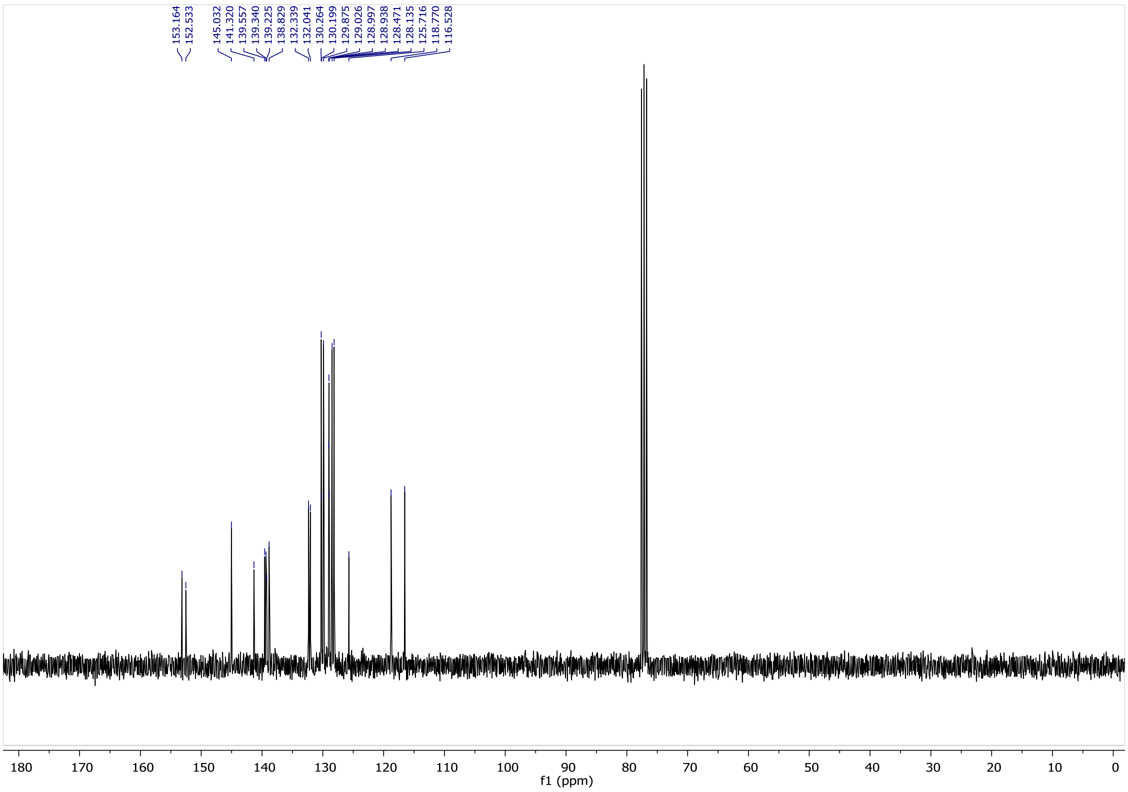

Compound 2e

^{1}H NMR (300 MHz, $CDCl_3$)

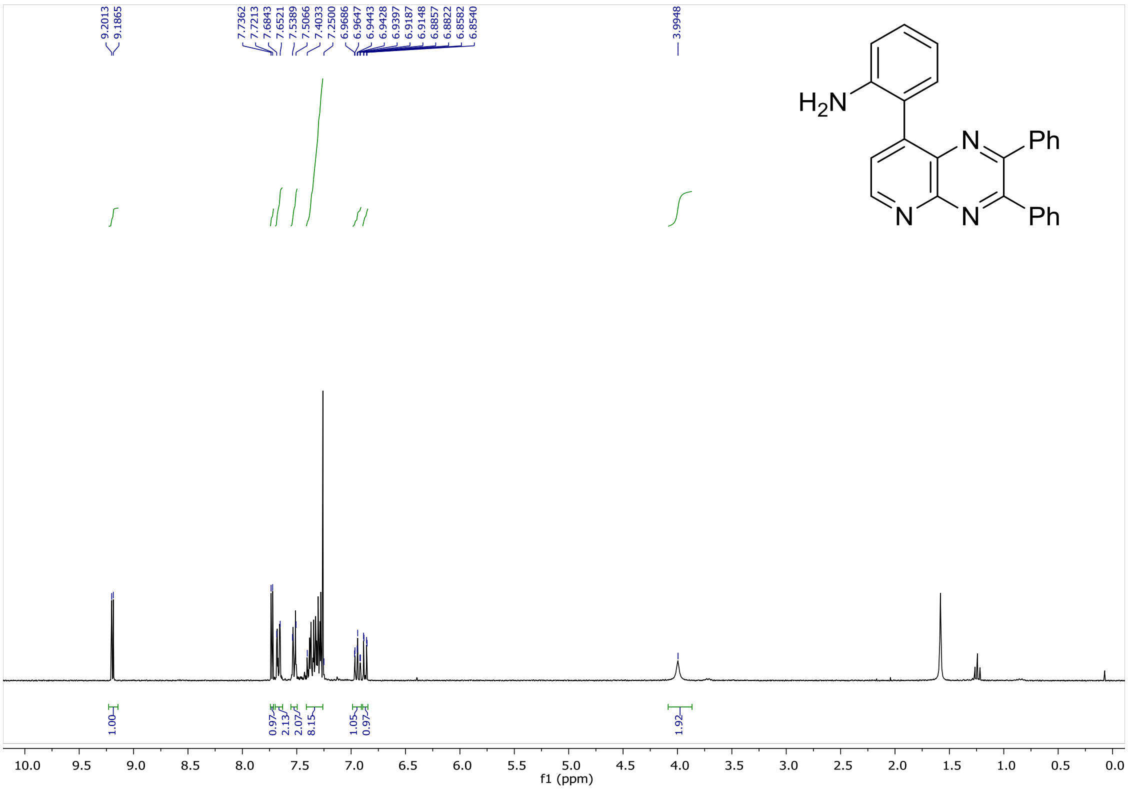

^{13}C NMR (75 MHz, $CDCl_3$)

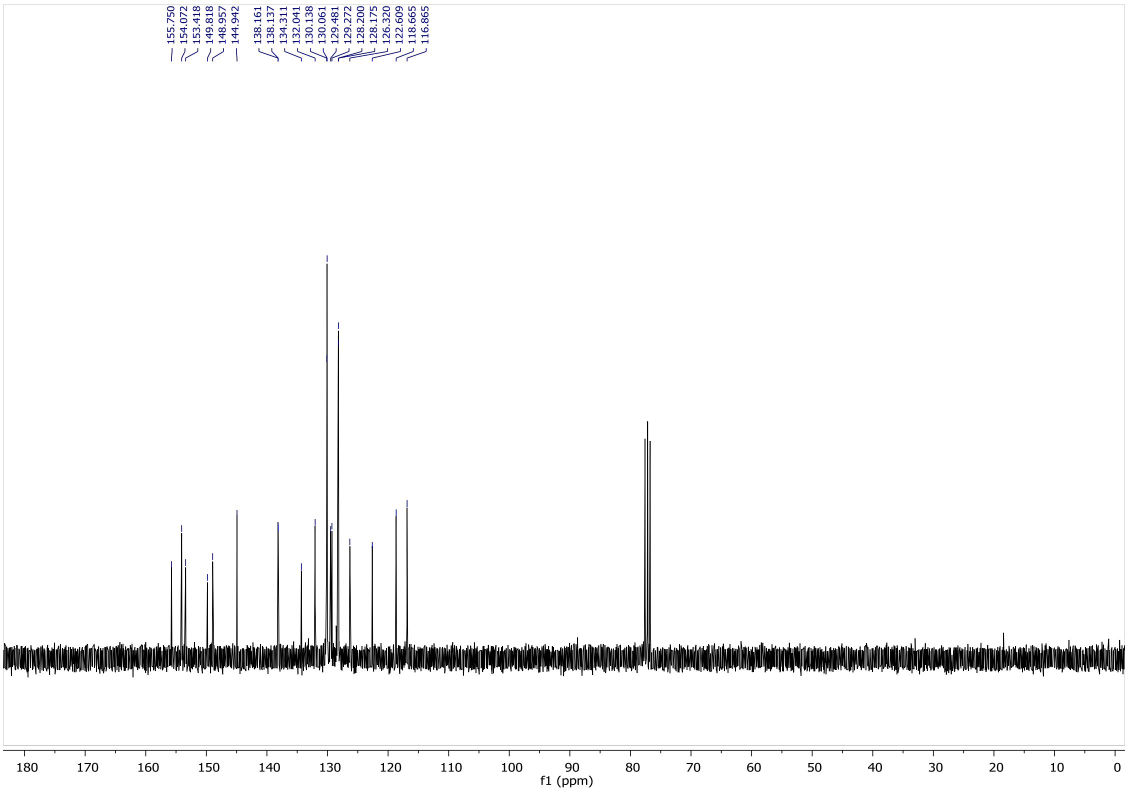

Compound 1f

^{1}H NMR (300 MHz, $CDCl_3$)

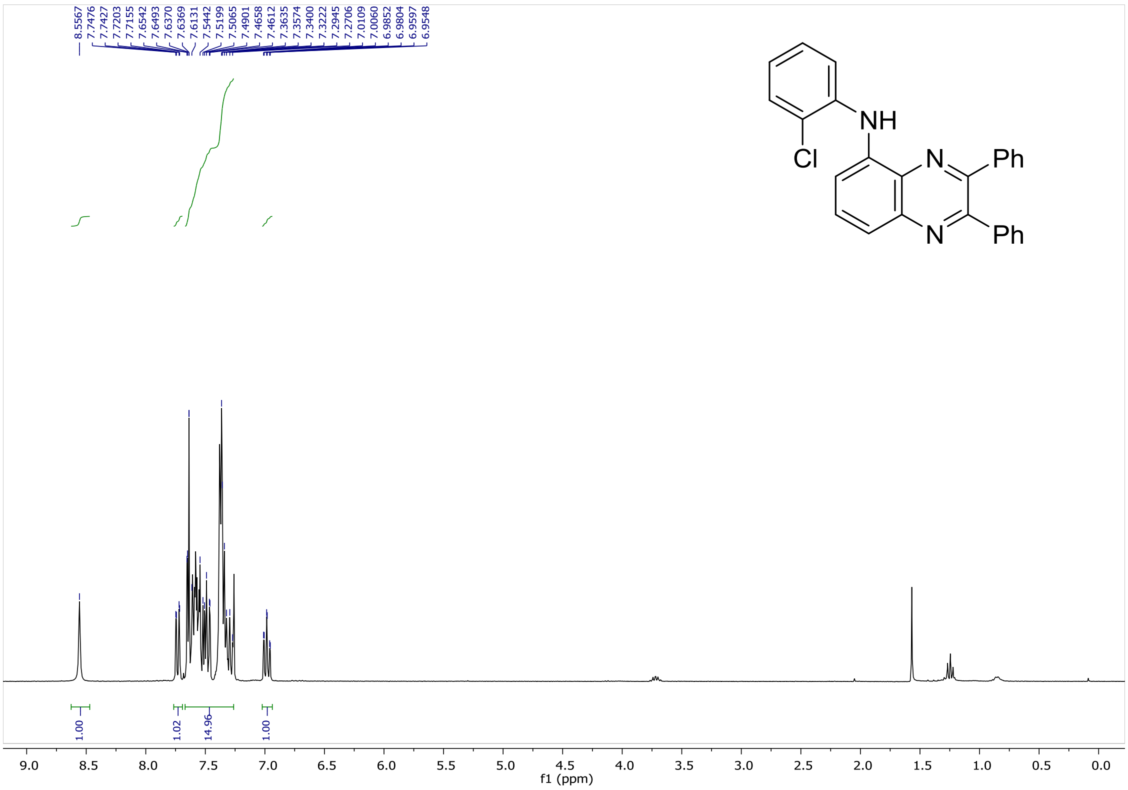

^{13}C NMR (75 MHz, $CDCl_3$)

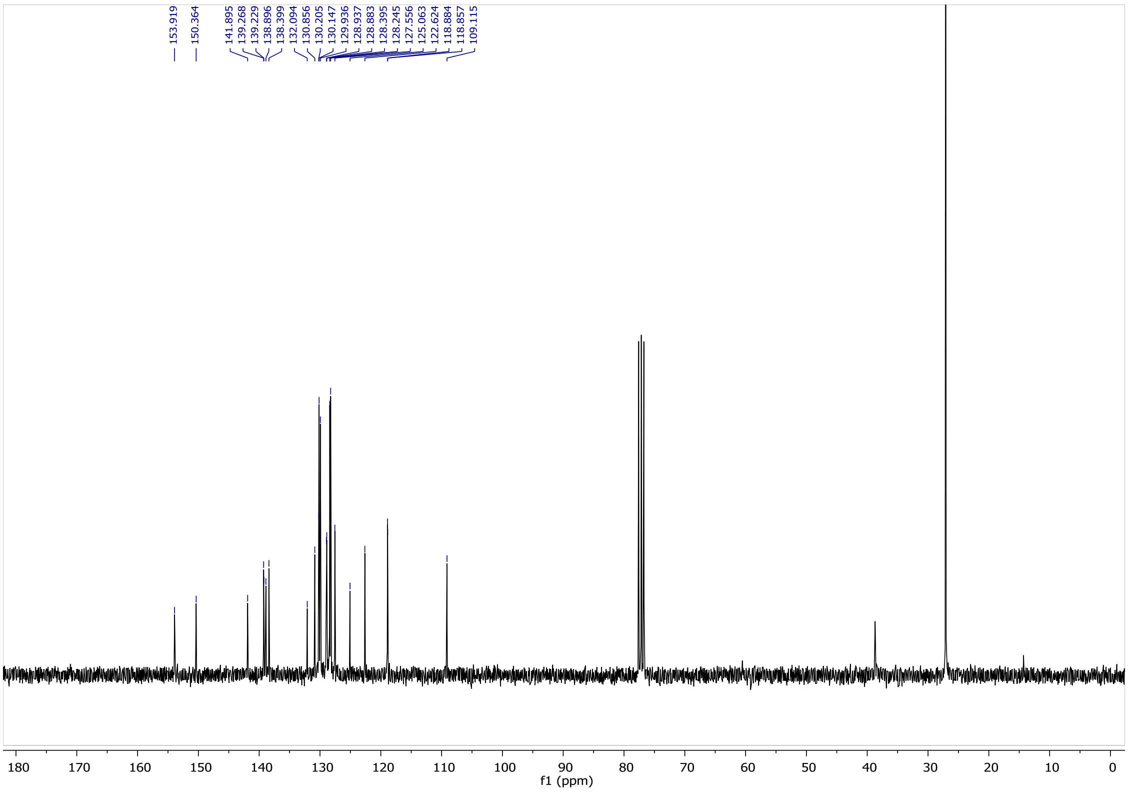

Compound 2f

^{1}H NMR (300 MHz, $CDCl_3$)

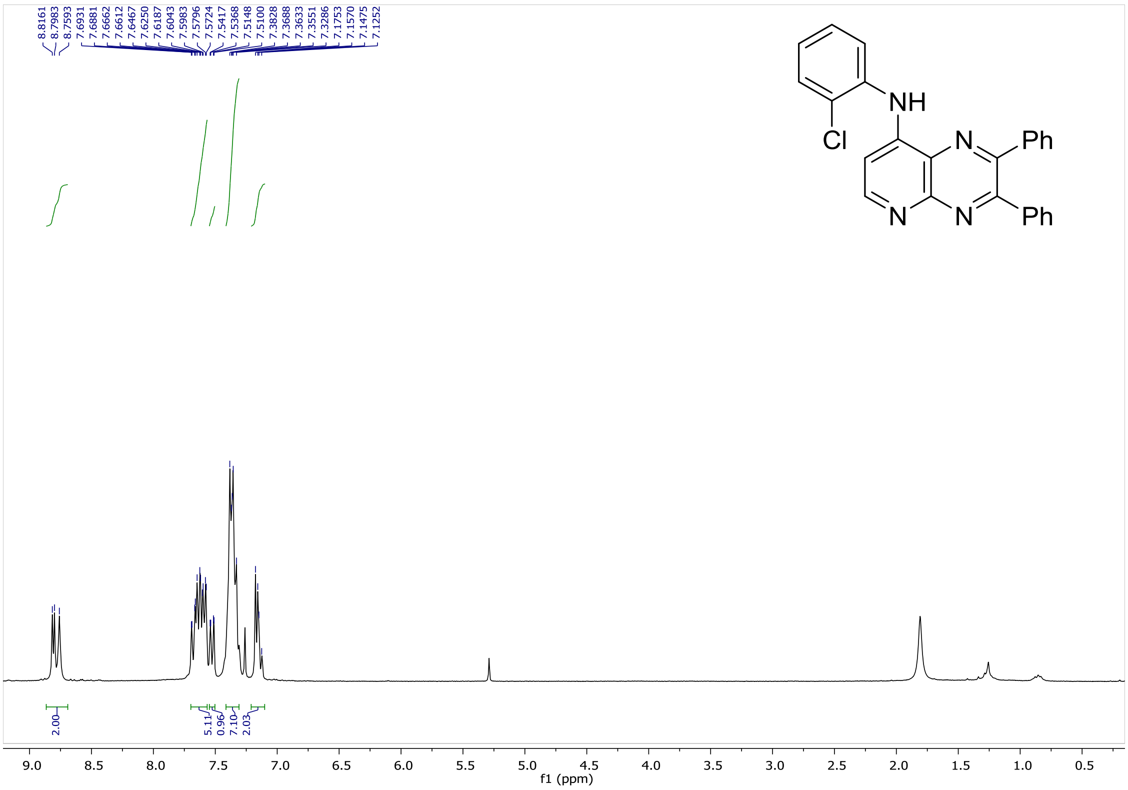

^{13}C NMR (75 MHz, $CDCl_3$)

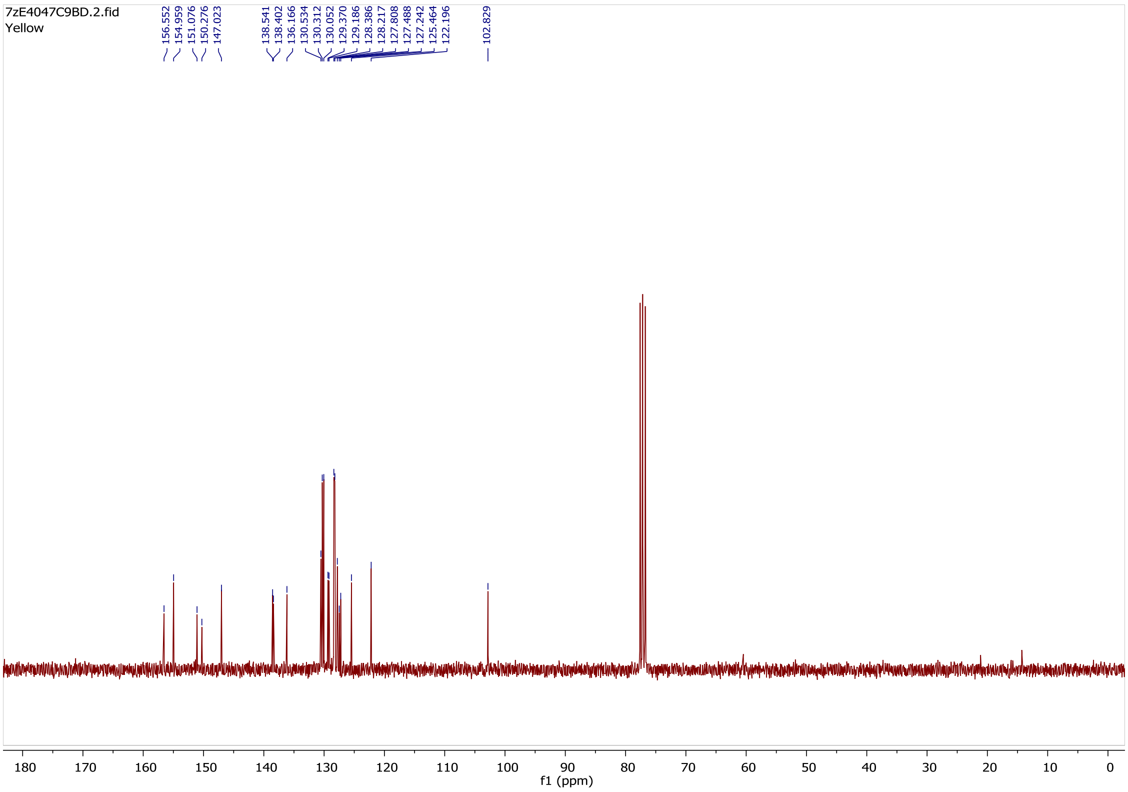

Compound 1g

^{1}H NMR (300 MHz, $(CD_3)_2SO$)

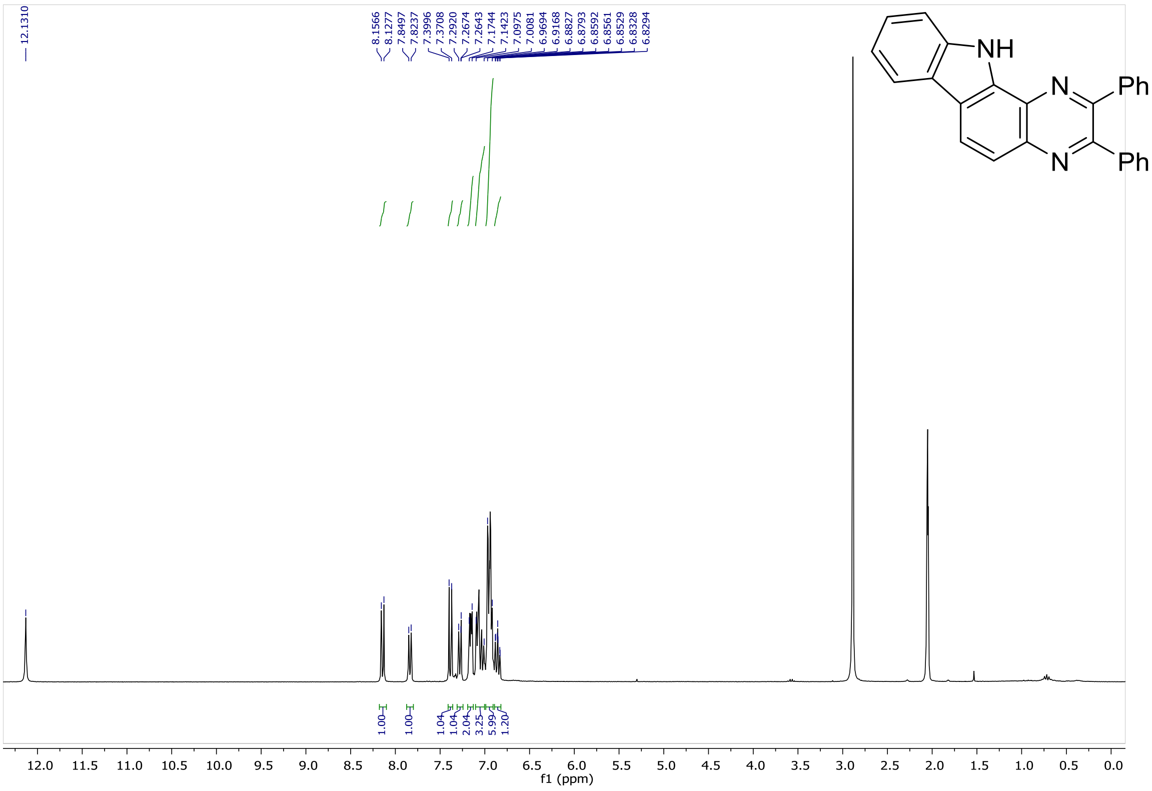

^{13}C NMR (75 MHz, $(CD_3)_2SO$)

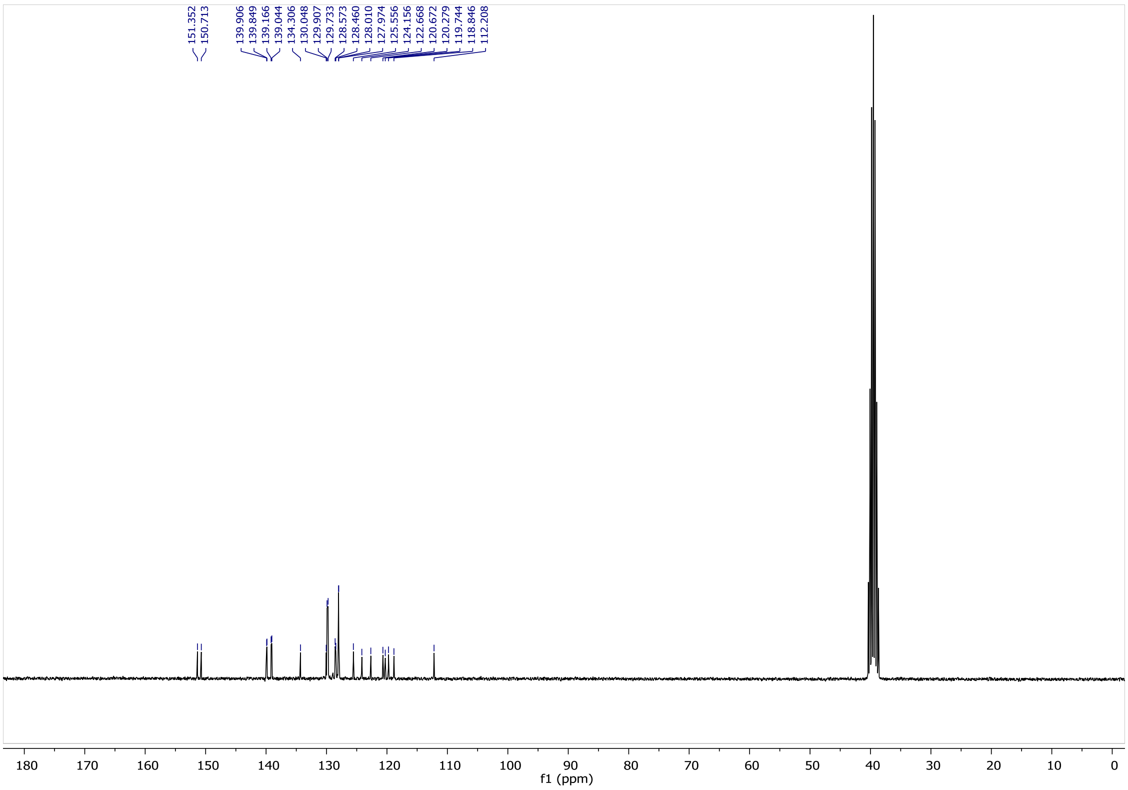

Compound 2g

^{1}H NMR (300 MHz, $(CD_3)_2SO$)

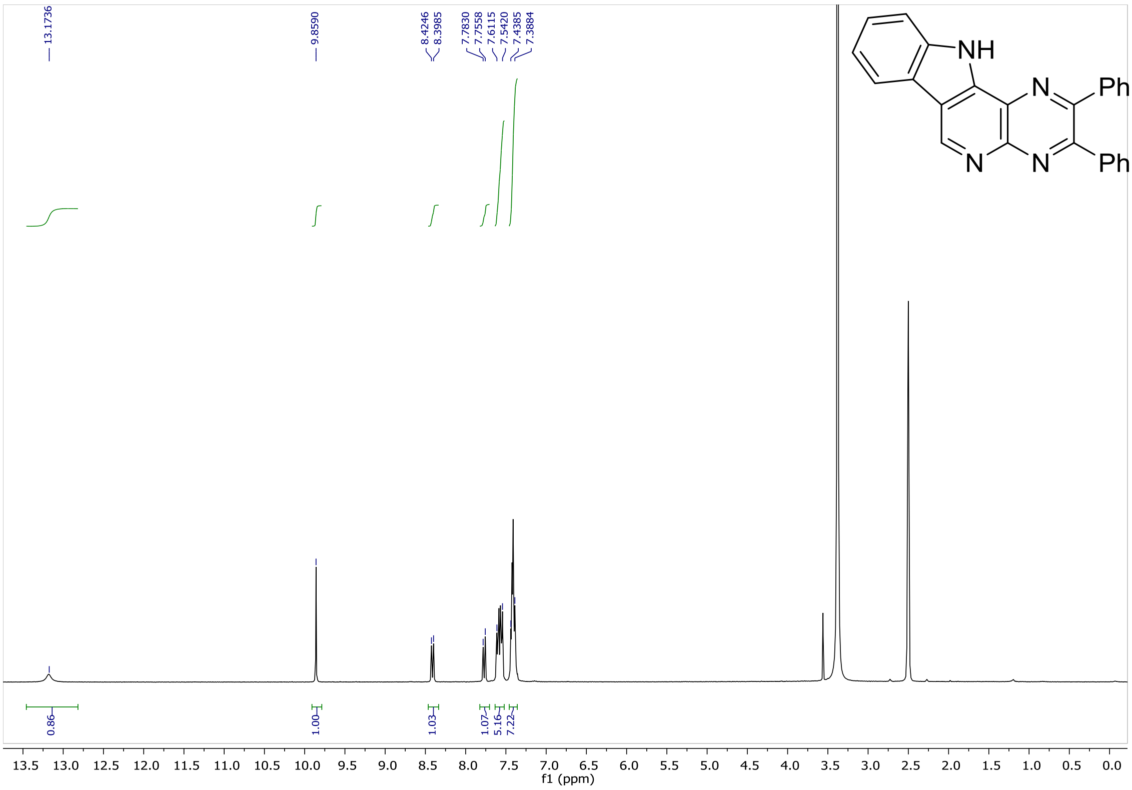

^{13}C NMR (75 MHz, $(CD_3)_2SO$)

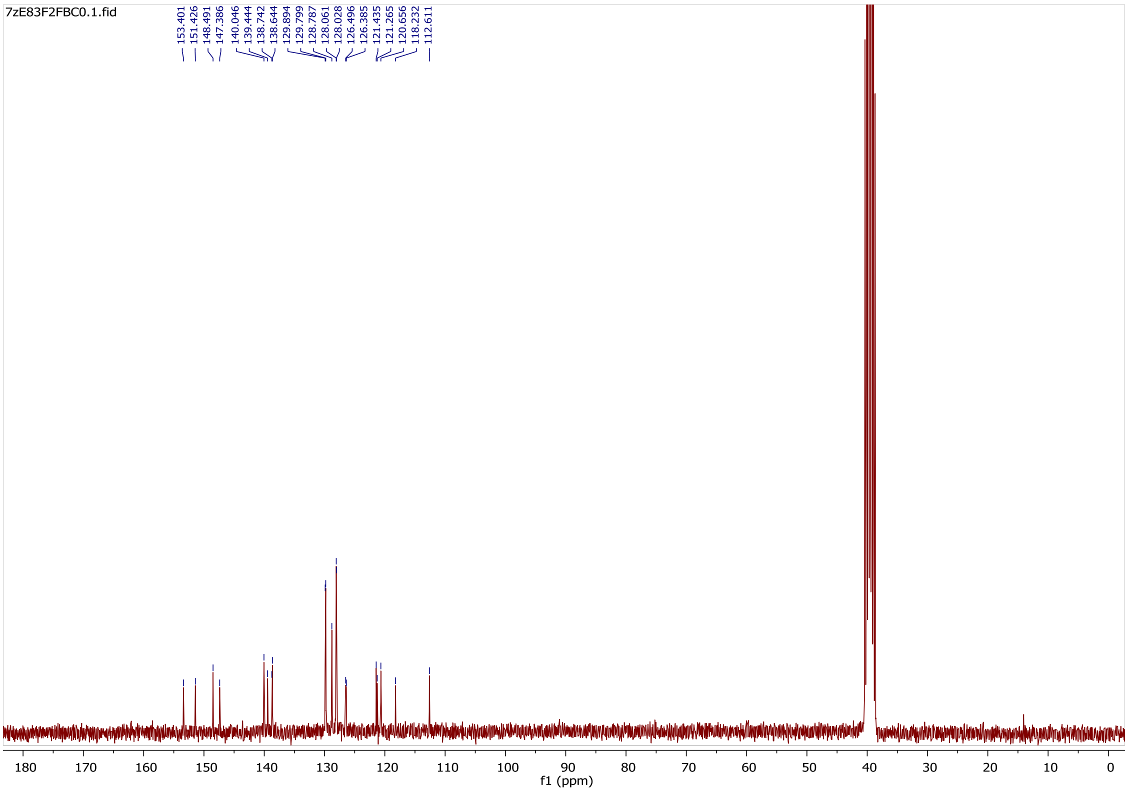

Compound 3g'

^{1}H NMR (300 MHz, $CDCl_3$)

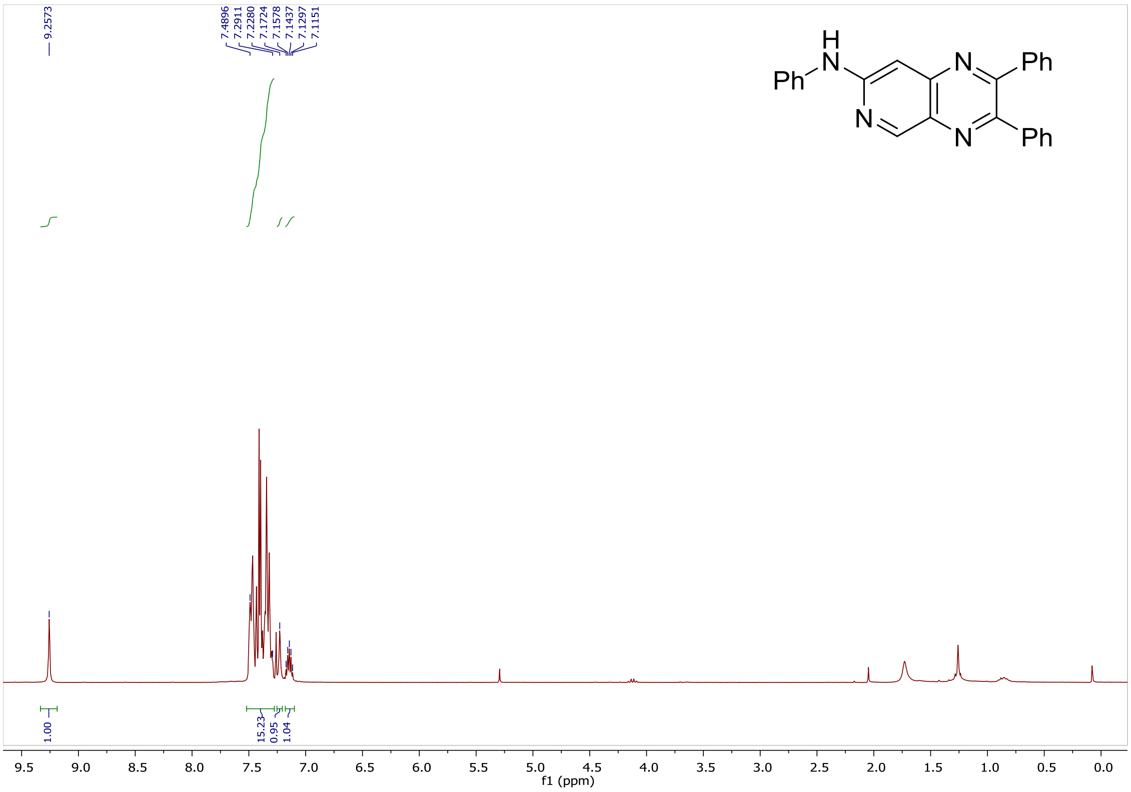

^{13}C NMR (75 MHz, $CDCl_3$)

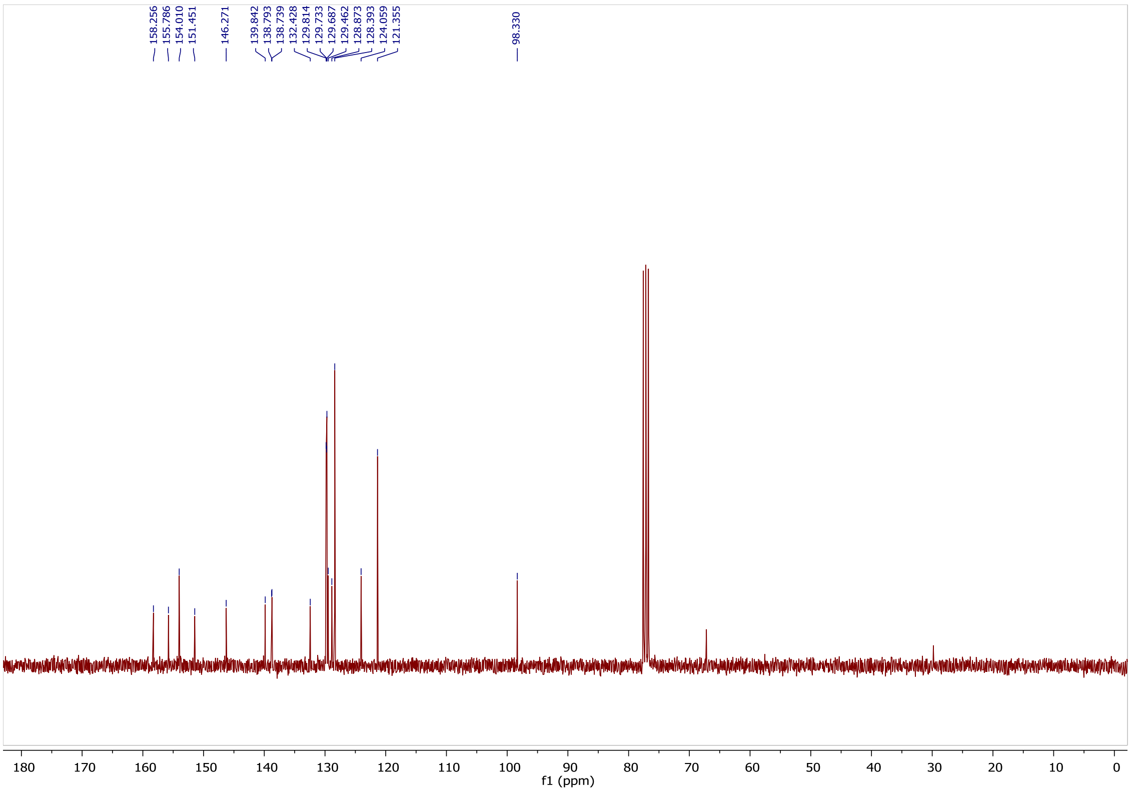

Compound 3h

^{1}H NMR (300 MHz, $CDCl_3$)

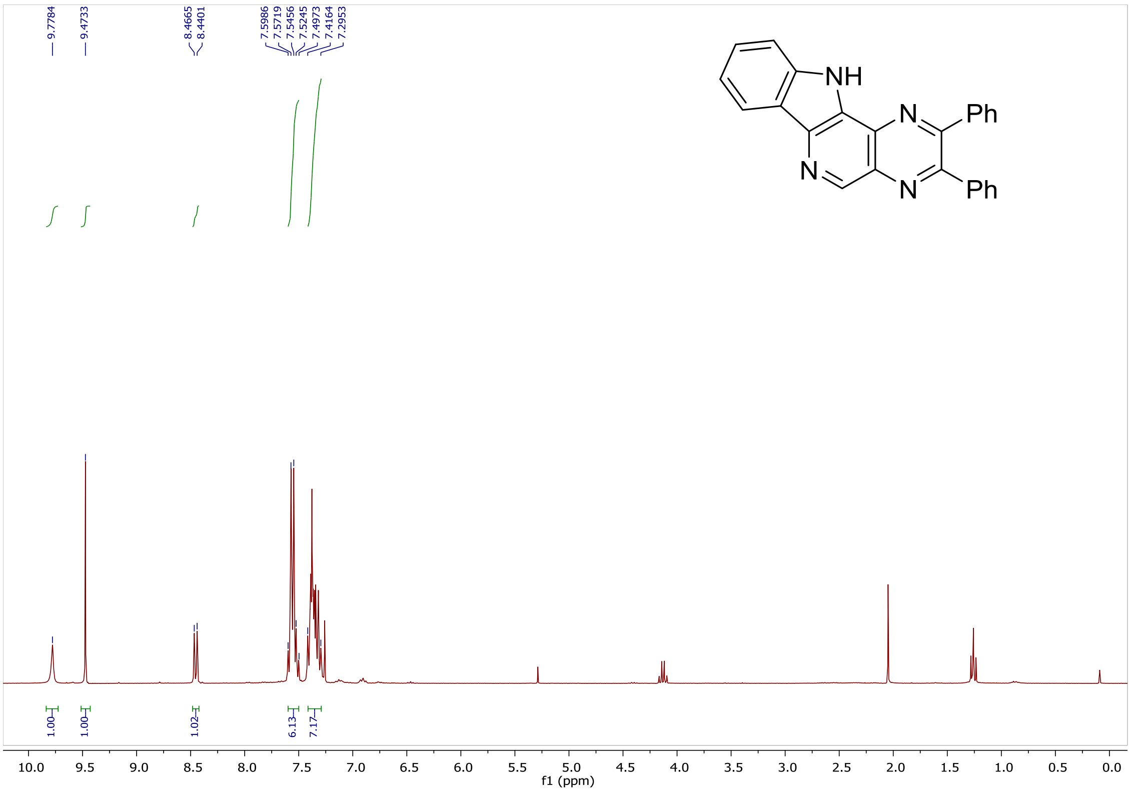

^{13}C NMR (75 MHz, $CDCl_3$)

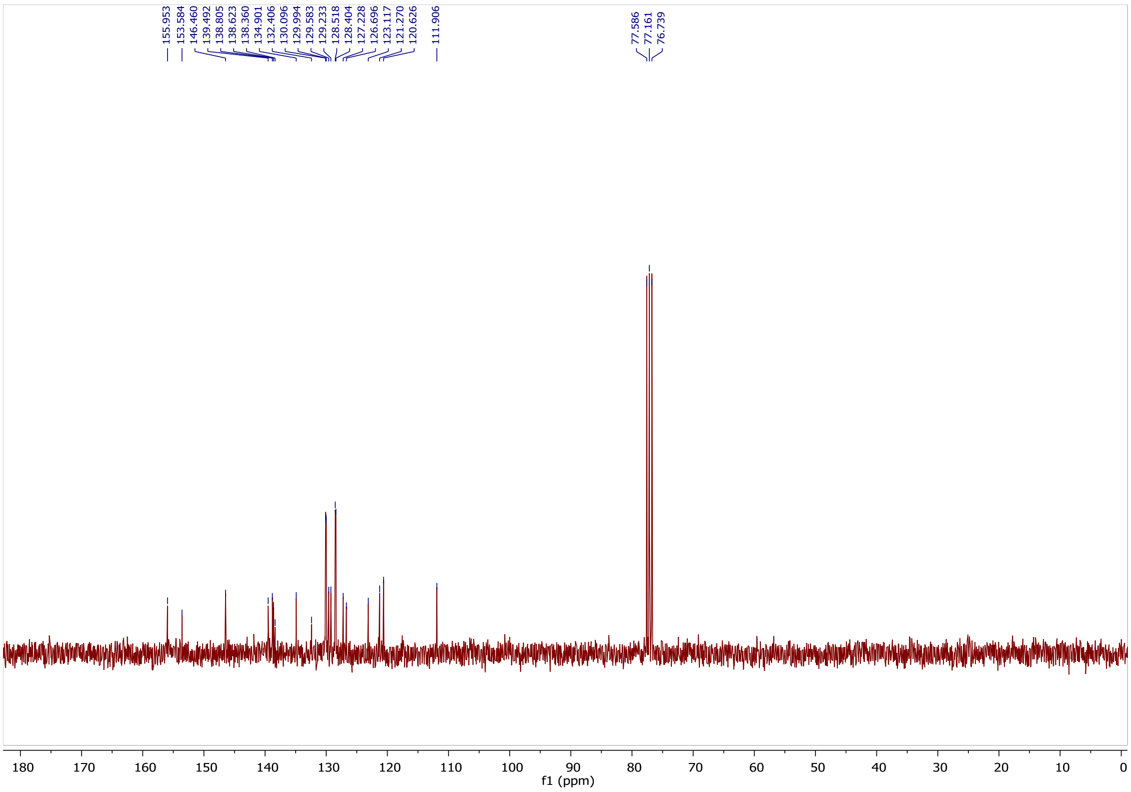

Compound 2i

^{1}H NMR (300 MHz, $CDCl_3$)

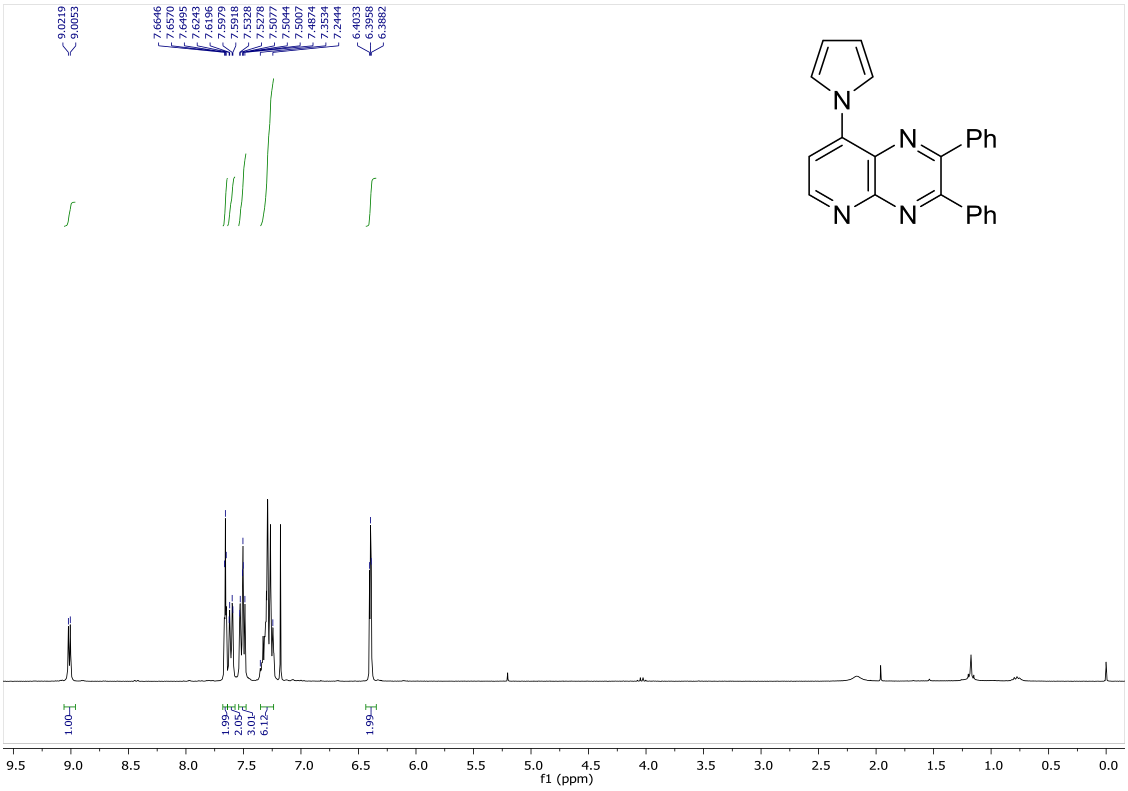

^{13}C NMR (75 MHz, $CDCl_3$)

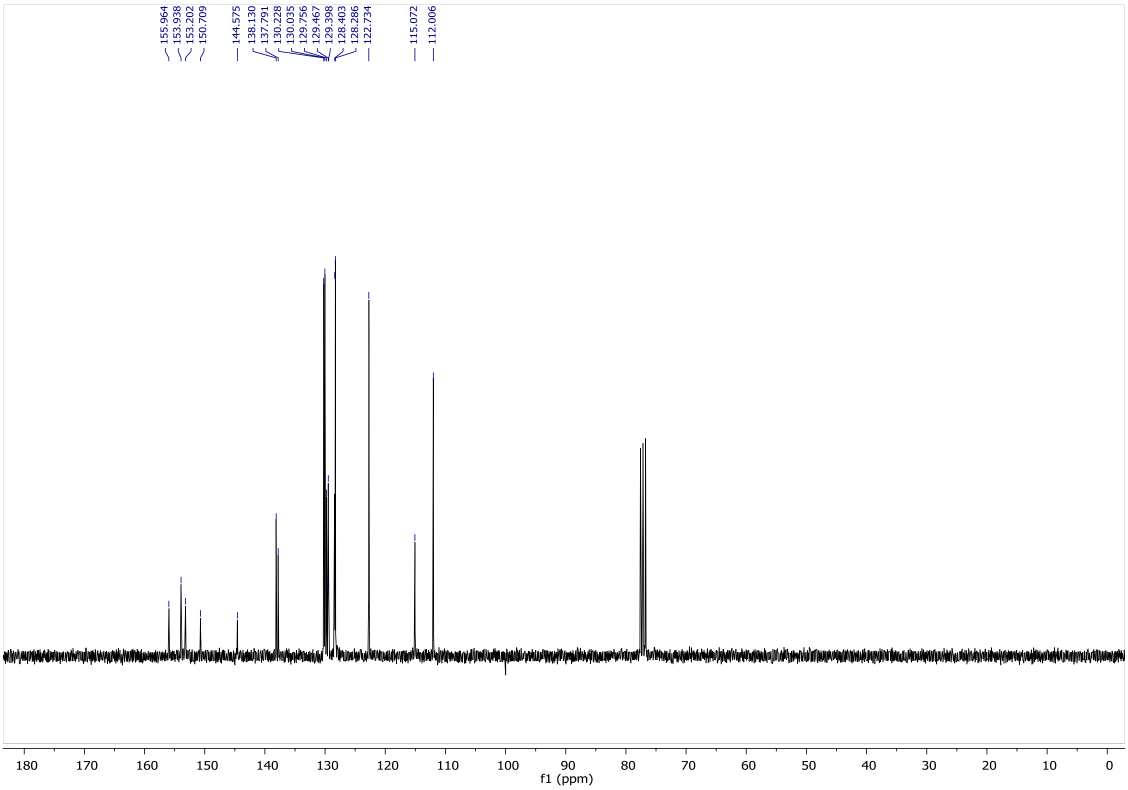

Compound 2j

^{1}H NMR (300 MHz, $CDCl_3$)

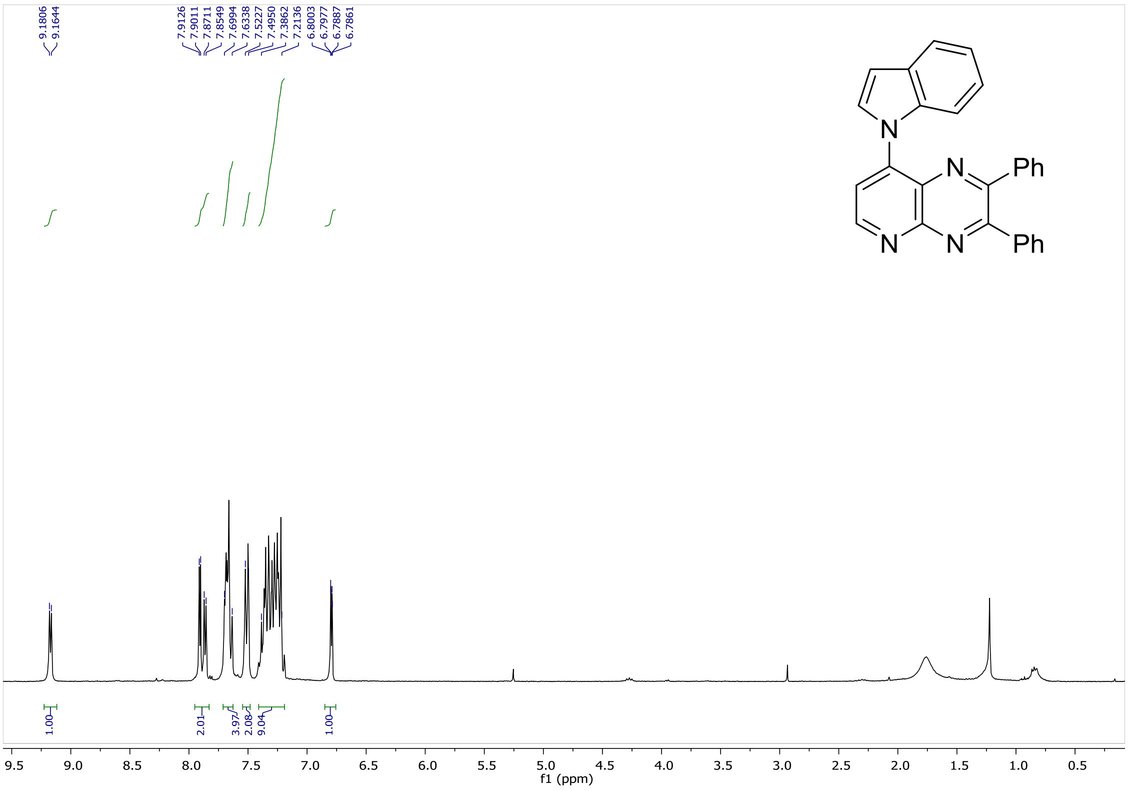

^{13}C NMR (75 MHz, $CDCl_3$)

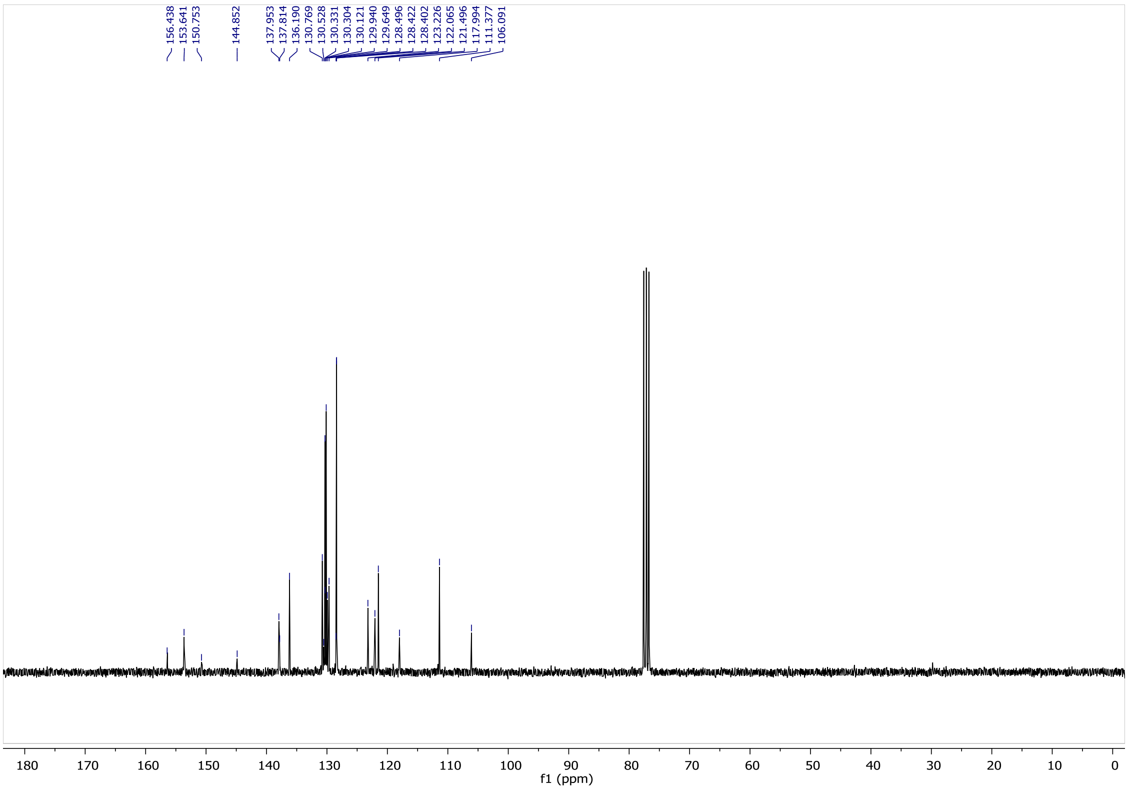

Compound 2k

^{1}H NMR (300 MHz, $CDCl_3$)

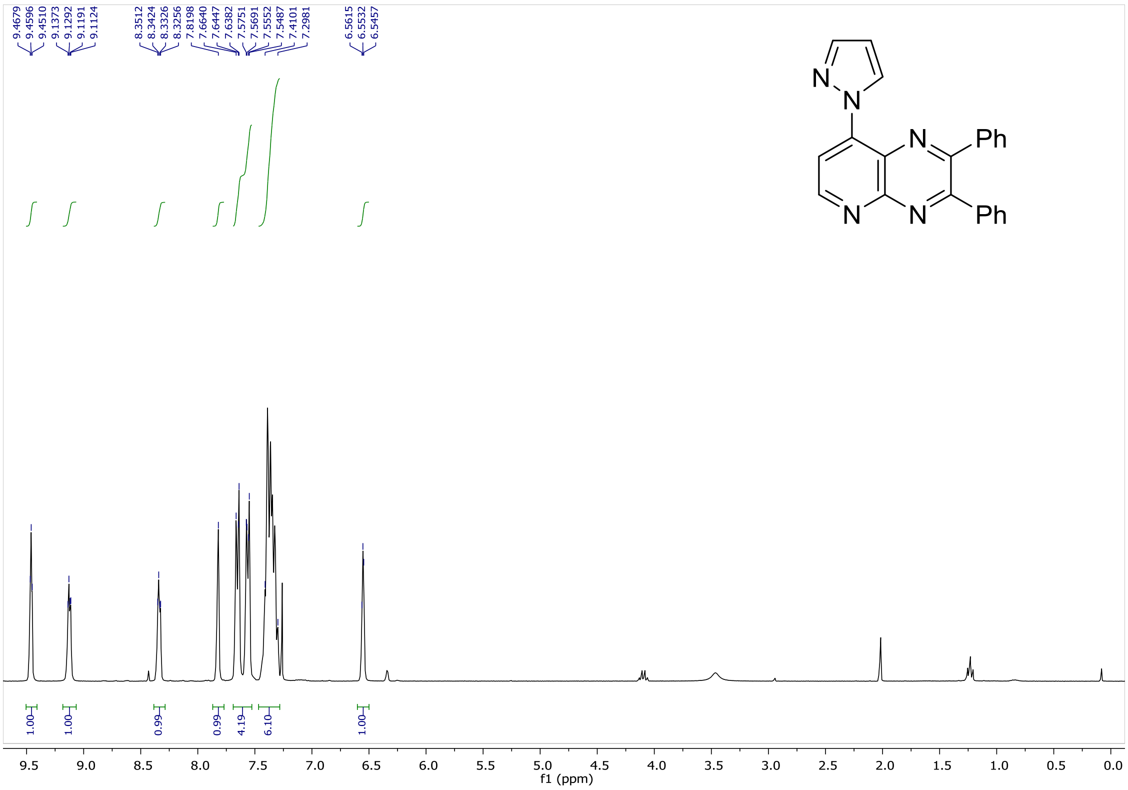

^{13}C NMR (75 MHz, $CDCl_3$)

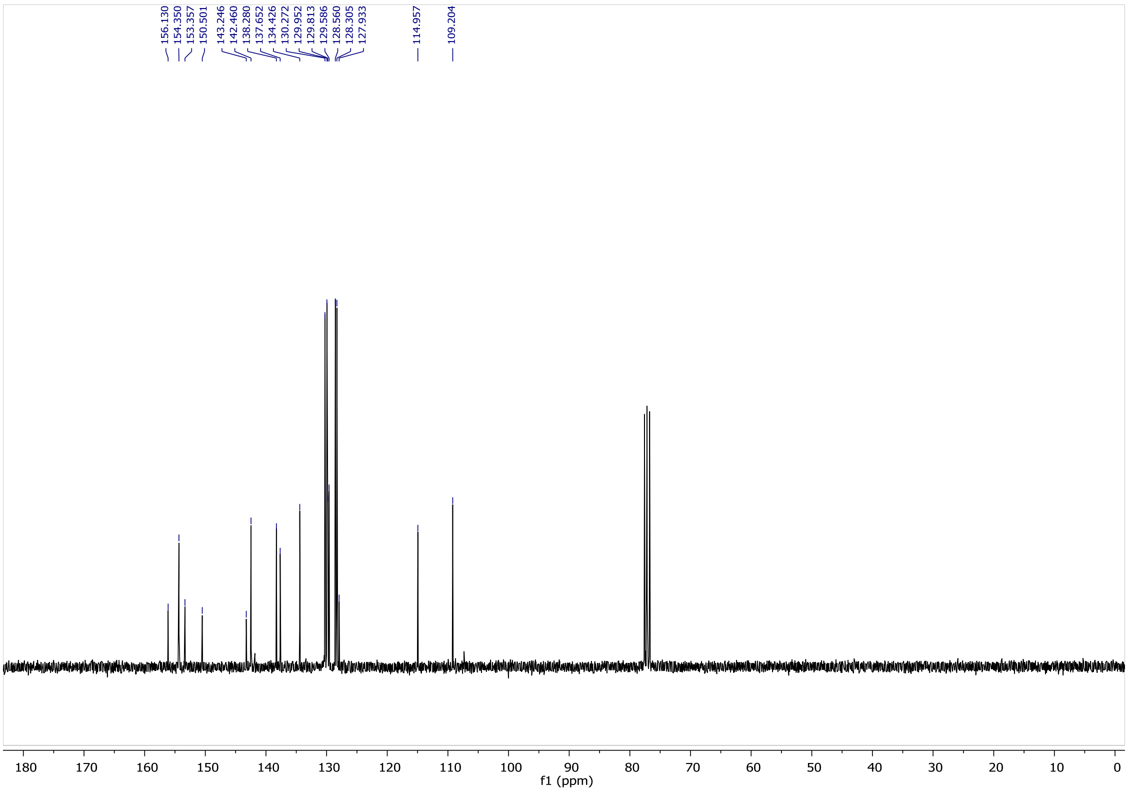

Compound 2l

^{1}H NMR (300 MHz, $CDCl_3$)

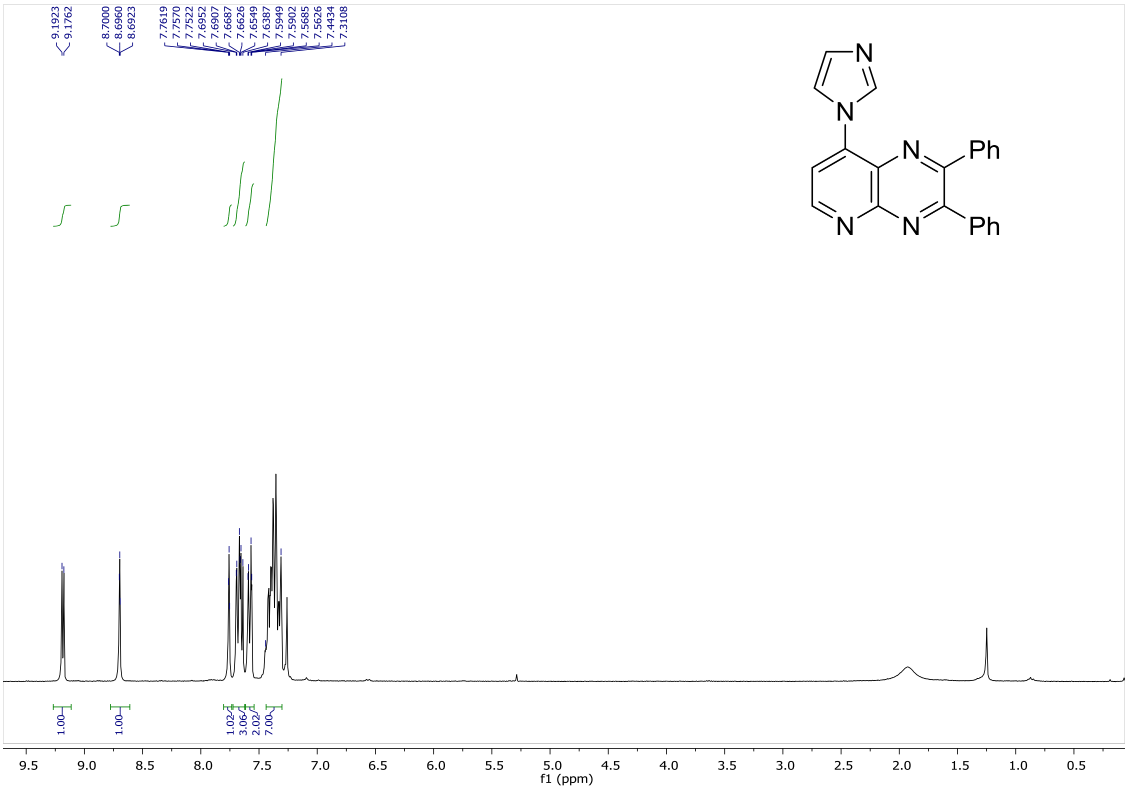

^{13}C NMR (75 MHz, $CDCl_3$)

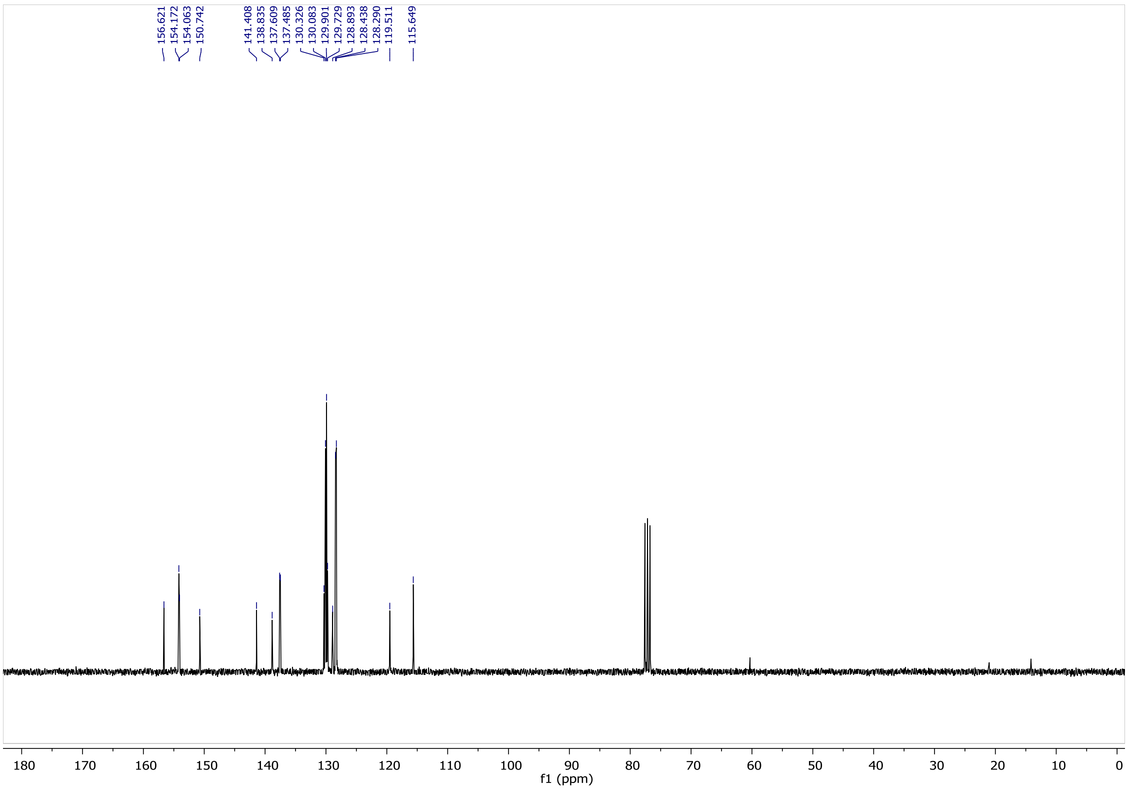

Compound 2m

^{1}H NMR (300 MHz, $CDCl_3$)

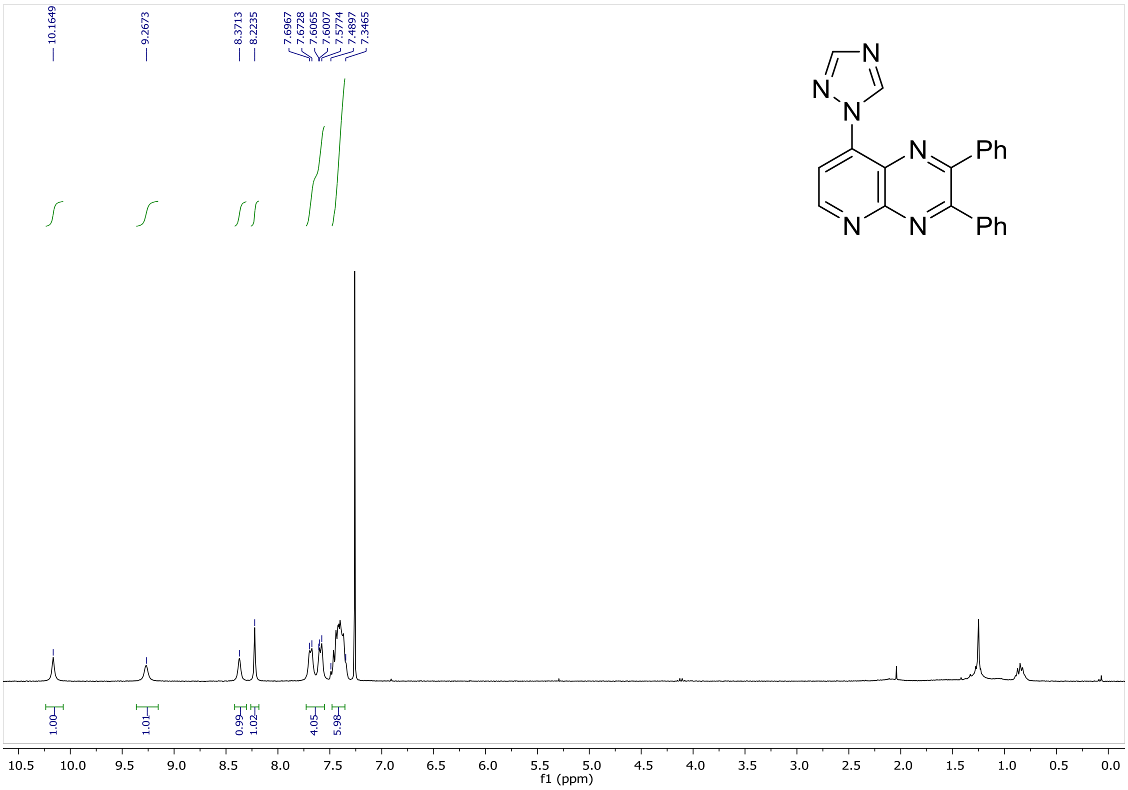

^{13}C NMR (75 MHz, $CDCl_3$)

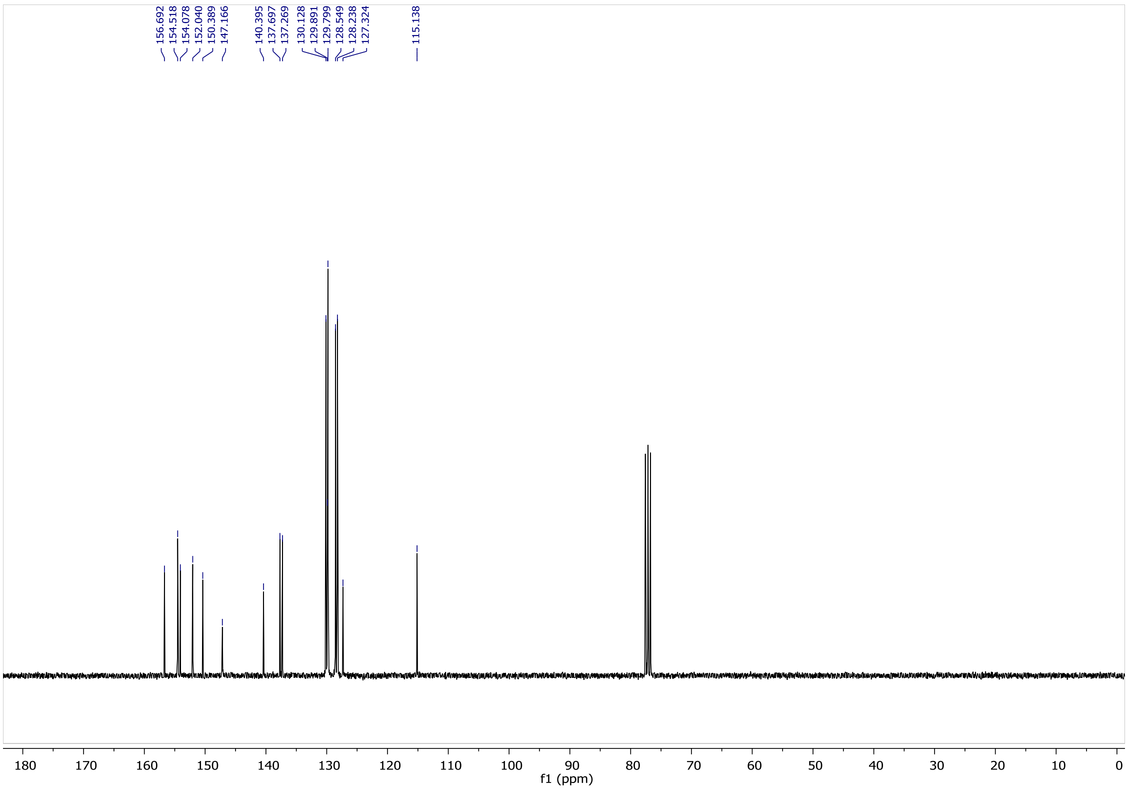

Compound 1k’

^{1}H NMR (300 MHz, $CDCl_3$)

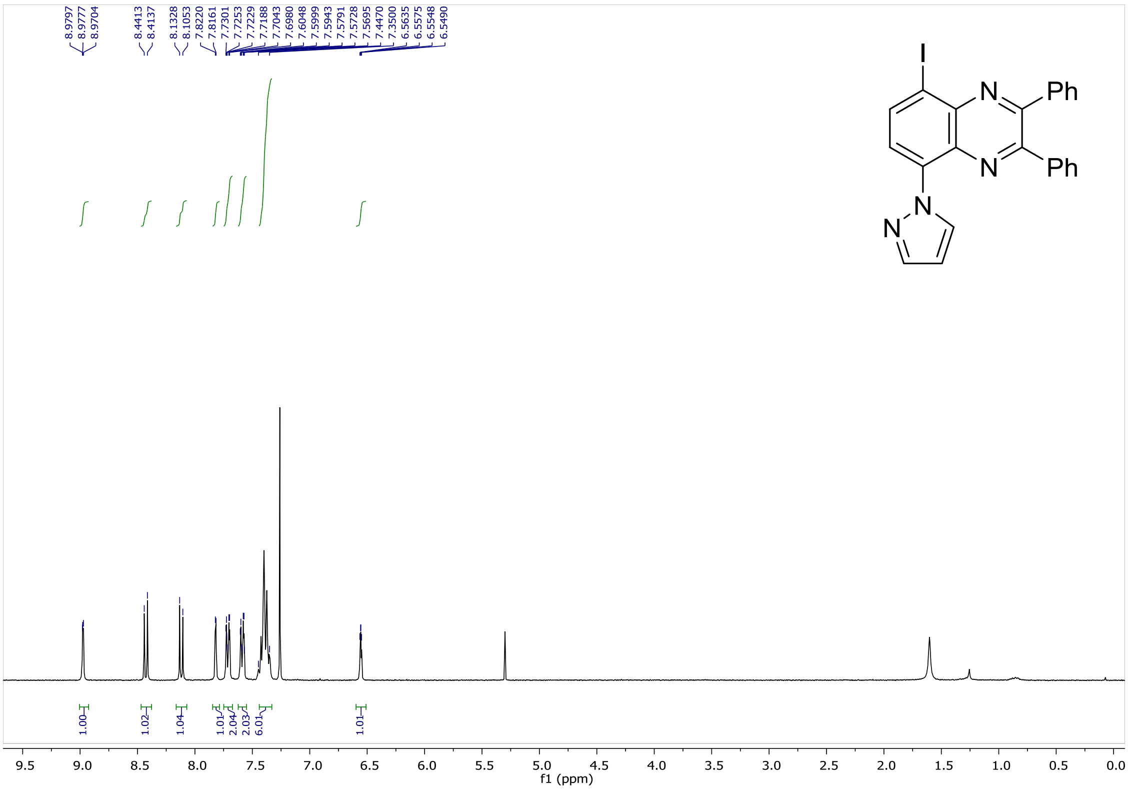

^{13}C NMR (75 MHz, $CDCl_3$)

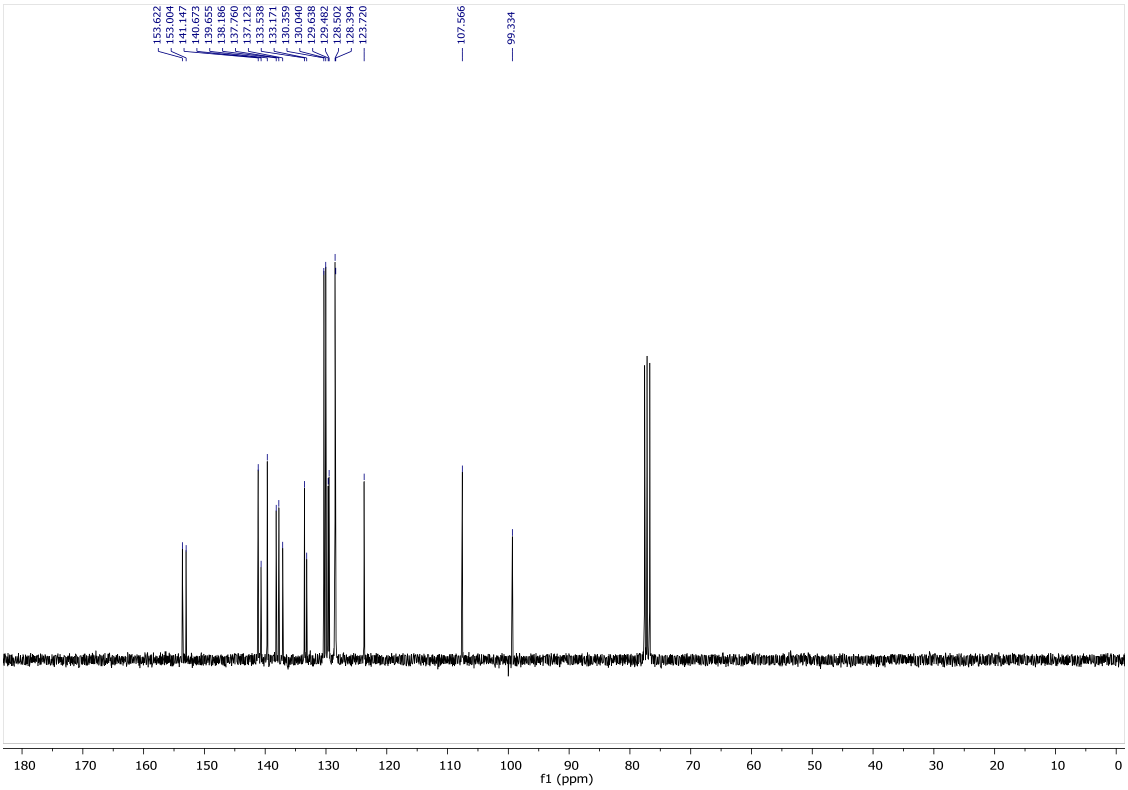

Compound 2n

^{1}H NMR (300 MHz, $CDCl_3$)

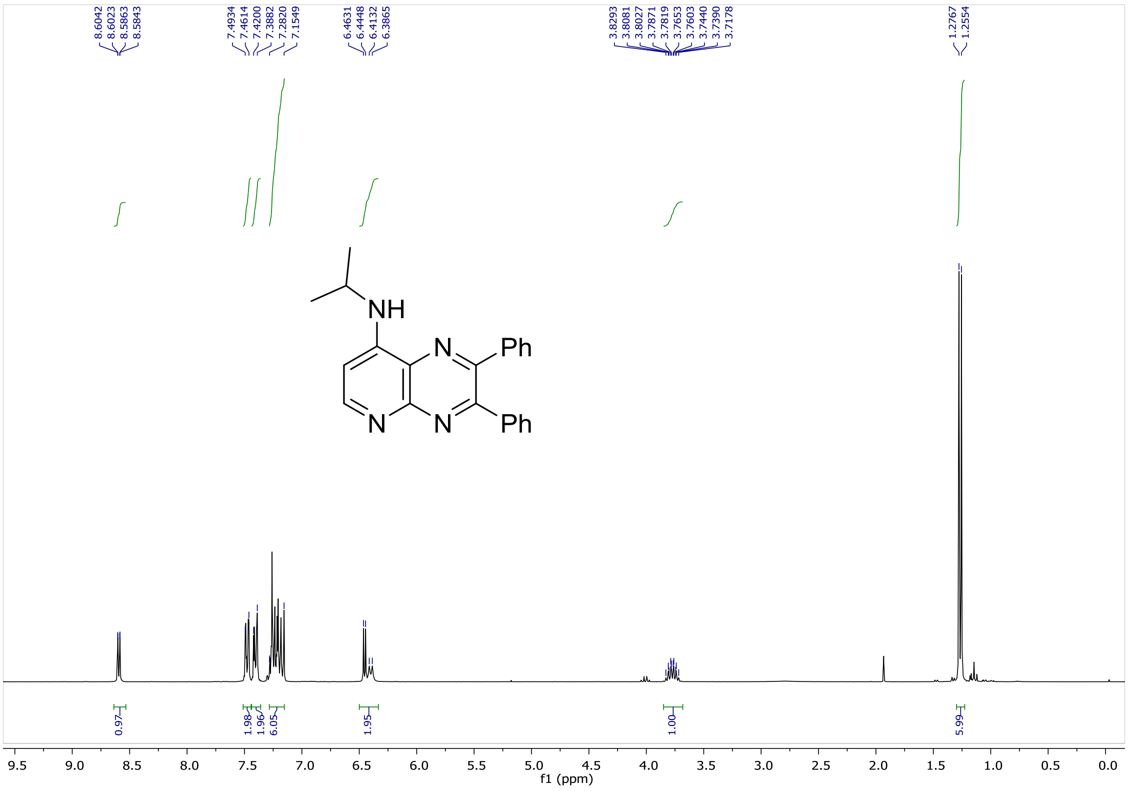

^{13}C NMR (75 MHz, $CDCl_3$)

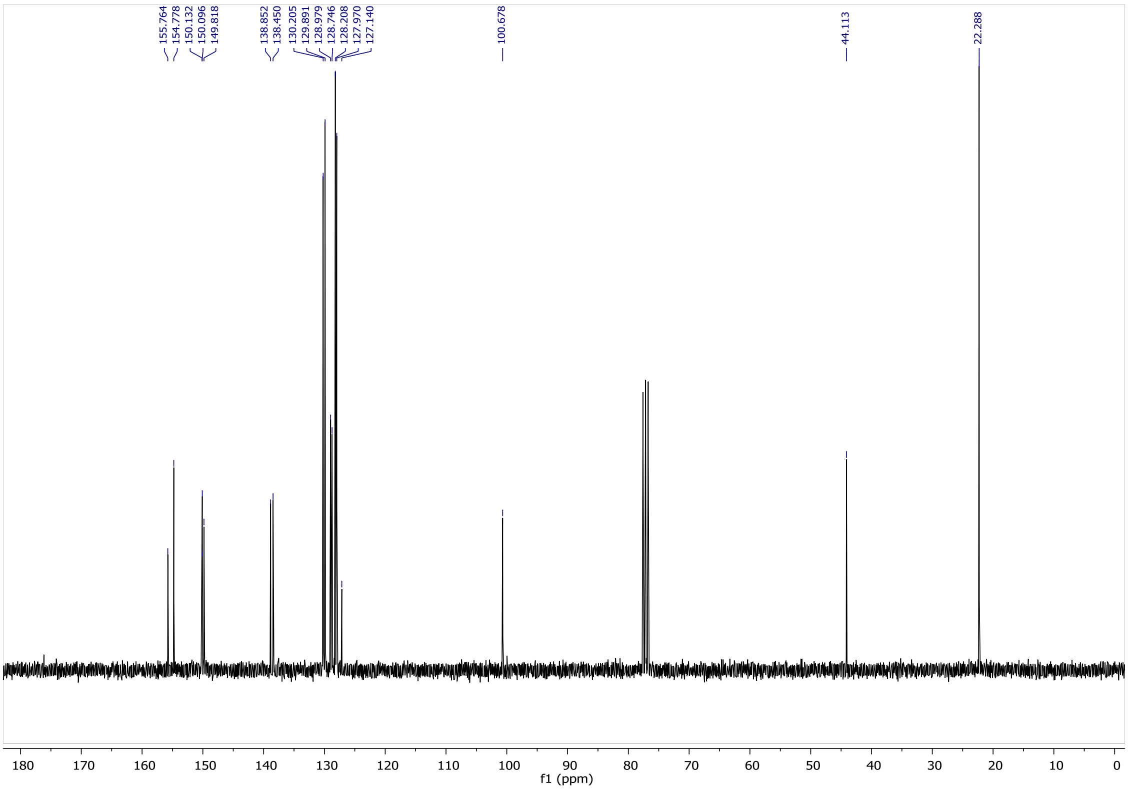

Compound 2o

^{1}H NMR (300 MHz, $CDCl_3$)

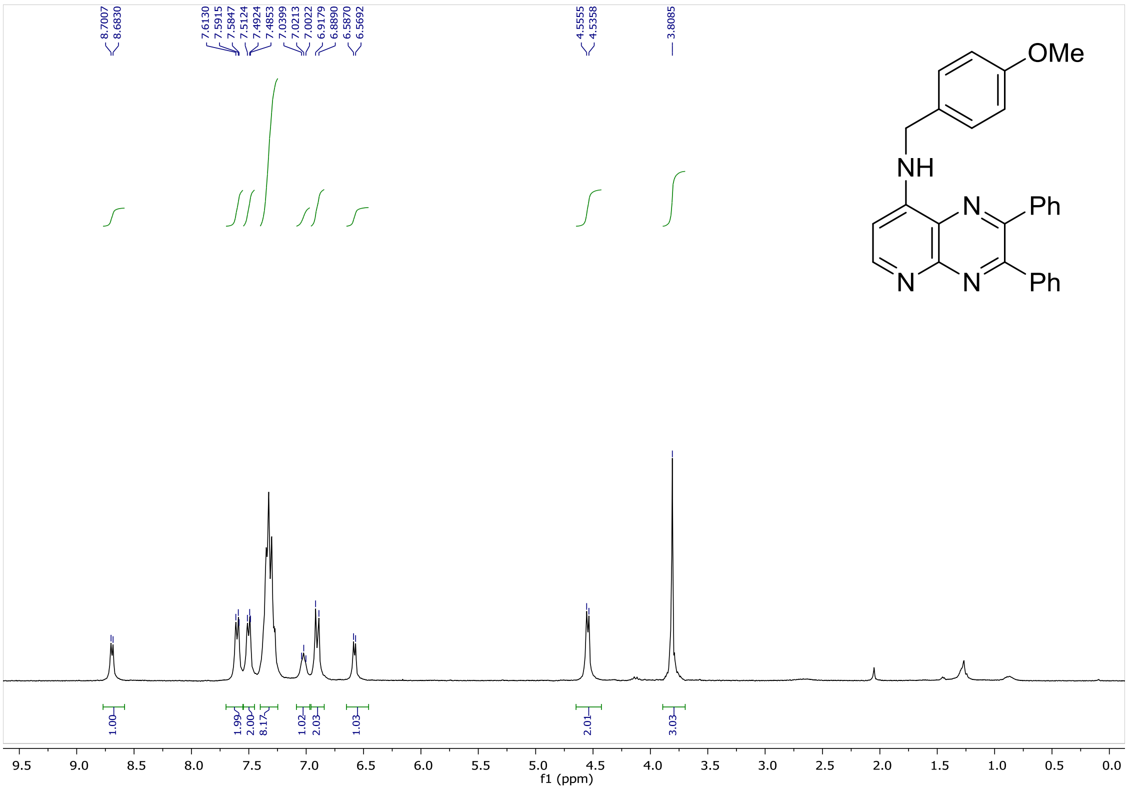

^{13}C NMR (75 MHz, $CDCl_3$)

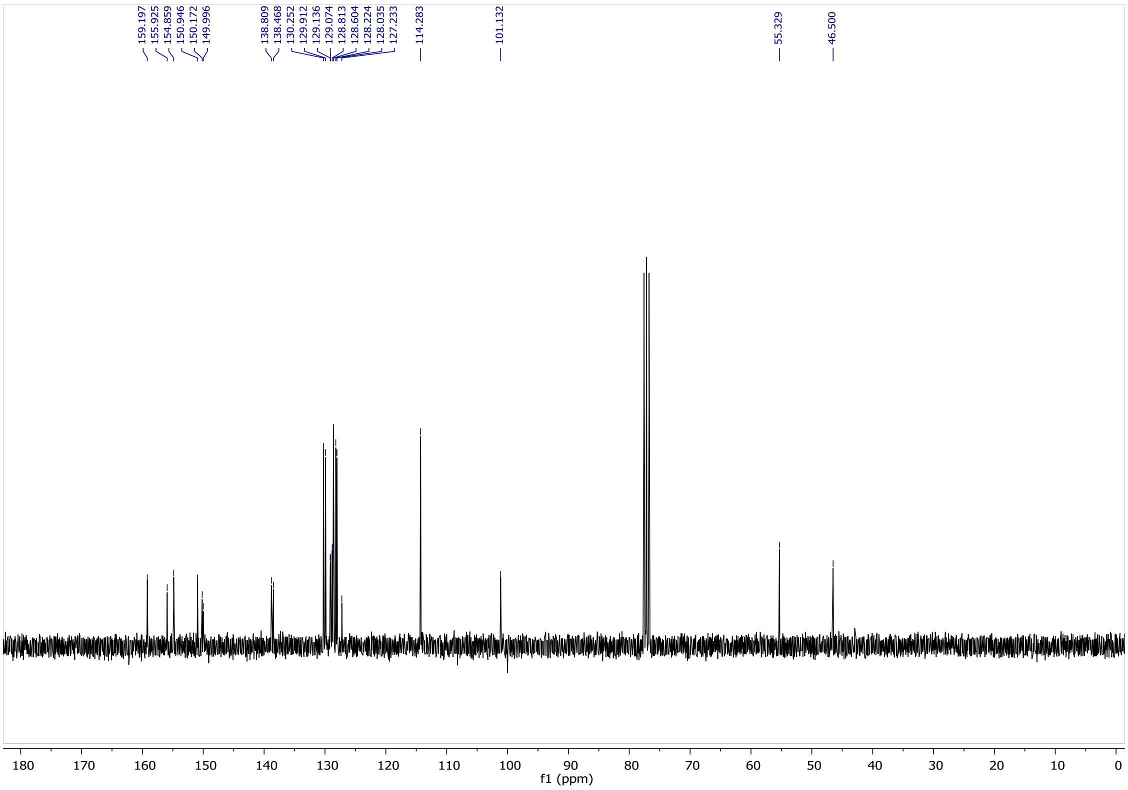

Compound 2p

^{1}H NMR (300 MHz, $CDCl_3$)

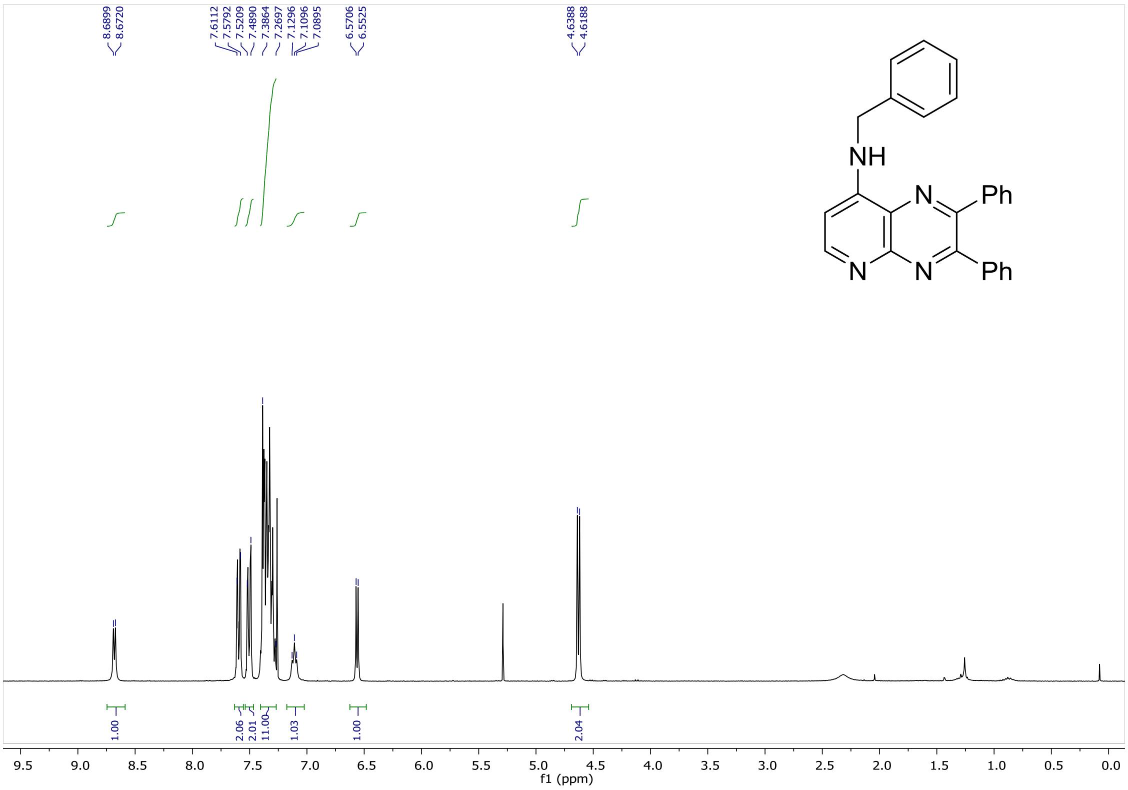

^{13}C NMR (75 MHz, $CDCl_3$)

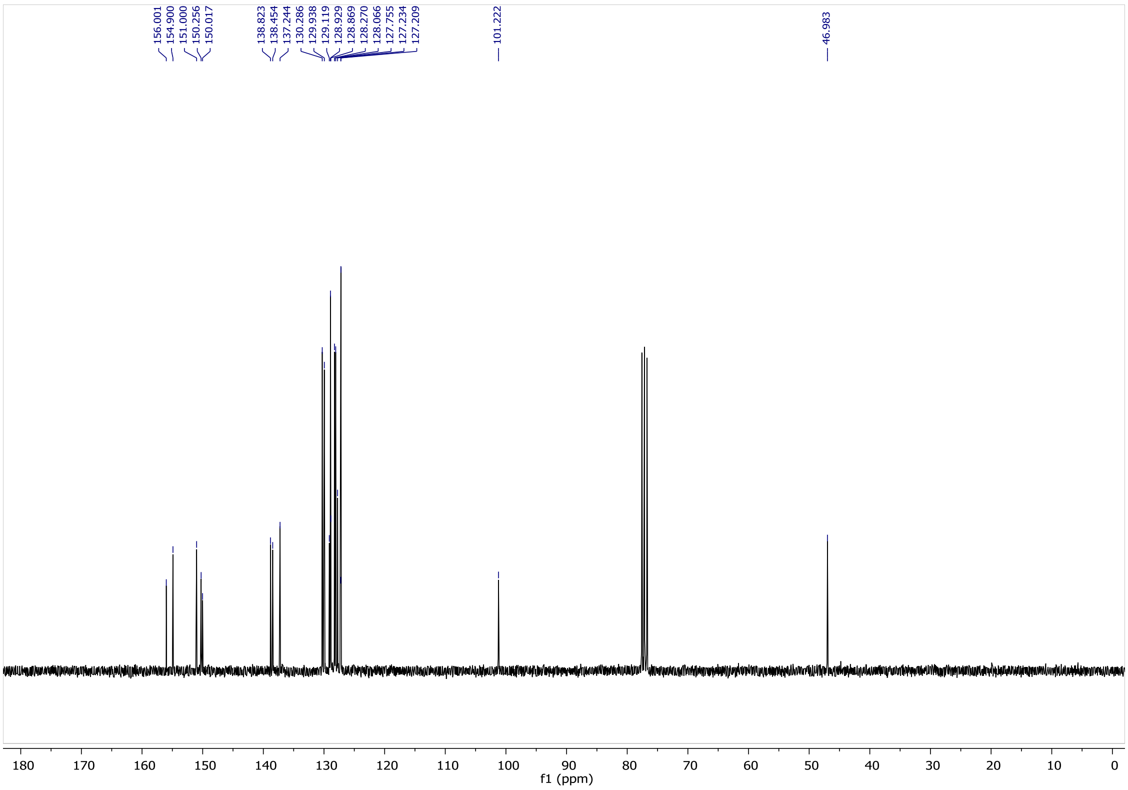

Compound 2q

^{1}H NMR (300 MHz, $CDCl_3$)

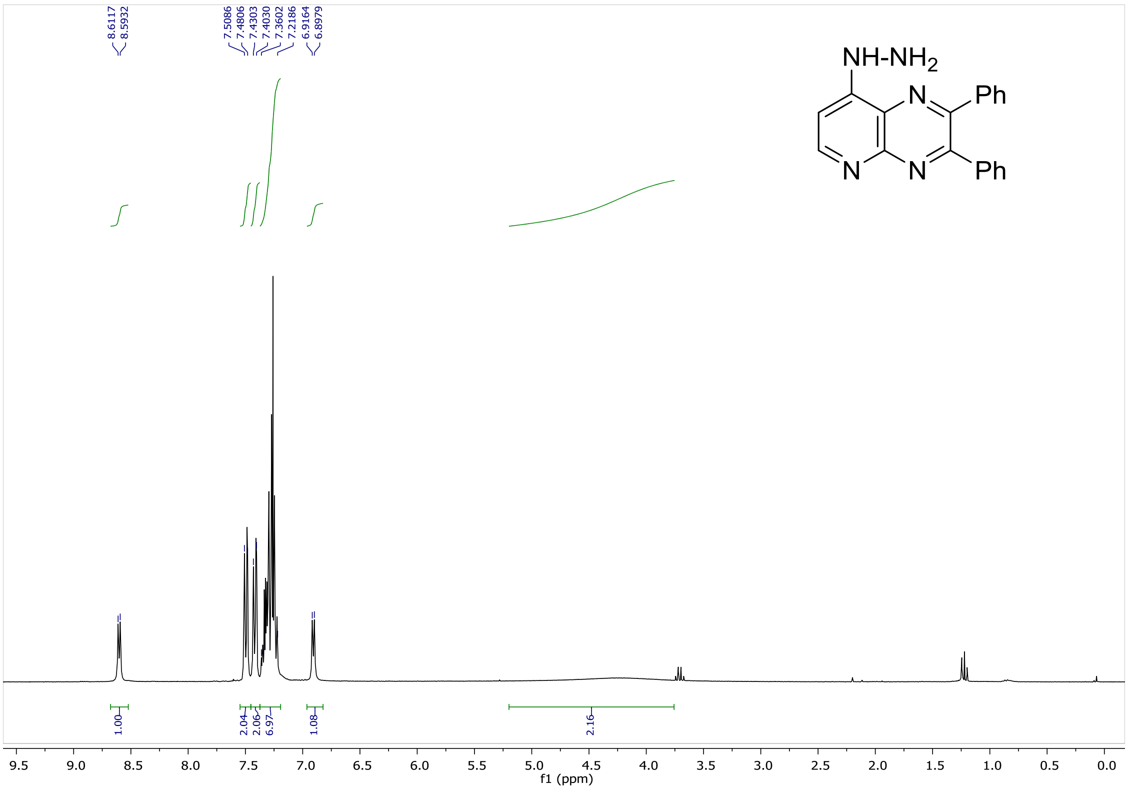

^{13}C NMR (75 MHz, $CDCl_3$)

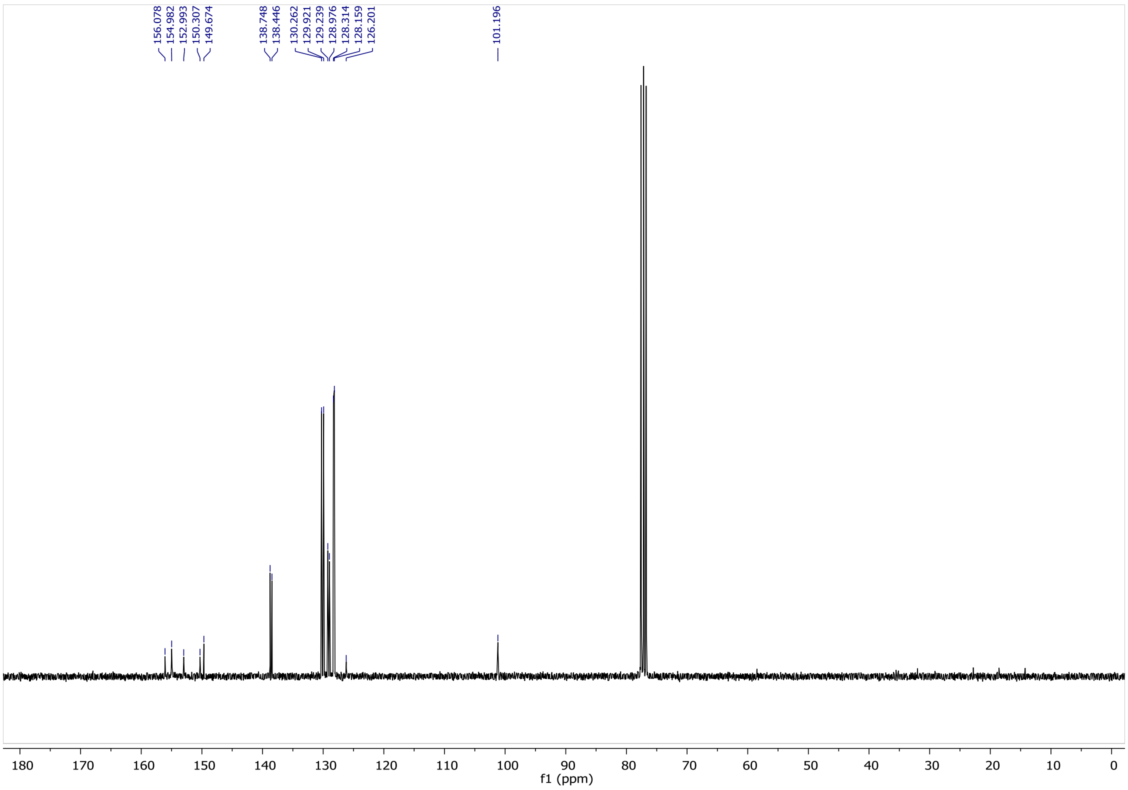

Compound 2r

^{1}H NMR (300 MHz, $CDCl_3$)

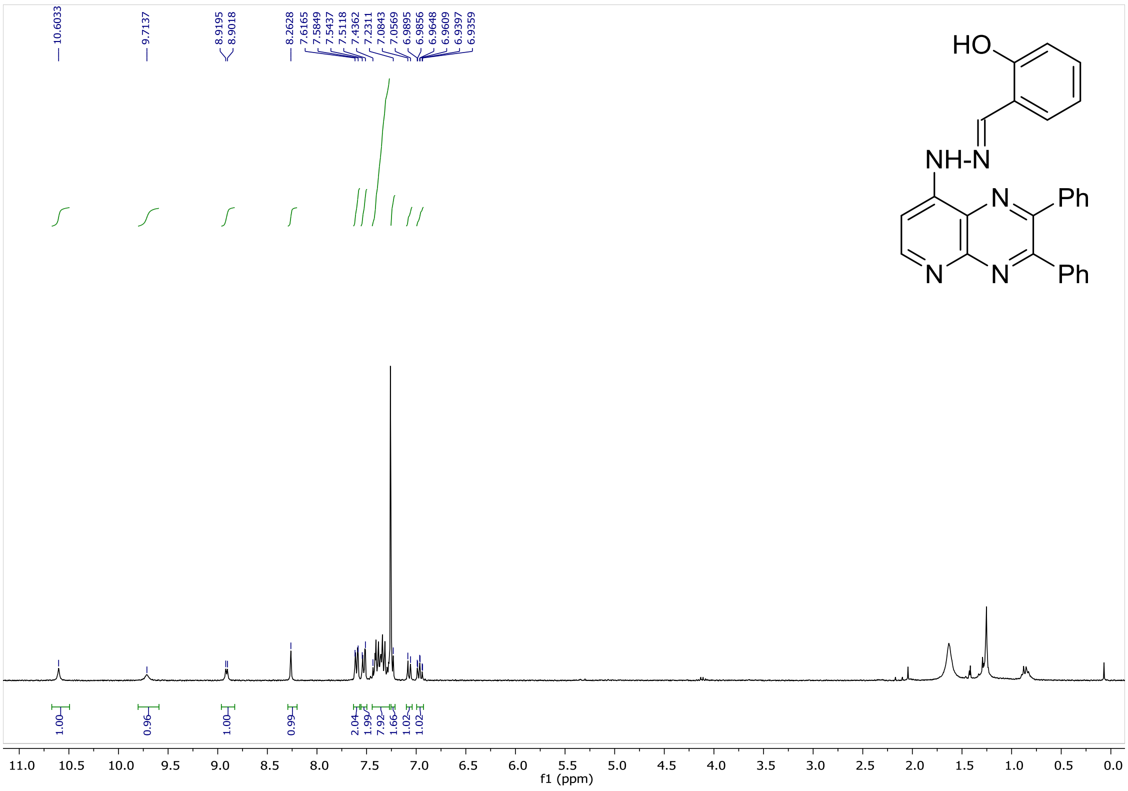

Compound 2t

^{1}H NMR (300 MHz, $CDCl_3$)

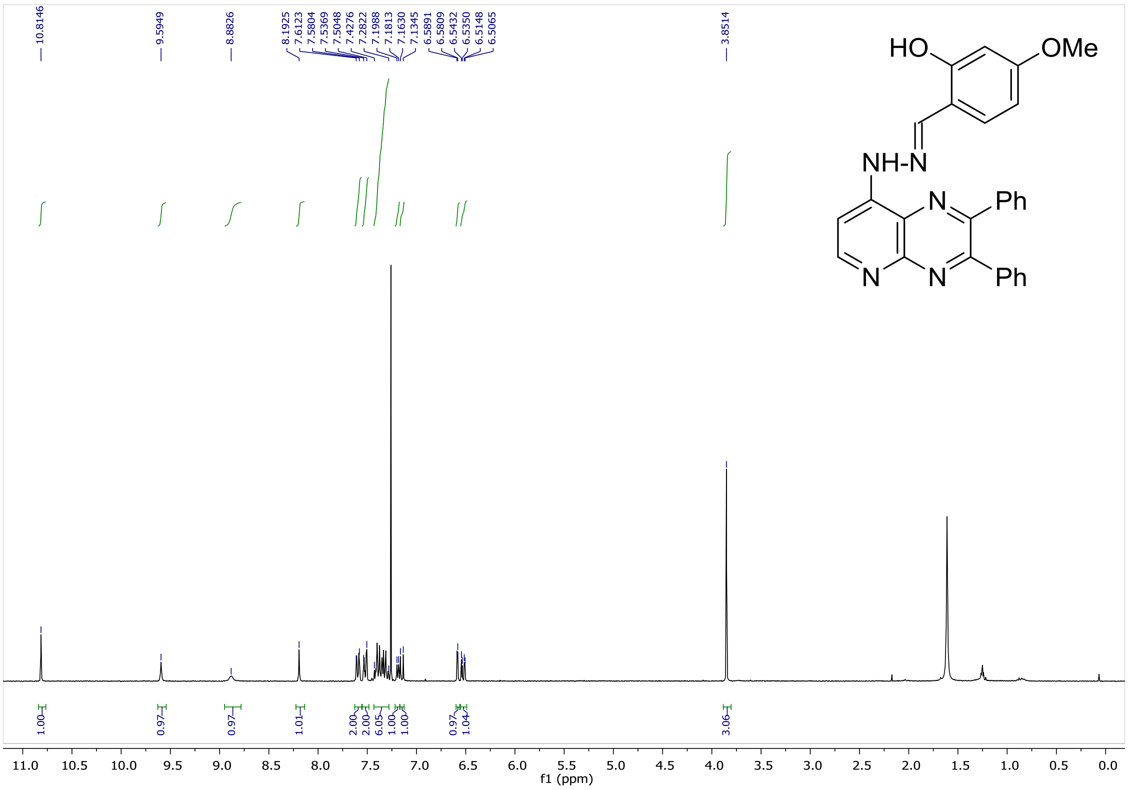

Compound 2s

^{1}H NMR (300 MHz, $CDCl_3$)

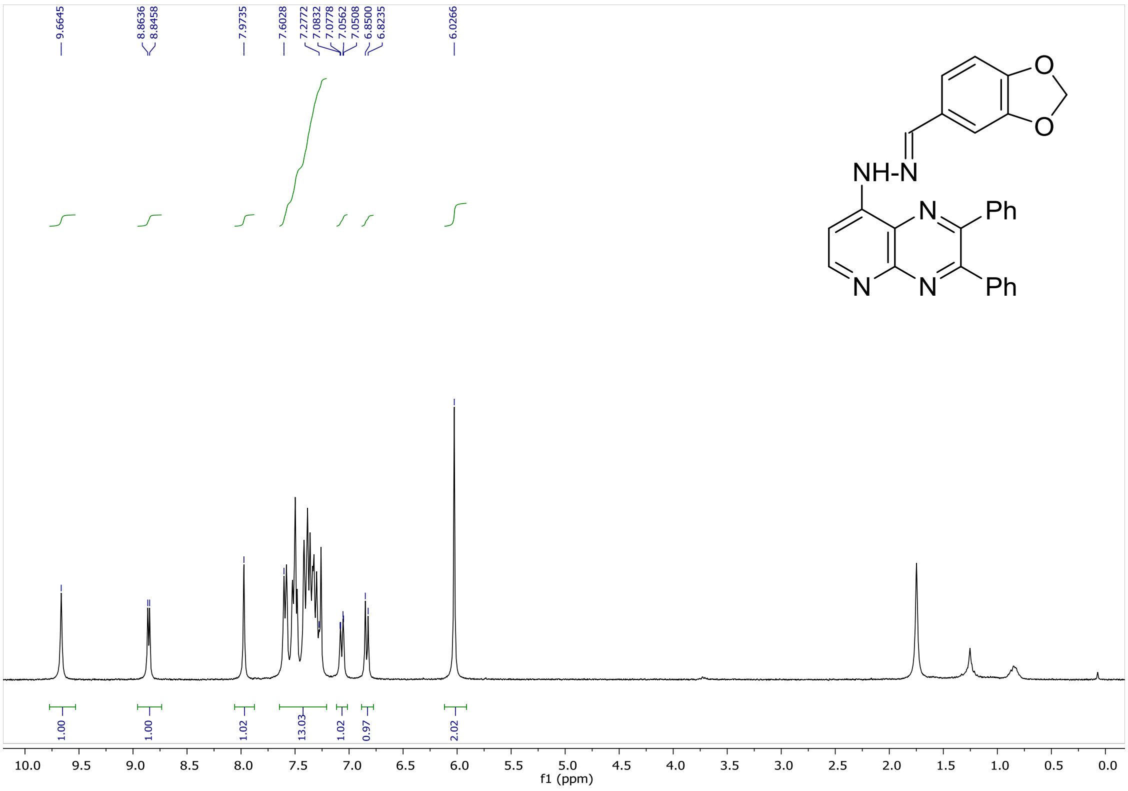

^{13}C NMR (75 MHz, $(CD_3)_2SO$)

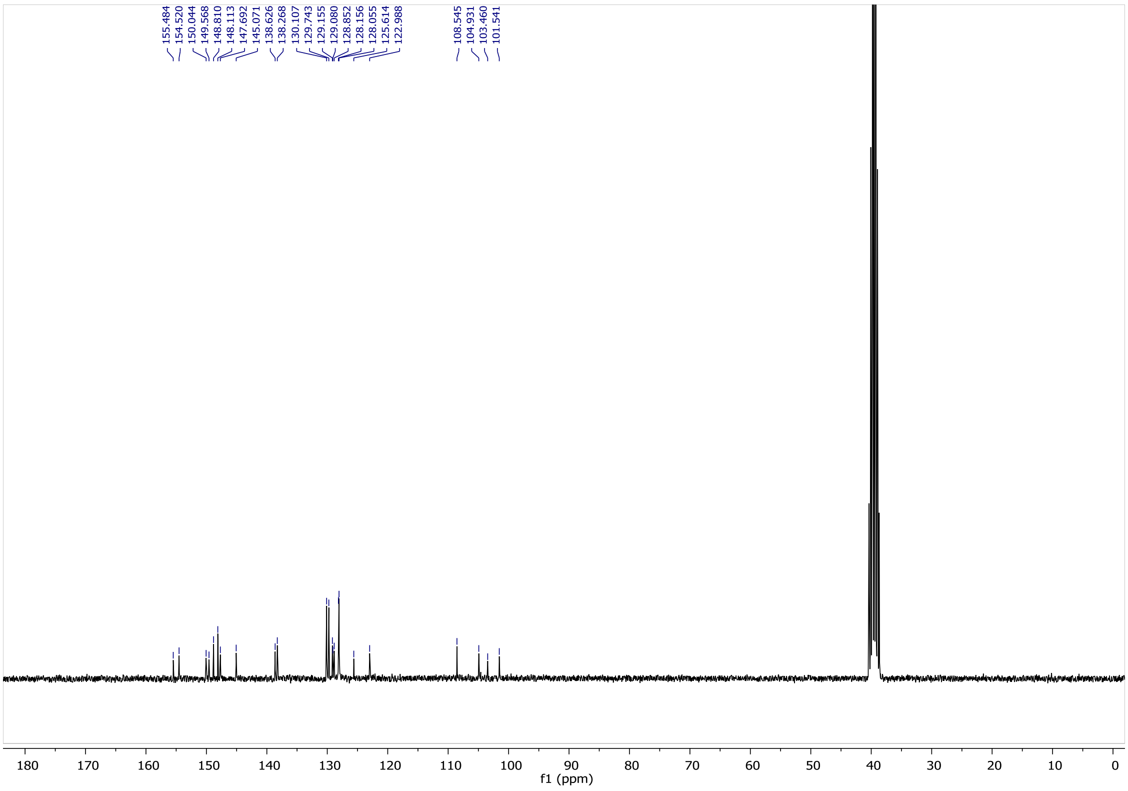

Compound 2u

^{1}H NMR (300 MHz, $CDCl_3$)

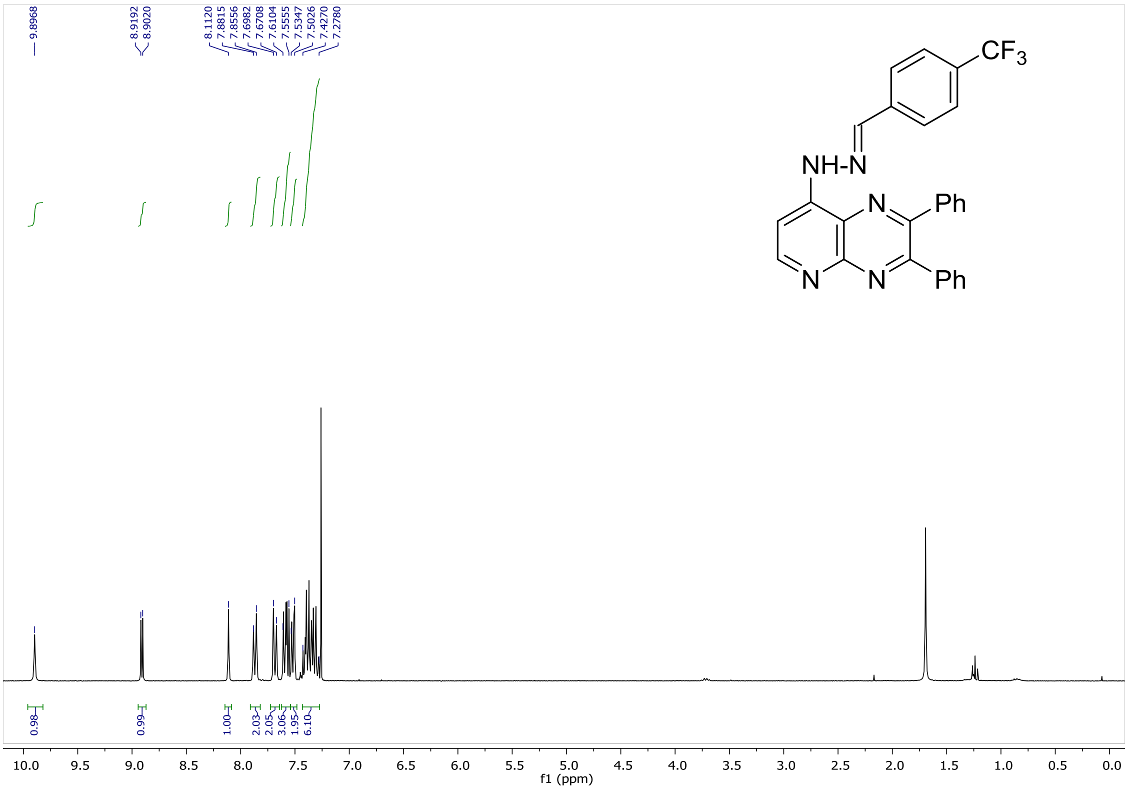

^{13}C NMR (126 MHz, $(CD_3)_2SO$, 333 K)

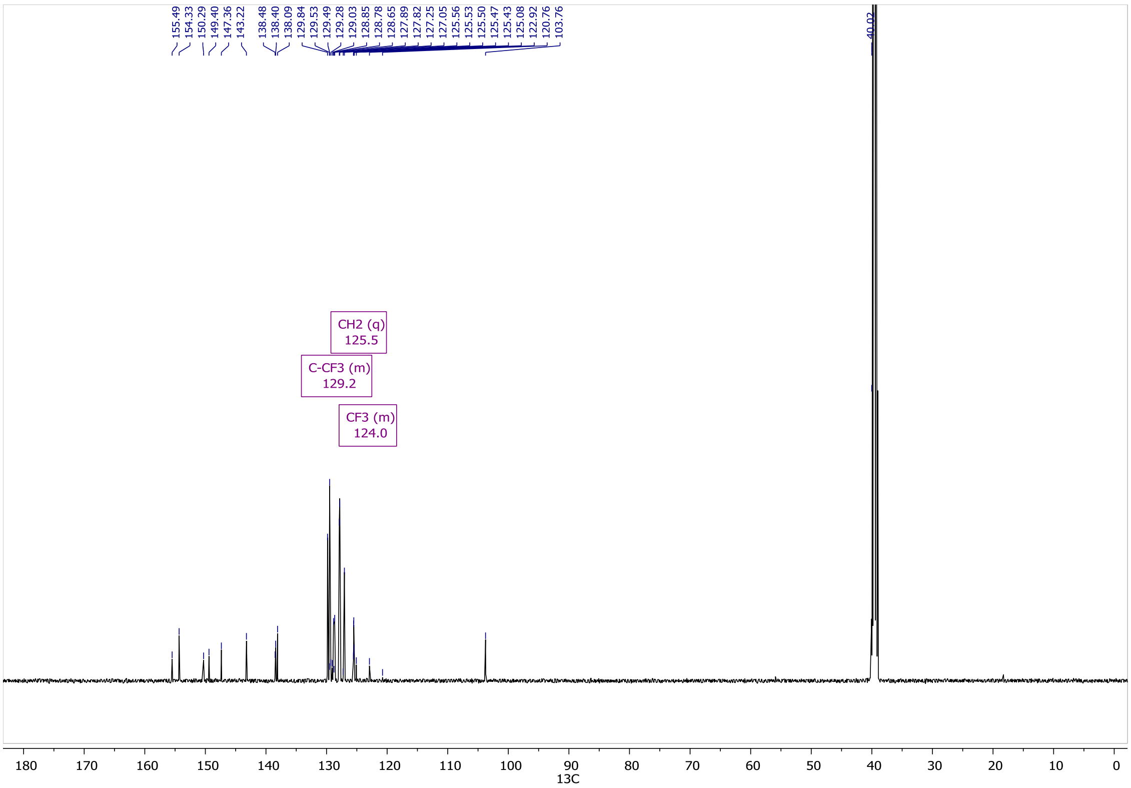

Compound 2v

^{1}H NMR (300 MHz, $CDCl_3$)

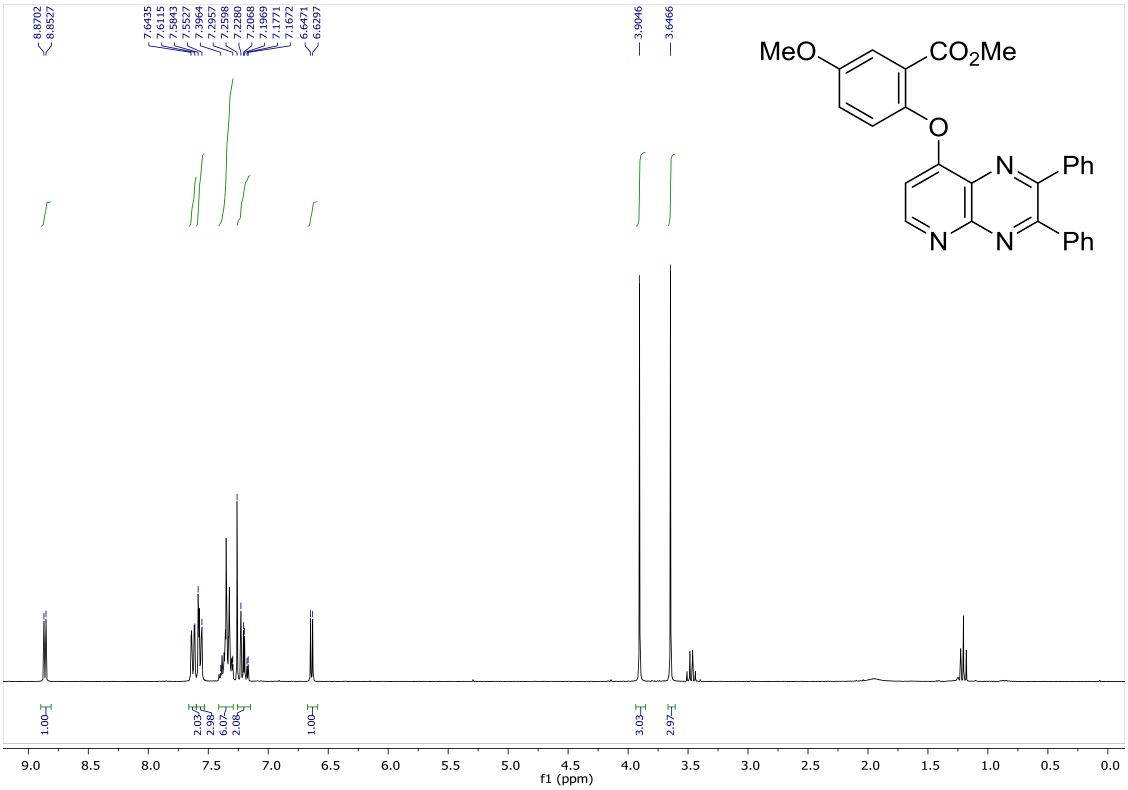

^{13}C NMR (75 MHz, $CDCl_3$)

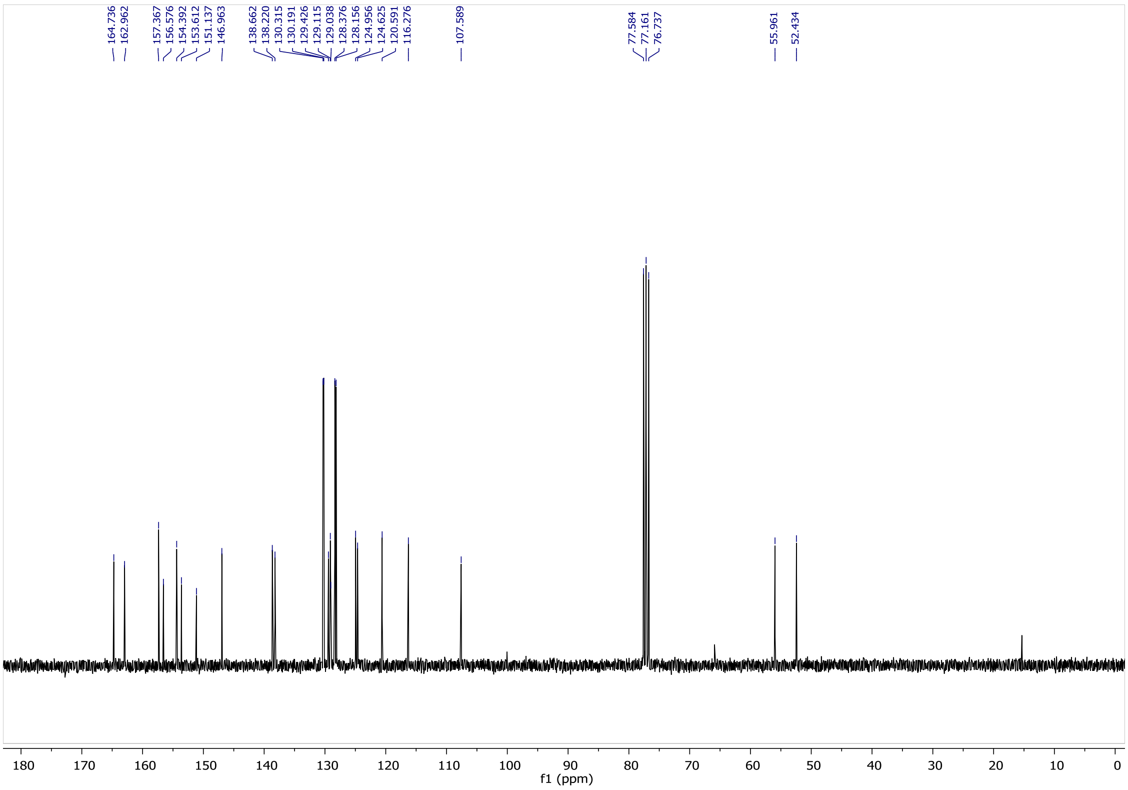

Inhibitory activities of synthesized compounds against a short panel of disease-related protein kinases

Table S1. The table displays the remaining kinase activities detected after treatment with 10 µM of the tested compounds. The values obtained after treatment with 1 µM are given in brackets. Results are expressed in % of maximal activity, i.e. measured in the absence of inhibitor but with an equivalent dose of DMSO (solvent of the tested compounds). ATP concentration used in the kinase assays was 10 µmol/L (values are means, n=2). Kinases are from human origin unless specified: *Mm, Mus musculus; Rn, Rattus norvegicus; Ssc, Sus scrofa domesticus.*

Compound	CDK2/Cyclin A	CDK5/p25	CDK9/Cyclin T	PIM1	*Mm*CLK1	*Rn*DYRK1A	DYRK1A	*Ssc*GSK3α/β	GSK3β	*Ssc*CK1δ/ε	CK1ε	Haspin
**1c**	99 (99)	- [1]	84 (97)	94 (100)	113 (108)	138 (146)	- [1]	110 (100)	- [1]	100 (114)	- [1]	108 (104)
**1d**	103 (95)	- [1]	116 (109)	103 (90)	115 (107)	86 (95)	- [1]	108 (91)	- [1]	112 (113)	- [1]	90 (102)
**1e**	100 (107)	- [1]	127 (93)	83 (94)	47 (108)	128 (119)	- [1]	114 (94)	- [1]	132 (107)	- [1]	116 (99)
**1g**	89 (103)	- [1]	83 (93)	93 (96)	118 (107)	139 (99)	- [1]	105 (97)	- [1]	125 (116)	- [1]	101 (97)
**2d**	109 (111)	- [1]	102 (102)	83 (99)	113 (109)	129 (114)	- [1]	147 (111)	- [1]	131 (101)	- [1]	110 (102)
**2e**	58 (92)	- [1]	98 (94)	90 (92)	130 (108)	181 (118)	- [1]	105 (116)	- [1]	86 (107)	- [1]	111 (114)
**2f**	107 (105)	- [1]	113 (106)	101 (90)	130 (107)	178 (128)	- [1]	122 (104)	- [1]	108 (108)	- [1]	130 (106)
**2g**	108 (101)	93 (101)	93 (69)	88 (108)	90 (99)	180 (125)	88 (101)	113 (94)	62 (95)	104 (106)	86 (88)	89 (107)
**2i**	- [1]	97 (81)	97 (56)	96 (67)	101 (114)	- [1]	64 (86)	101 (98)	- [1]	- [1]	- [1]	94 (96)
**2j**	- [1]	77 (99)	55 (74)	109 (105)	107 (107)	- [1]	83 (106)	106 (97)	- [1]	93 (91)	- [1]	59 (105)
**2k**	- [1]	93 (100)	72 (83)	**51** (88)	92 (112)	- [1]	95 (83)	97 (100)	- [1]	- [1]	- [1]	100 (95)
**2l**	- [1]	79 (96)	66 (87)	**50** (106)	72 (112)	- [1]	78 (95)	100 (110)	- [1]	- [1]	- [1]	73 (63)
**2m**	- [1]	101 (98)	59 (83)	77 (96)	86 (104)	- [1]	87 (90)	94 (101)	- [1]	86 (111)	- [1]	79 (83)
**2n**	- [1]	118 (100)	84 (69)	100 (107)	95 (97)	- [1]	74 (56)	101 (99)	- [1]	65 (78)	- [1]	82 (82)
**2o**	- [1]	100 (101)	78 (75)	74 (85)	103 (115)	- [1]	86 (94)	96 (109)	- [1]	69 (102)	- [1]	104 (106)
**2p**	- [1]	92 (114)	74 (76)	99 (108)	96 (102)	- [1]	59 (86)	103 (88)	- [1]	73 (57)	- [1]	101 (101)
**2q**	- [1]	110 (101)	71 (69)	81 (84)	98 (99)	- [1]	82 (80)	101 (104)	- [1]	80 (85)	- [1]	105 (95)
**2r**	- [1]	110 (99)	93 (84)	105 (90)	109 (113)	- [1]	114 (100)	108 (107)	- [1]	105 (117)	- [1]	120 (133)
**2s**	- [1]	99 (109)	85 (80)	102 (94)	95 (104)	- [1]	80 (89)	103 (105)	- [1]	91 (76)	- [1]	114 (81)
**2t**	- [1]	95 (107)	83 (92)	76 (113)	111 (112)	- [1]	98 (85)	109 (109)	- [1]	110 (89)	- [1]	109 (138)
**2u**	- [1]	103 (119)	80 (82)	110 (87)	98 (104)	- [1]	91 (84)	98 (103)	- [1]	77 (70)	- [1]	91 (87)
**2v**	- [1]	119 (113)	72 (72)	60 (78)	98 (107)	- [1]	85 (97)	103 (111)	- [1]	79 (72)	- [1]	118 (126)
**3h**	- [1]	103 (103)	77 (89)	106 (99)	92 (106)	- [1]	99 (102)	- [1]	60 (91)	- [1]	92 (99)	100 (110)

[1] Not performed.
